# Grind, shine and detect: mechanochemical synthesis of AIE-active polyaromatic amide and its application as molecular receptor of monovalent anions or nucleotides†

**DOI:** 10.1039/d4ra02129k

**Published:** 2024-04-23

**Authors:** Jakub S. Cyniak, Artur Kasprzak

**Affiliations:** a Faculty of Chemistry, Warsaw University of Technology Noakowskiego Str. 3 00-664 Warsaw Poland artur.kasprzak@pw.edu.pl

## Abstract

A mechanochemical synthesis of novel polyaromatic amide consisting of 1,3,5-triphenylbenzene and 1,1′,2,2′-tetraphenylethylene skeletons has been established. The designed mechanochemical approach using readily available and low-cost equipment allowed a twofold increase in reaction yield, a 350-fold reduction in reaction time and a significant reduction in the use of harmful reactants in comparison to the solution synthesis method. The parameters of Green Chemistry were used to highlight the advantages of the developed synthesis method over the solution-based approach. The title compound was found to exhibit attractive optical properties related to the Aggregation-induced emission (AIE) behaviour. Taking the advantage of AIE-active properties of the synthesized polyaromatic amide, its application as effective and versatile molecular receptor towards detection of monovalent anions, as well as bio-relevant anions – nucleotides, has been demonstrated. The values of the binding constants were at the satisfactory level of 10^4^, the detection limit values were low and ranged from 0.2 μM to 19.9 μM.

## Abstract 2

The mechanochemical synthesis of AIE-active amide, as well as the application of the title compound as molecular receptor of monovalent anions and nucleotides, are described.

## Graphical abstract

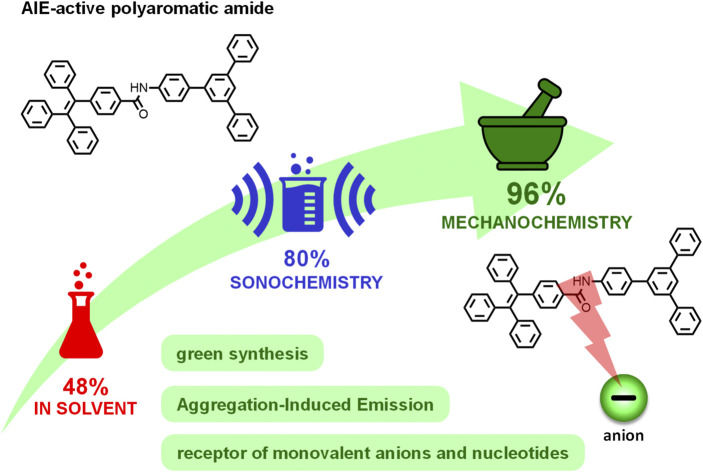

## Introduction

Conventional luminophores commonly suffer from fluorescence quenching caused by aggregation. Aggregation-induced emission (AIE)^1^ effect, described for the first time in 2001, has significantly changed the perception of the design of light-emitting materials.^2,3^ Fluorophores exhibiting AIE properties are characterised by weak emission in the molecularly dissolved state, while in the aggregated or solid state they emit light with great intensity. Such feature makes these compounds very promising molecules from the viewpoint of applied sciences. Over the past 20 years, various applications of AIE-active compounds were demonstrated, *e.g.*, in the fields of organic light-emitting diodes (OLED) technology,^4–7^ bio-imaging,^8–11^ photodynamic therapy,^12–14^ smart materials^15,16^ and molecular receptors.^17–19^ The latter application is particularly interesting. Compounds exhibiting the AIE effect have been used to detect metal cations and anions, explosives, gases and compounds of biological interest.^20–25^ The latter class of analytes can be considered especially important with nucleotides (esters of nucleosides and phosphoric acid) playing a number of important roles in the human body. They support the synthesis of RNA and lipids, are involved in enzymatic processes and they are necessary for various metabolic processes and energy flow in cells.^26–29^ Nucleotides have also been used as components of antiviral drugs, mainly against HIV and hepatitis.^30,31^ Although several classes of chemical receptors for nucleotide sensing have been described, these have been compounds with complex structures,^32,33^ and, other sophisticated models such as organometallic complexes,^34^ micelles,^35^ nanotubes^36^ or AIE-gene-DNA associates.^37^

**Fig. 1:**
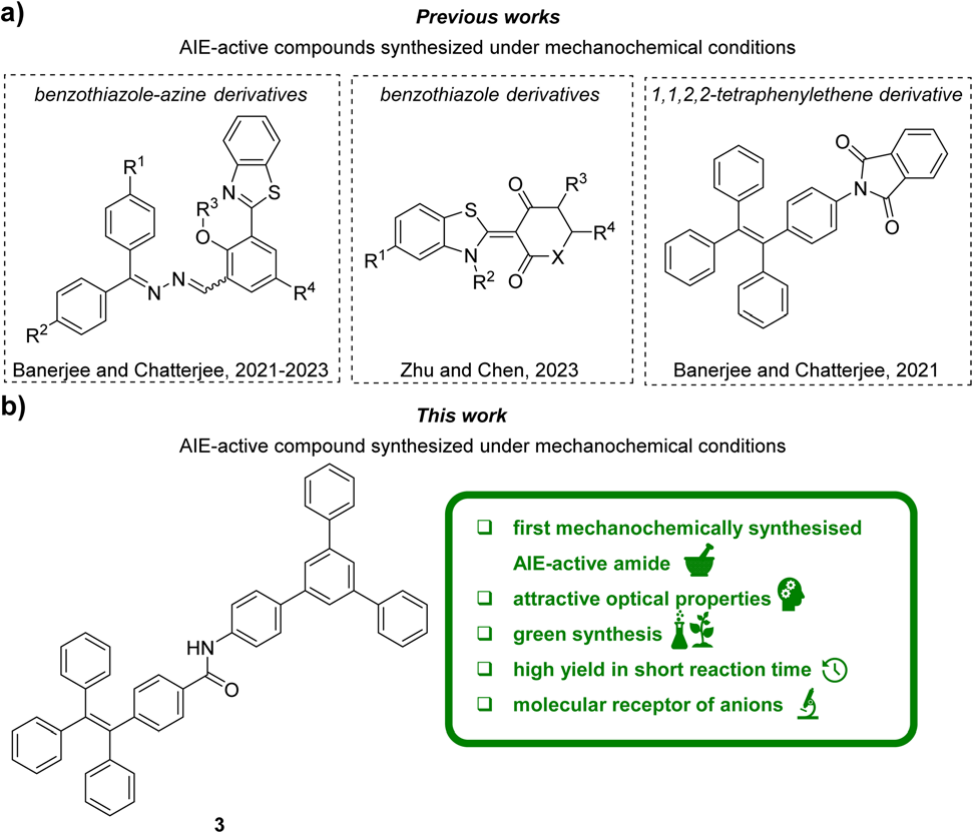
Graphical representation of the state of the art (a) and the contents of this work (b).

Mechanochemistry, although known since ancient times,^38^ is considered a modern and attractive synthetic tool in organic chemistry. The reduction in the use of harmful organic reactants and solvents, and thus the reduction in waste generation and notable reduction in reaction times are one of the most important advantages of mechanochemistry. Furthermore, despite the usage of simple and readily available instruments, mechanochemistry often allow synthesis of compounds, which are hard or even impossible to obtain by traditional solution-based methods.^39,40^ All these benefits make mechanochemistry one of the flagship methods of Green Chemistry, fulfilling many of its paradigms. Despite mechanochemical synthesis and design of AIE-active compounds are emerging fields of chemistry, the reports dealing with merging these concepts are extremely sparse.^41^ To the best of our knowledge, the reports mostly include very recent (2021–2023) studies on the grinding-induced synthesis of AIE-active benzothiazole-azine^42–44^ or benzothiazole derivatives^45^ (Fig. 1a). Despite 1,1′,2,2′-tetraphenylethylene skeleton is commonly used as first- or best-choice AIE-active unit,^8,24,25,46^ only one phthalimide derivative bearing this motif was recently synthesized under mechanochemical conditions^47^ (Fig. 1a). Neither other conjugates of 1,1′,2,2′-tetraphenylethylene nor the AIE-active 1,3,5-triphenylbenzene derivatives were obtained using mechanochemistry. Also, 1,1′,2,2′-tetraphenylethylene derivatives monosubstituted with 1,3,5-triphenylbenzene have not been studied extensively. To the best of our knowledge, only one such carbon–carbon linked conjugate, bearing the naphthalene linker, has been described in the patent (2005).^48^

In this work (Fig. 1b), the application of a mechanochemical method to synthesise a polyaromatic amide 3 consisting of 1,3,5-triphenylbenene and 1,1′,2,2′-tetraphenylethylene units is described for the first time. We demonstrated that the developed mechanochemical synthesis method provided excellent yields with shorter reaction times while significantly reducing usage of toxic reactants and solvents compared to the current state of the art. The parameters of Green Chemistry were used to highlight the advantages of the developed synthesis method over the conventional approach. The synthesized polyaromatic amide has been comprehensively characterised by multiple spectroscopic methods in terms of properties derived from the AIE effect, and then was applied as an innovative, versatile receptor for the detection of both monovalent and bio-relevant anions, that is nucleotides. Taking into account that the synthesis of 3 was performed with readily available and low-cost equipment, herein, we were able to show the power of using simple mechanochemical approaches for creation of attractive AIE-active compound toward the design of innovative organic molecular receptors.

## Results and discussion

### Synthesis

**Scheme 1:**
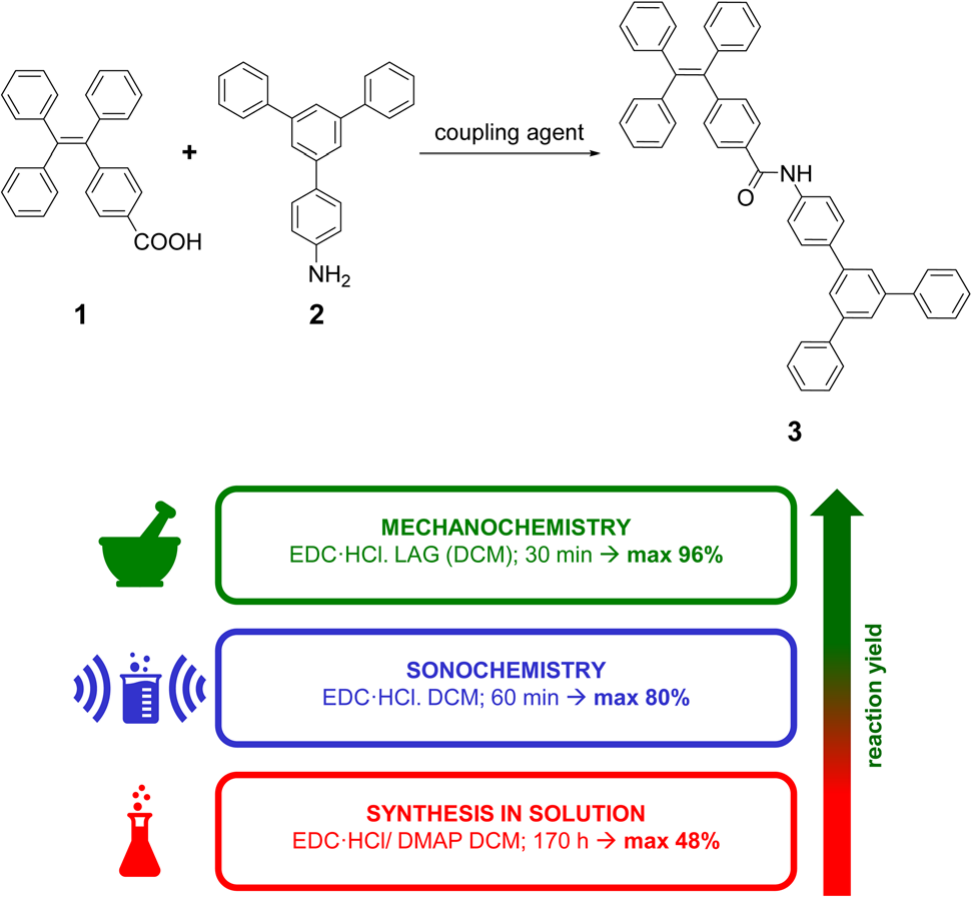
General synthesis path to obtain compound 3 together with the graphical presentation of key-results.

The general synthesis path of compound 3 is presented in Scheme 1 (for the full experimental details see sections S1.1–S1.4 in ESI†). We assumed that 3 can be synthesised *via* direct coupling of carboxylic acid 1 (4-(1,2,2-triphenylvinyl)benzoic acid) with an amine 2 (5′-phenyl-[1,1′:3′,1′′-terphenyl]-4-amine) in presence of a coupling agent.

Initial trials were focused on reactions carried out in various organic solvents in the presence of EDC·HCl (1-(3-dimethylaminopropyl)-3-ethylcarbodiimide hydrochloride) as a coupling agent (all starting materials as well as the product had good solubility in the solvents tested). These were intended to provide a reference point for further syntheses by mechanochemical and sonochemical methods, as well as to provide a benchmark for comparing reaction yields. To our surprise, these experiments provided unsatisfactory to moderate yields (9–48%), even for very long reaction times (up to 170 h). The usage of thionyl chloride also did not provide satisfactory yield (9%).

**Fig. 2:**
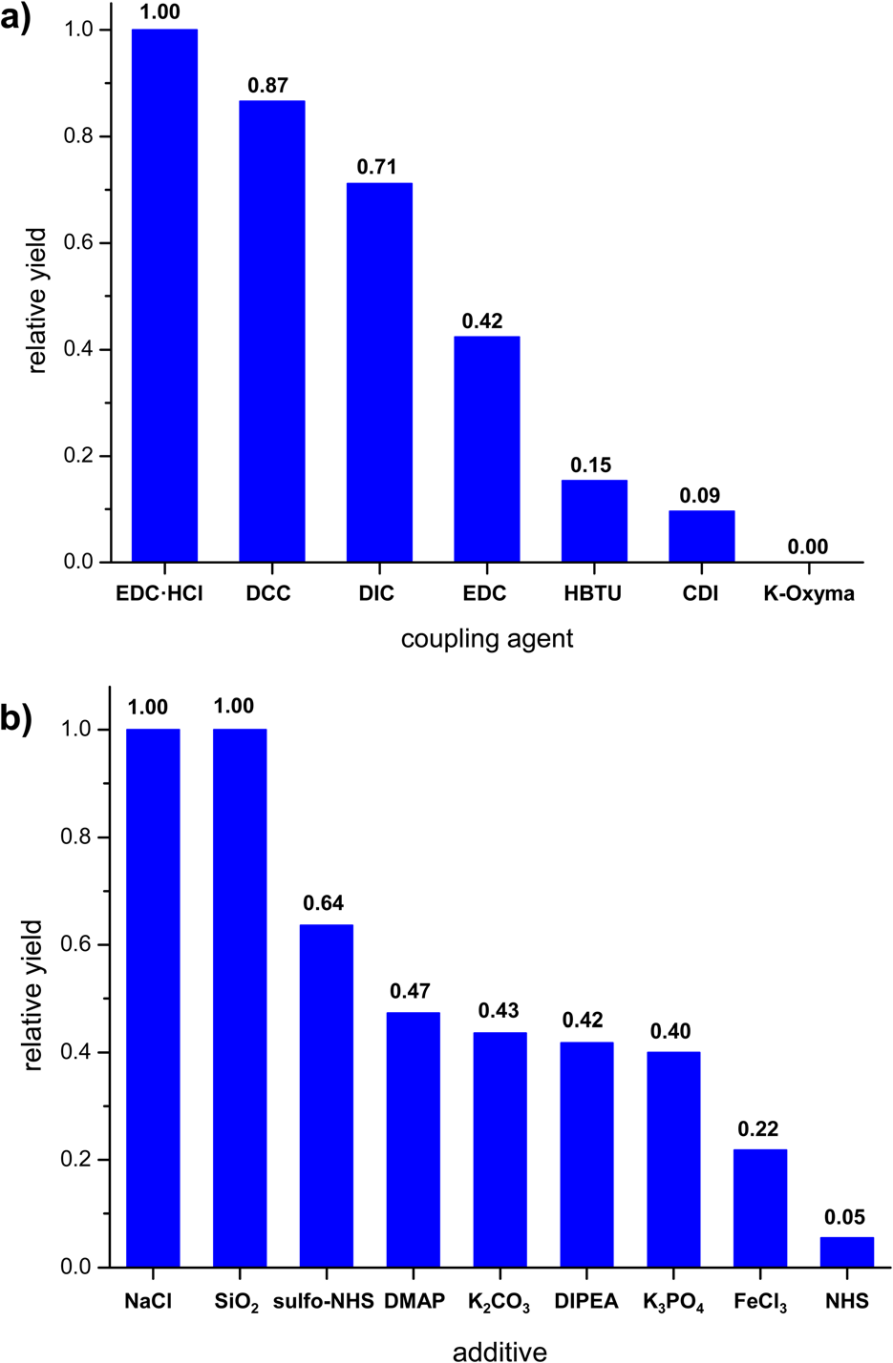
Effect of reaction parameters on the relative yield of mechanochemical synthesis (grinding in mortar); (a) coupling agent, (b) additives.

Having established that carrying out the reaction using solution method yielded poor or average results, we proceeded to attempt a mechanochemical synthesis of 3. Our ultimate goal was to perform the reaction using readily available and low-cost equipment, available in every organic chemistry laboratory. To our delight, just 15 minute grinding of compounds 1 and 2 in a hand-held mortar in the presence of EDC·HCl and small amount of DCM (50 μL) (LAG – Liquid Assisted Grinding) provided 3 with similar reaction yield (52%) as for the process in the solvent conducted for 170 hours, *i.e*., in the 350-fold less time. In the next steps, we evaluated whether other coupling agents or additives (grinding auxiliaries) would enable a further increase in reaction yield. For this purpose, reactions were carried out by grinding starting materials in hand-held mortar with different coupling agents or additives. The highest yields were obtained for carbodiimides (Fig. 2a). During grinding procedure, the mixture was a pale-yellow powder and did not change throughout the grinding process. Additives we used (Fig. 2b) differed in their acid-base nature. Experiments showed only a slight increase in reaction yield for compounds of neutral character (NaCl and SiO_2_). Alkaline and acidic additives generally caused a decrease in reaction yield. We also noted that the additives caused the form of the mixture to change to a dark yellow viscous paste, which made grinding more difficult. An important parameter for mechanochemical reactions is the presence of a small amount of solvent (LAG). To our delight, we found that it was possible to replace dichloromethane with ethyl acetate without any significant decrease in the reaction yield. The effect of grinding time on reaction yield was also investigated. The reaction mixture was ground in a hand-held mortar for 5 to 30 minutes. Increasing the grinding time did not significantly change the reaction yield.

Mechanochemical reactions can be carried out not only in mortars or specialised mills, but also using systems as simple as a glass rod and a narrow glass vial. There are reported examples of reactions for which such a simple set-up has allowed high yields to be obtained.^49^ To our delight, just a 15 minute grinding of the reactants with glass rod in glass vial provided target product with 84% yield. Further extension of the reaction time to 30 minutes provided an excellent 96% yield (for the optimised reaction protocol, see experimental section). During grinding, after about 3 minutes the mixture became a dark yellow viscous paste, which then solidified. The reaction carried out in ethyl acetate provided a slightly lower yield (80%), confirming the possibility of using this biodegradable solvent^50,51^ for the synthesis. This allows for a more sustainable synthesis with a slightly lower yield. We hypothesise that the differences in the yields obtained in the grinding reactions in the mortar and in the glass vial can be attributed to the method of the grinding. This might be due to the larger ratio of the surface area of the glass rod to that of the vial than the ratio of the surface area of the pestle to that of the mortar.

It is also worth noting that carrying out the reaction under sonochemical conditions did not provide higher yields (80%).

Finally, to check the repeatability of the designed grinding-induced protocol, we performed the mechanochemical synthesis of the target compound 3 under optimized mechanochemical conditions three times, at the similar scales and on different days (independent runs; grinding in glass vial with glass rod, reaction time: 30 minutes, 1.0 equiv. of EDCl). The synthesis outcomes were highly consistent what demonstrated that the designed grinding-induced synthesis is highly reproducible; the obtained isolated yields were consistent and equalled 93 ± 3%. ^1^H NMR analyses also supported the isolation of pure 3 in each synthesis run. Refer to ESI, subsection S1.3† for the full data regarding the reproducibility of the experiments.

### Green Chemistry metrics for the synthesis of 3

To generally compare and evaluate the designed methods of synthesising compound 3 – mechanochemically and in solvent – the relevant Green Chemistry metrics were used (Table 1). The calculated parameters were assessed by marking them with red, amber, and green flags according to Clark's unified metrics toolkit^52,53^ (for data and method of calculation, see ESI section S1.5†). A green flag was assigned to preferred values, an amber flag was assigned to values that were acceptable, but with some restrictions, and a red flag was assigned to undesirable values. Atom Economy (AE), Process Mass Intensity (PMI) and Reaction Mass Intensity (RMI) were defined following the literature.^52–54^

**Table 1:** Comparison of green metrics and reaction times for mechanochemical synthesis and synthesis in solvent

Metrics	Synthesis in solvent EDCl·HCl/DMAP	Mechanochemistry EDCl·HCl
Yield^a^ (%)	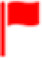	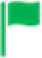
Reaction time (h)	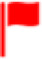	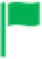
Atom economy (%)	67%	76%
RME (%)	72%	73%
PMI_(total)_^b^	5834	5689
PMI_(reaction)_^c^	154	9
PMI_(solvent)_^d^	153	1.9
PMI_(additive)_	0.037	0
Reaction solvent	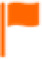	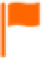
Health and safety^e^	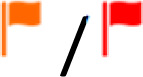	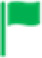
Main hazard statements^e^	H318, H370, H411	H302, H315, H335

a Isolated yields.

b Including coupling agent and workup solvents.

c Including additional reagent.

d For solvents used directly in synthesis.

e For additional safety data see ESI, section S1.5, Table S4.†

The closer the values of Atomic Economy (AE) and Reaction Mass Efficiency (RME) are to 100%, the more efficient is the reaction. On the other hand, the Process Mass Intensity (PMI) values should be in the lowest possible range. While the calculation of some parameters (*e.g*., PMI) are most direct for the reactions performed at the kilogram scale, which is not possible to conduct for every process at the design step and due to some laboratory (equipment and scale) limitations, we wanted to provide a general overview of the advantages of the designed mechanochemical approach as compared with the reaction in solution. In fact, some of the reported studies included the calculation of these parameters for the milligram scale reactions.^53^

The reaction yield of the mechanochemical reaction was double that of the reaction in solvent and was achieved in almost 350-fold shorter time. A higher Atom Economy (about 10%) was achieved in the mechanochemical reaction, due to the absence of an additional reactant (DMAP), which was used in the synthesis in solution. Similar values of Process Mass Intensity (PMI) and Reaction Mass Efficiency (RME) were obtained for both synthetic approaches. Differences can be seen when individual components of PMI are extracted. Mechanochemical reaction using in a lower volume of solvent (LAG) and without an additive resulted in a significant reduction in PMI_(reaction)_ and PMI_(solvent)_ values by approximately seventeen and eighty-one times respectively. Both methods use dichloromethane as a solvent, small amount used in mechanochemical synthesis reduces exposure to its hazardous properties (causes skin and eye irritation – H315 and H319, may cause drowsiness or dizziness – H336 and is suspected of causing cancer – H351).

### Analyses on the AIE behaviour of 3

**Fig. 3:**
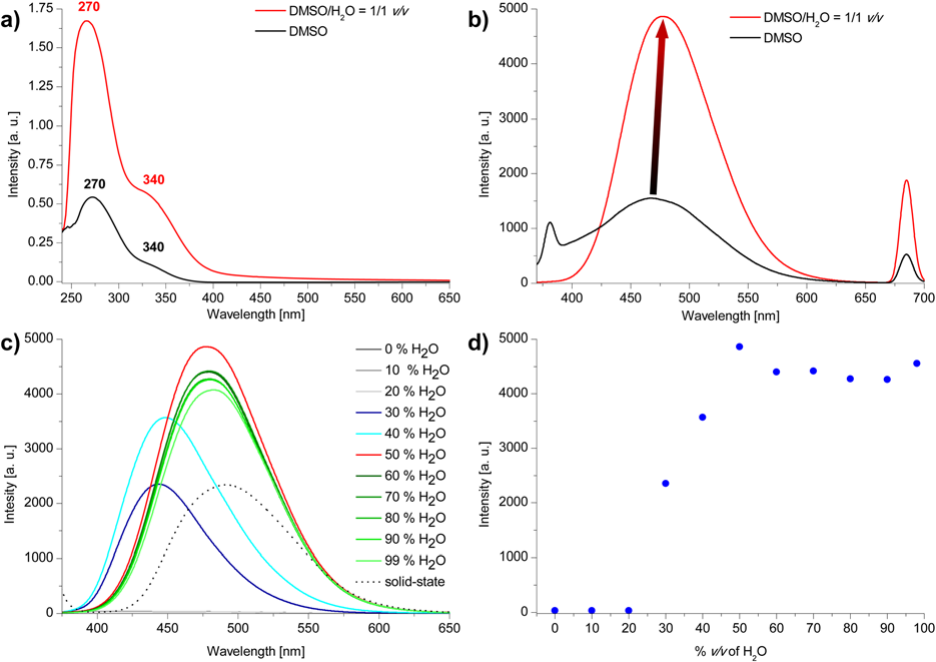
(a) UV-vis spectra (*C*_3_ = 2 × 10^−5^ M), (b) emission spectra (c) emission spectra of compound 3 in DMSO/H_2_O system (for b and c: *λ*_ex_ = 340 nm) (d) emission intensity dependence on the water content (vol%) in the DMSO/H_2_O system.

The structure of the compound was confirmed by ^1^H NMR and {^1^H}^13^C NMR spectroscopies, high-resolution mass spectrometry (HRMS) and elemental analysis. The UV-vis spectra of 3 shows strong absorption maximum (*λ*_max_) located at *ca.* 270 nm and weaker *λ*_max_ at *ca.* 340 nm (Fig. 3a). The emission spectra show differences depending on the value of the excitation wave. For *λ*_ex_ = 340 nm, a broad maximum is observed in the range *ca.* 400–540 nm (Fig. 3b, for all spectra see ESI, section S4†).

**Fig. 4:**
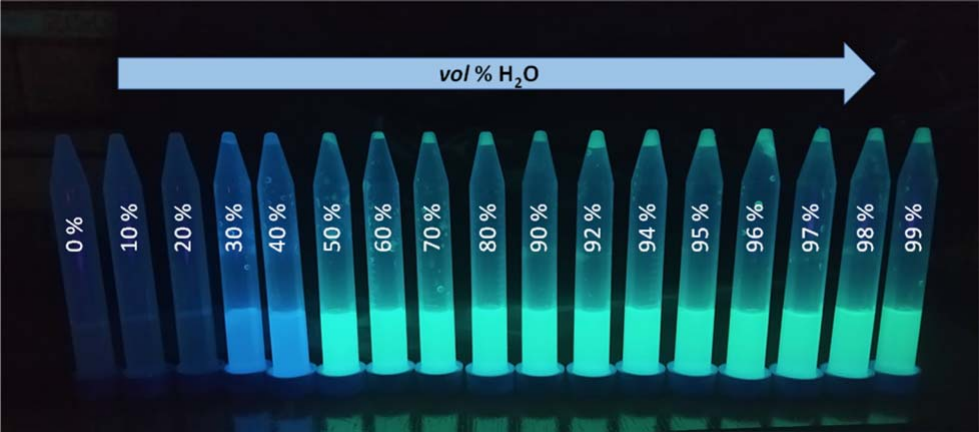
Aggregates of 3 in DMSO/H_2_O system containing different vol% of water in the sample seen under UV light (*λ*_ex_ = 365 nm).

Due to the presence of 1,3,5-triphenylbenzene and 1,1′,2,2′-tetraphenylethylene skeletons in the compound 3 structure we anticipated that this compound could feature AIE properties. The emission properties of compound 3 in the aggregated state were studied in the DMSO/H_2_O system with increasing volume of water (Fig. 3c and d). For water contents below 30 vol% emission is very weak. For 30–40 vol% water content an intensive blue emission of *λ*_max_*ca.* 445 nm appears, followed by a bathochromic shift of *λ*_max_ to *ca.* 465 nm from 50 vol% water to 99 vol% of water content in the sample. A maximum emission is observed for 50 vol% water. Further increase in the amount of water slightly reduces the emission intensity and causes it to settle at a relatively constant level. Changes in emission maximum and intensity can also be observed with the naked eye (Fig. 4). The emission intensity in DMSO/H_2_O = 1/1 v/v system is almost double that of the solid-state (Fig. 2c). We hypothesise that the occurrence of a maximum in emission intensity for 50% water content in the sample and the subsequent slight decrease in emission intensity for higher water contents might be related to the size of the aggregates. Increasing the percentage of water content in the sample to 90% results in a more than 9-fold decrease in the mean hydrodynamic diameter of the aggregates (from 254 nm to 27 nm; see DLS data in ESI, section S6†).

**Fig. 5:**
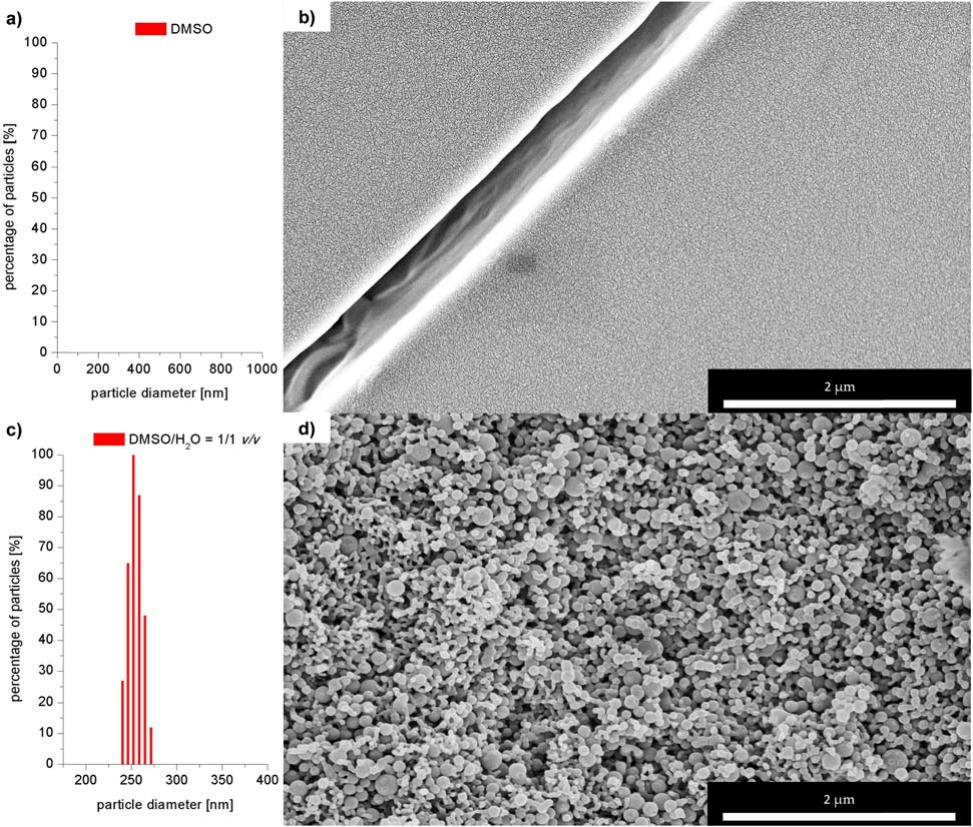
DLS measurements: size of particles of 3 in: (a) DMSO, (c) DMSO/H_2_O = 1/1 v/v; SEM images of 3; (b) solid obtained after column chromatography, (d) dried aggregates.

To further confirm the presence of compound 3 in the form of aggregates, DLS (Dynamic Light Scattering) analysis was performed. Importantly, no particles were detected in the DMSO solution of 3 (Fig. 5a). On the other hand, particles with a mean hydrodynamic diameter of 254 nm were found in the DMSO/H_2_O system, confirming the formation of aggregates (Fig. 5c). Interestingly, increasing the water content of the system from 50 vol% to 90 vol% resulted in a 27-fold reduction in the mean hydrodynamic diameter of the aggregates, to 27 nm. In order to demonstrate the differences in morphology of solid compound 3 in the aggregated form and before aggregation, SEM (Scanning Electron Microscope) images were acquired (see data on SEM in sections S1.1 and S7 in ESI†). Fig. 5b shows the SEM image of solid compound 3 obtained after purification on a chromatographic column. The image shows a smooth particle surface with a sharp boundary between grains – bright stripe on the upper left (the surface irregularities are due to the metallic gold layer applied to the sample and are not due to the surface morphology of the sample). Fig. 5d shows the SEM image of the aggregates of compound 3 obtained by filtrating them from the DMSO/H_2_O = 1/1 v/v solution and later drying on air at room temperature. A significant change in the morphology of the samples can be observed. Instead of a flat surface for solid compound 3, fine, spherical particles of various sizes (diameter <100 nm) are forming the entire volume of the sample of aggregated 3. The spheres are not completely merged, voids are visible between them. Three-dimensional sponge-like structure was formed. There are also areas where the spherical aggregates merge together to form elongated rod-shaped structures.

### Application of 3 as molecular receptor of monovalent anions

After successful synthesis of 3 we decided to investigate application of this compound as molecular receptor of monovalent anions. We anticipate that such supramolecular recognition would be enabled by the presence of amide moiety in the structure of 3. We also hypothesised that these interactions of AIE-active compound 3 shall be characterized by satisfactory binding parameters.

To confirm that the amide bond is involved in the interaction of 3 with the anions, a series of ^1^H NMR spectra were measured with increasing molar concentration of Br^−^ anion (in the form of tetrabutylammonium bromide) from 0 eq to 10 eq in solution (for experimental details and spectra see ESI sections S1.7.1 and S5.1,† respectively). An upfield chemical shift of the signal coming from the proton of the amide group was observed. We have attributed this phenomenon to the anion binding, namely the process of proton transfer from the amide group of the receptor to the anion. No changes were observed neither in the chemical shift of the other signals of compound 3 nor the tetrabutylammonium cation.

**Fig. 6:**
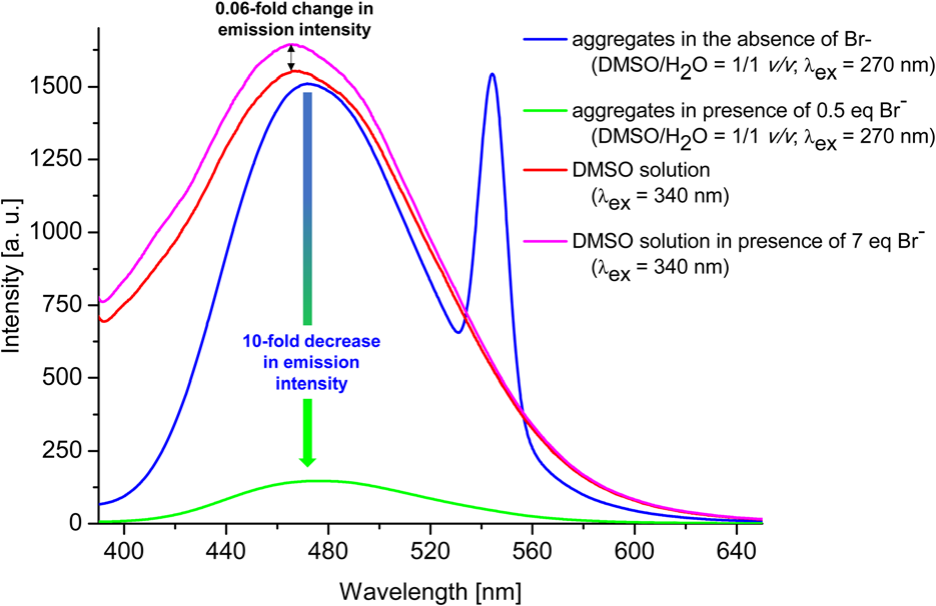
General comparison of emission intensity of 3 in different systems.

Having confirmed by 1H NMR spectroscopy the occurrence of non-covalent interactions of compound 3 with anions, they were further characterised for Br^−^ as the representative monovalent anion by spectrofluorimetry (*λ*_ex_ = 270 nm and 340 nm) in DMSO and DMSO/H_2_O = 1/1 v/v system (for experimental details see ESI, section S1.7.2†). Control experiments revealed that the most significant changes (decreases) in emission intensity, were observed for the emission spectra measured in DMSO/H_2_O = 1/1 v/v system and applying *λ*_ex_ of 270 nm. Therefore, this solvent system and excitation wavelength were used to further analyses. Fig. 6 shows a general comparison for the emission spectra of compound 3 in the presence or absence of Br^−^ in different solvent systems. The addition of 0.5 eq. of Br^−^ to compound 3 in the aggregated state (DMSO/H_2_O = 1/1 v/v) causes 10-fold decrease in the emission intensity (compare blue and green lines). On the other hand, for solution of 3 in DMSO (red line), only 0.06-fold increase in emission intensity was observed after the addition of 7 molar equivalents of Br^−^ (compare pink and red lines). This observation confirms our hypothesis that compound 3 in aggregated form exhibit better receptor properties than in the molecularly solubilised state.

**Fig. 7:**
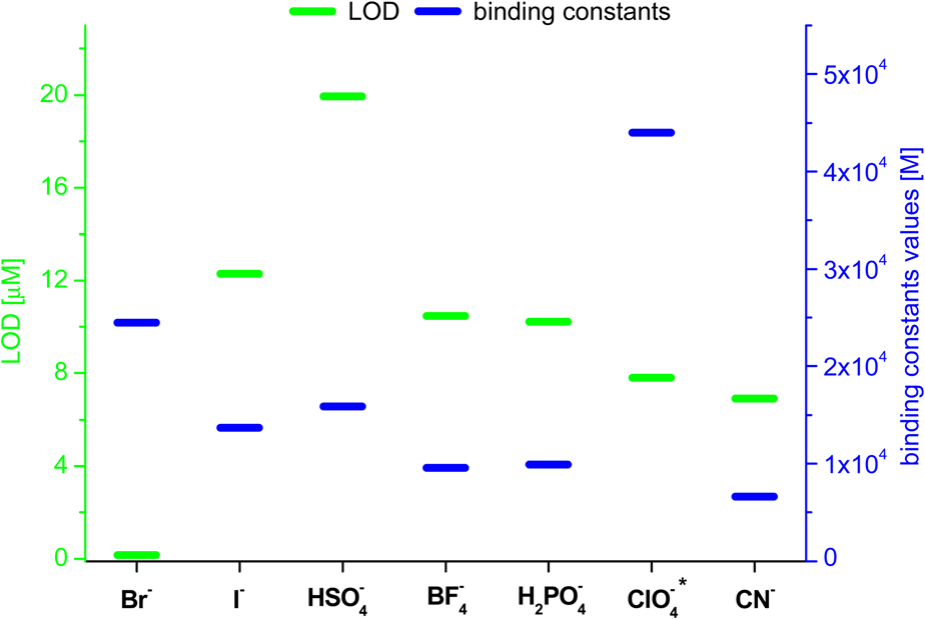
Values of LOD and binding constants for different anions; *determined by Benesi–Hildebrant method, for other by Stern–Volmer method.

Based on the results obtained for Br^−^, interactions of 3 with other monovalent anions (I^−^, HSO_4_^−^, BF_4_^−^, H_2_PO_4_^−^, ClO_4_^−^, CN^−^) were characterized by emission spectra in aggregated state (2 × 10^−5^ M, DMSO/H_2_O = 1/1 v/v, *λ*_ex_ = 270 nm; for the spectra see ESI, section S5.2†). For most of the analysed anions, decreases in emission intensity were observed, and only for ClO_4_^−^ ions there was an increase in emission intensity. The values of the binding constants were determined using the Stern–Volmer method (systems for which a decrease in emission intensity was observed; *K*_sv_ was calculated) and Benesi–Hildebrant method (systems for which an increase in emission intensity was observed; *K*_app_ was calculated) (for details see ESI, section S5†). The determined values of the binding constants were at the level of 10^4^ M. The highest value (4.4 × 10^4^ M) was obtained for ClO_4_^−^(Fig. 7, blue dashes). Limit of Detection (LOD) values were also determined, for anions showing an increase in emission on the basis of the Benesi–Hildebrant equation, and for anions showing a decrease in emission on the basis of the Stern–Volmer equation. The lowest value (0.17 μM) was obtained for the Br^−^ anion (Fig. 7, green dashes).

### Application of 3 as molecular receptor of bio-relevant anions – nucleotides

**Fig. 8:**
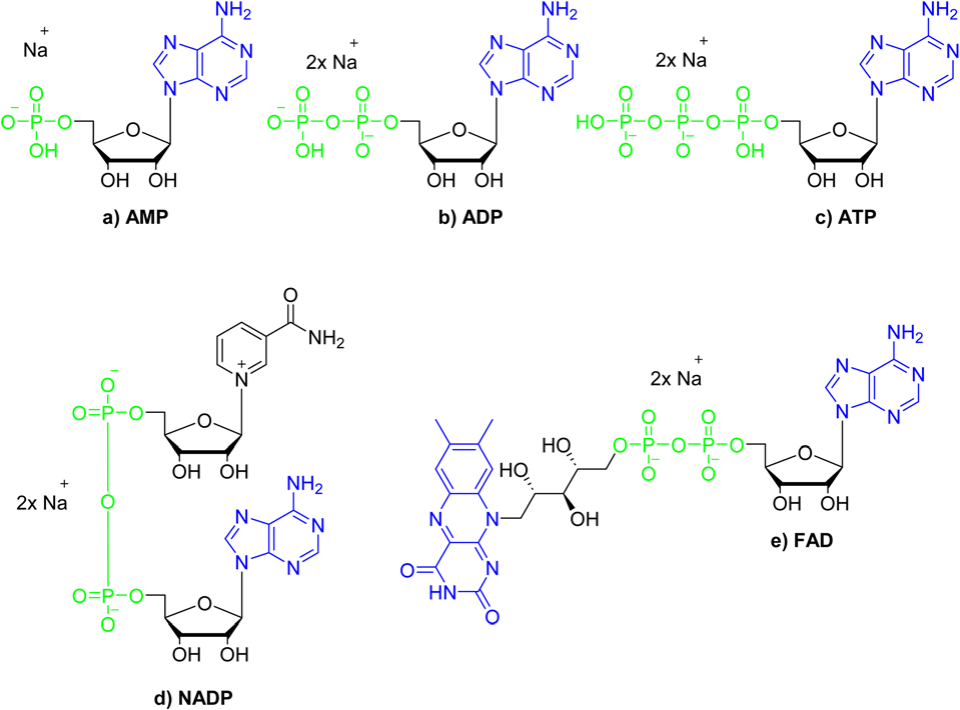
Structures of the nucleotides selected for the studies on interactions with compound 3; green colour denotes phosphate groups; blue colour denotes nucleobases moieties.

The chemical structures of nucleotides include the negatively charged phosphate moieties, what on the basis of the above-presented results, makes them attractive bio-relevant analytes within our studies. Five nucleotides varying in chemical structure were selected for the studies on interactions with compound 3 (Fig. 8). These include the monoanionic AMP (adenosine monophosphate), the dianionic ADP (adenosine diphosphate), NADP (nicotinamide adenine dinucleotide), FAD (flavin adenine dinucleotide) and the trianionic ATP (adenosine triphosphate). All compounds contain adenosine moiety in their structure. In addition, NADP and FAD are dinucleotides that are a combination of an adenosine nucleotide with a nicotinamide nucleotide, or flavin mononucleotide, respectively.

**Fig. 9:**
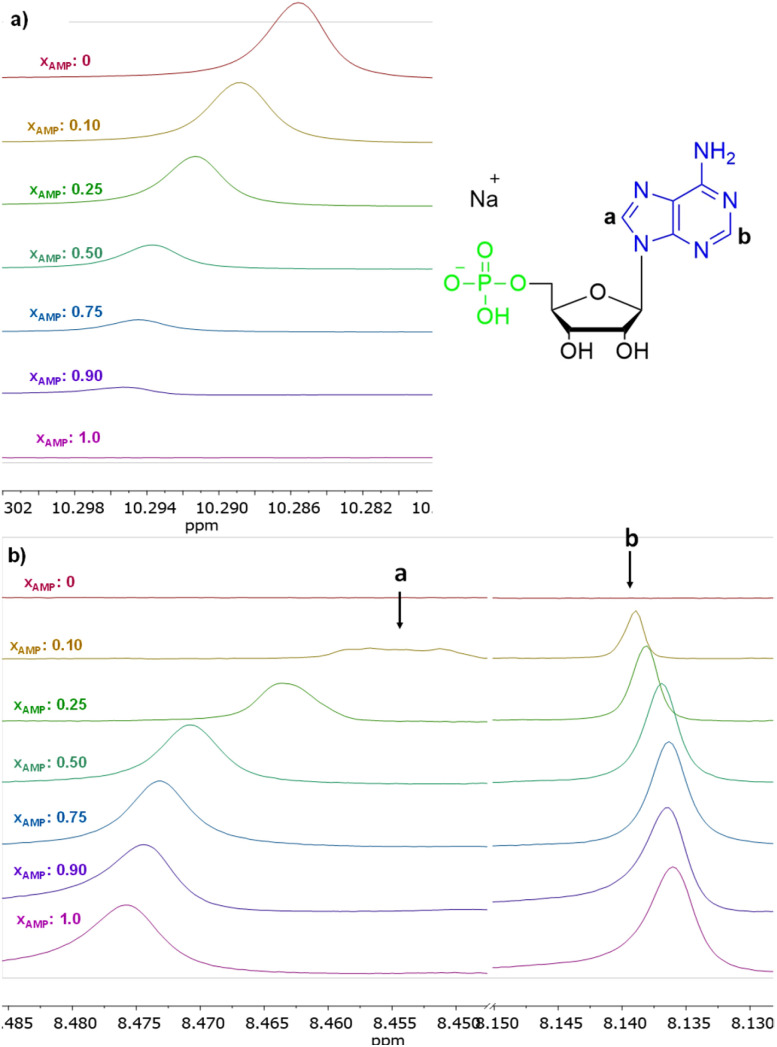
Insets of ^1^H NMR spectra (500 MHz, DMSO-*d*_6_) of 3 in the presence of various molar equivalents of AMP (a) shift of proton signal of amide group, (b) shift of proton signal of nucleotides nucleobase.

The interactions of 3 with representative nucleotides, namely AMP and ADP, were investigated using ^1^H NMR spectroscopy (for the full spectra see ESI, section S5.1†). Measurements were made while keeping total number of moles of receptor (3) and analyte (nucleotide) in the sample on the constant level, with varying their molar fractions. Fig. 9 shows the ^1^H NMR titration data for representative AMP. Shift of the signal of the amide group was observed (Fig. 9a). We have attributed this feature to the anion binding phenomenon, namely the process of proton transfer from the amide group of the receptor to the anion. Shifts in the signals of the nucleobase were also observed (protons a and b, Fig. 9b). The stoichiometry of the complexes formed was estimated using Job's plot method^55^ (continuous variation method; see details of this methodology in section S5, ESI†), was found to be 3 : 1 both for AMP and ADP, that is three molecules of amide (3) per one nucleotide. We hypothesised that, in the case of AMP, one molecule of the amide 3 binds to the phosphate group and two molecules bind to nitrogen atoms in the nucleobase (as indicated by chemical shifts on the ^1^H NMR spectrum) which means that this structural moiety is also involved in interaction with receptor 3. In the case of ADP, only one signal shift of the nucleobase is observed, which may indicate that two amide molecules bind to phosphate groups and one to the nucleobase.

**Fig. 10:**
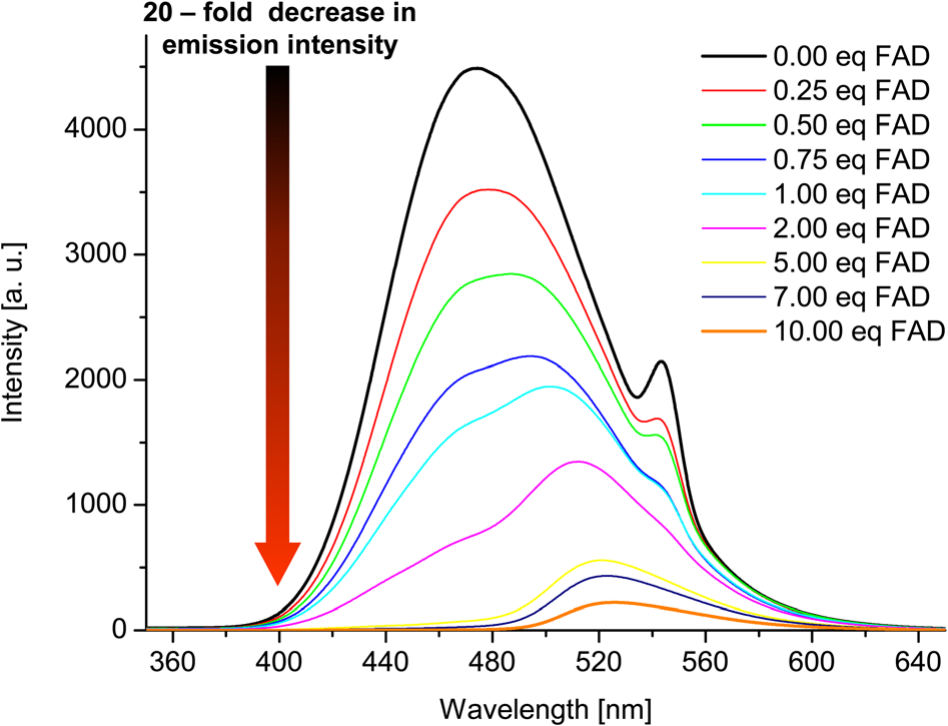
Emission spectra of 3 in the presence of various molar equivalents of FAD (DMSO/H_2_O 1 : 1 v/v, *C*_3_ = 2 × 10^−5^ M, *λ*_ex_ = 270 nm).

**Fig. 11:**
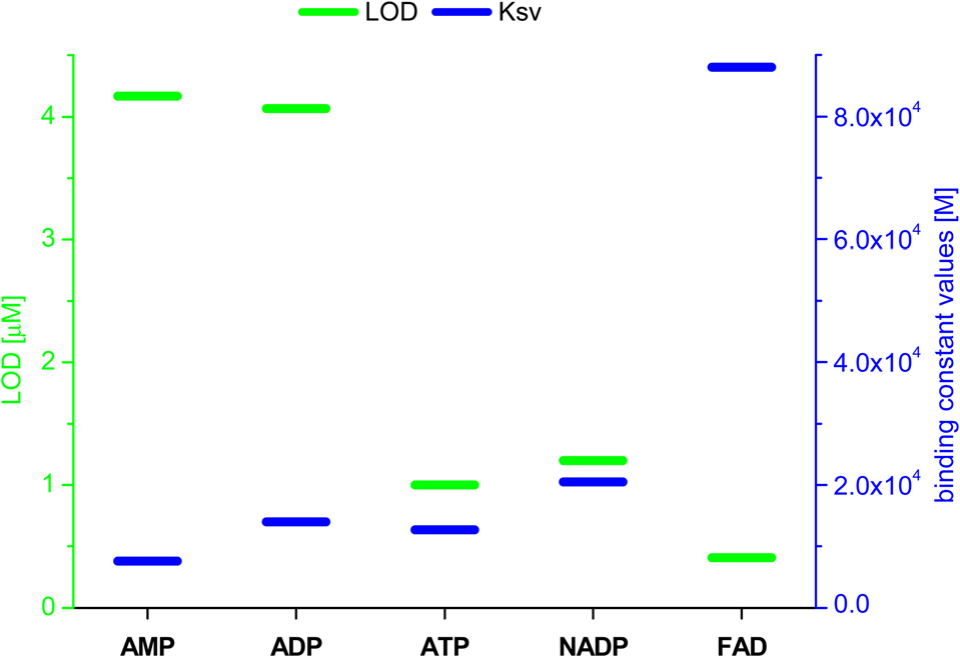
Values of LOD and binding constants (*K*_SV_) for different nucleotides.

Having confirmed by 1H NMR spectroscopy the occurrence of non-covalent interactions of compound 3 with anions, they were further characterised by emission spectra in aggregated state (2 × 10^−5^ M, DMSO/H_2_O = 1/1 v/v, *λ*_ex_ = 270 nm; see details of this methodology in SI, section S5.2†). For all nucleotides, a decrease in emission intensity was observed as their molar concentration in the sample increased. Fig. 10 shows emission spectra of 3 in the presence of various molar equivalents of FAD as representative nucleotide. Addition of 10 equivalents of FAD caused 20-fold decrease in emission intensity. The values of the binding constants were determined using the Stern–Volmer equation (*K*_sv_). The determined values of the binding constants were at level of 10^4^ M. These *K*_app_ values are in a good agreement with the values for monovalent anions. This means, that receptor 3 can not only effectively bind simple anions, but also sophisticated bio-anions, with the similar *K*_app_. The highest value (8.80 × 10^4^ M) was obtained for FAD (Fig. 11, blue dashes). Limit of Detection (LOD) values were determined based on the Stern–Volmer equation. The lowest value (0.69 μM) was obtained for the FAD (Fig. 11, green dashes). When analysing the values of the binding constants obtained for each nucleotide, attention could be drawn to the value of the binding constant for FAD, which is almost 10 times higher than the binding constant for AMP. It might be explained by comparing the structure of FAD with the other nucleotides. Analyses of the interaction of amide (3) with AMP and ADP by ^1^H NMR show that, in addition to the interaction with the phosphate groups, there is also an interaction between amide (3) and the nitrogen atoms of the nucleobase. FAD, being a dinucleotide composed of flavin and adenine moieties, has a much higher number of nitrogen atoms in its structure that can interact with the amide.

## Conclusions

In conclusion, we demonstrated that polyaromatic amide exhibiting aggregation-induced emission (AIE) properties can be effectively obtained mechanochemically with use of simple reaction set-up with readily available and low-cost equipment. Our method, meeting the principles of Green Chemistry, provided an excellent reaction yield (96%) while reducing synthesis time by the factor of 350 and minimizing the use of harmful reagents and solvents. We presented that the as synthesized polyaromatic amide with asymmetrical molecular architecture consisting of 1,3,5-triphenylbenzene and 1,1′,2,2′-tetraphenylethylene skeletons features attractive AIE behaviours, as supported with extensive spectroscopic analyses. Spectroscopic analyses confirmed that compound 3 in the aggregated form exhibited versatile properties as an anion receptor, not only towards detection of monovalent anions but also bio-relevant anions, namely nucleotides. This work not only provides new mechanochemical tools towards creation of AIE-active functional polyaromatic molecules towards their applications as molecular receptors, but also demonstrates the attractive possibilities and advantages of merging three interesting research areas, that is: mechanochemistry, design of AIE-active molecules and application of organic compounds as molecular receptors.

## Experimental section

### Materials and methods

Chemical reagents and solvents for the synthesis were commercially purchased and purified according to the standard methods, if necessary. Thin layer chromatography (TLC) was performed using Merck Silica gel 60 F254 plates. The NMR experiments were conducted using a Varian VNMRS 500 MHz spectrometer (^1^H at 500 MHz, ^13^C{^1^H} NMR at 125 MHz) equipped with a multinuclear *z*-gradient inverse probe head. The spectra were recorded at 25 °C and standard 5 mm NMR tubes were used. ^1^H and ^13^C chemical shifts (*δ*) were reported in parts per million (ppm) relative to the solvent signal, *i.e*., DMSO-*d*_6_: *δ*_H_ (residual DMSO) 2.50 ppm, *δ*_C_ (residual DMSO) 39.5 ppm. In the case of NMR spectra were analyzed with the MestReNova v12.0 software (Mestrelab Research S. L). ESI-HRMS (TOF) measurements were performed with a Q-Exactive ThermoScientific spectrometer. Elemental analyzes were performed using CHNS Elementar Vario EL III apparatus. Each elemental composition was reported as an average of two analyses. UV-vis measurements were performed with a WVR UV-1600PC spectrometer, with the spectral resolution of 2 cm^−1^. For the UV-Vis measurements, the wavelengths for the absorption maxima *λ*_max_ were reported in nm. Emission spectra were recorded with a HITACHI F-7100 FL spectrometer; parameters for the spectra of liquid samples (DMSO solution): scan speed: 1200 nm min^−1^, delay: 0.0 s, EX slit: 5.0 nm, EM slit: 5.0 nm, PMT voltage: 700 V; parameters for the spectra of samples of aggregates (DMSO/H_2_O solution in various proportions): scan speed: 1200 nm min^−1^, delay: 0.0 s, EX slit: 5.0 nm, EM slit: 5.0 nm, PMT voltage: 400 V parameters for the spectra of solid samples: scan speed: 1200 nm min^−1^, delay: 0.0 s, EX slit: 5.0 nm, EM slit: 5.0 nm, PMT voltage: 400 V. The wavelengths for the emission maxima (*λ*_em_) were reported in nm. SEM Field emission scanning electron microscope Helios 5 PFIB (Thermo Scientific) with the use of SE (secondary electron) detector. Dynamic light scattering (DLS) measurements were performed with Brookhaven Instruments Particle Size Analyser 90Plus.

### Optimized protocol for the mechanochemical synthesis of 3

4-(1,2,2-Triphenylvinyl) benzoic acid (1) (20.0 mg; 5.3 × 10^−5^ mol; 1.0 eq.), 5′-phenyl-[1,1′:3′,1′′-terphenyl]-4-amine (2) (17.1; 5.3 × 10^−5^ mol; 1.0 eq.) and 1-(3-dimethyl-aminopropyl)-3-ethylcarbodiimide hydrochloride (EDC·HCl) (10.0; 5.3 × 10^−5^ mol; 1.0 eq.) were grinded in glass vial with glass rod in presence of DCM (50 μL) at room temperature for 30 min. Then a 1 mol dm^−3^ hydrochloric acid solution was added to the reaction mixture (20 mL), and the crude product was extracted with CH_2_Cl_2_ (3 × 20 mL). Organic layers were combined, washed with water and brine. After drying with MgSO_4_ followed by filtration, volatiles were distilled off on a rotary evaporator. Finally, the product was purified using a column chromatography (SiO_2_, 2% hex/CH_2_Cl_2_) to provide the target compound 3 as a yellow solid (note: compound 3 can also be purified by column chromatography with 50% c-hex/AcOEt (*R*_f_ = 0.9)). The data on the repeatability of this synthesis are presented in ESI, subsection S1.3.†

^1^H NMR (DMSO-*d*_6_, 500 MHz, ppm), *δ*_H_ 10.29 (s, 1H) 7.91–7.85 (m, 11H), 7.78–7.76 (m, 2H), 7.53–7,50 (m, 4H), 7.43–7,40 (m, 2H), 7.20–7.12 (m, 11H), 7.04–7.00 (m, 6H); {^1^H}^13^C NMR (DMSO-*d*_6_, 125 MHz, ppm), 165.0, 146.7, 142.9, 142.8, 142.7, 141.6, 141.1, 140.2, 139.7, 138.9, 135.1, 132.6 × 2, 130.6, 128.9, 128.0, 127.9 × 2, 127.8, 127.7, 127.3 × 2, 127.2, 126.9, 126.7, 124.0 × 2, 120.6; HRMS (ESI) *m*/*z* [M]^+^ calcd for C_51_H_37_NO = 680.2948, found = 680.2942 *m*/*z*; elemental analysis: Anal. Calcd for C_51_H_37_NO: C, 90.1; H, 5.49; N. 2.06. Found: C, 89.86; H, 5.49; N, 2.08. *R*_f_ (2% hex/CH_2_Cl_2_) = 0.91.

### Analyses of the interactions between 3 and anions (monovalent, nucleotides) using emission spectroscopy

The anion binding experiments between compound 3 (receptor) and anions (analytes; Br^−^, I^−^, HSO_4_^−^, BF_4_^−^, H_2_PO_4_^−^, SCN^−^, ClO_4_^−^, CN^−^, AMP, NADP and FAD) were performed employing the emission spectra titration experiments. In all cases, tetrabutylammonium ([N(C_4_H_9_)_4_]^+^) salts of anions were used. The experiments were performed in the DMSO/H_2_O = 1 : 1 *v*/*v* system as follows. Stock solution of 3 (2 × 10^−3^ M) in DMSO was diluted with adequate volume of pure DMSO (to reach volume of 1 mL), followed by addition of H_2_O solution containing given anion (final molar concentration of anion was between 5 × 10^−6^ M and 2 × 10^−4^ M).

### Analyses of the interactions between 3 and anions (monovalent, nucleotides) using ^1^H NMR spectroscopy

The binding experiments between compound 3 (receptor) and anions (Br^−^, AMP and ADP) were performed employing the ^1^H NMR titration experiments. Tetrabutylammonium bromide ([N(C_4_H_9_)_4_]^+^) was used in these experiments. The experiments were performed in DMSO-*d*_6_ containing TMS (tetramethylsilane, 0.03% vol) as follows. To a stock solution of 3 (7.5 × 10^−3^ M) in DMSO-*d*_6_ a stock solution of analyte (7.5 × 10^−3^ M) in DMSO-*d*_6_ was added, followed by addition of DMSO-*d*_6_ to reach given molar concentration of analyte in the sample (in case of AMP and ADP, stock solution of 3 and stock solution of analyte were mixed in such a way that the sum of receptor's (3) and analyte's molar concentrations in the sample were on the constant level with varying their molar fractions). Final volume of the samples was 1 mL.

### Estimation of binding parameters

For anions for which a decrease in emission intensity was observed (I^−^, HSO_4_^−^, BF_4_^−^, H_2_PO_4_^−^, CN^−^, AMP, ADP, ATP, NADP, FAD) the Stern–Volmer constant values (*K*_sv_) were estimated using the Stern–Volmer method, given by the equation: 
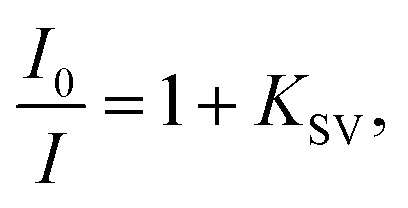
 where *I*_0_ and *I* are the fluorescence intensities of 3 in the absence and presence of given anion, respectively. *K*_SV_ were taken as a slope of 1/*C*(_*A*−_) *vs.* 1/Δ*I* linear plots. The limit of detection (LOD) values were estimated from the plot: of (*I* − *I*_min_)/(*I*_max_ − *I*_min_) *vs.* log([*A*^−^]). For ClO_4_^−^ where an increase in emission intensity was observed the apparent binding constant (*K*_app_) values were estimated using the Benesi–Hildebrand^56,57^ method, given by the equation: 
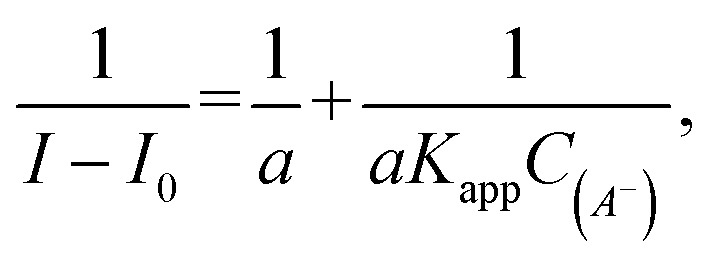
 where *I*_0_ and *I* are the fluorescence intensities of 3 in the absence and presence of given anion, respectively, *a* is a constant, and *C*_(*A*−)_ is the molar concentration of given anion in solution. *K*_app_ were determined as a ratio of intercept-to-slope of 1/(*I* − *I*_0_) *vs.* 1/*C*_(*A*−)_ linear plots. The data (for the estimation of *K*_app_ for the studied systems were collected from emission maxima (*λ*_em_) = 496 nm (*λ*_ex_ = 270 nm). The limit of detection (LOD) values were estimated by the equation: LOD = 3*S*/*b*, where *S* is standard error of intercept, and *b* is slope of regression line.

The stoichiometry of the complexes formed was estimated using Job's plot method (based on the ^1^H NMR experiments), from the plot: (1 − *x*) × (*δ* − *δ*_0_) *vs. x*. The *x* stands from the mole fraction of nucleotide. The expected stoichiometry was indicated by the maximum on the plot.

## Author contributions

J. S. C. performed most of the experiments, including all chemical syntheses and all receptor application studies, as well as collected, analyzed and processed all the analytic data (all under the supervision of A. K.). A. K. performed the experiments on the estimation of fluorescence quantum yields. A. K. and J. S. C. together designed the synthetic experiments. J. S. C. prepared all graphics for the manuscript and wrote the first manuscript draft. A. K. conceived the concept, supervised the whole project and experiments, analyzed all the data, provided funding acquisition, reviewed and edited the manuscript drafts, as well as led the correspondence with the reviewers. All authors discussed the results, commented on the manuscript and approved its final version.

## Conflicts of interest

There are no conflicts to declare.

## Supplementary Material

RA-014-D4RA02129K-s001
