# Supplementary material for: Grind, shine and detect: mechanochemical synthesis of AIE-active polyaromatic amide and its application as molecular receptor of monovalent anions or nucleotides
Source: RSC Adv. 2024 Apr 23;14(19):13227–36. doi: 10.1039/d4ra02129k (PMC11037028; doi:10.1039/d4ra02129k)
Supplement: RA-014-D4RA02129K-s001 [file RA-014-D4RA02129K-s001.pdf]

Electronic Supplementary Material (ESI) for RSC Advances.
This journal is © The Royal Society of Chemistry 2024

Electronic Supplementary Information (ESI) for

Grind, shine and detect: Mechanochemical synthesis of AIE-active polyaromatic amide and its application as molecular receptor of monovalent anions or nucleotides

Jakub S. Cyniak[a], Artur Kasprzak[a]*

[a] Faculty of Chemistry, Warsaw University of Technology, Noakowskiego Str. 3, 00-664 Warsaw, Poland

* Corresponding author e-mail: artur.kasprzak@pw.edu.pl

Table of contents:

List of figures ... 3
List of abbreviations: ... 5
S1. Experimental section ... 6
S1.1 Materials and methods ... 6
S1.2 Synthesis of compound **3** – synthesis in solvent ... 7
S1.3 Synthesis of compound **3** – mechanochemistry ... 8
S1.4 Synthesis of compound **3** – sonochemistry ... 11
S1.5 Green chemistry metrics ... 12
S1.6 Characterisation of aggregation induced emission effect ... 17
S1.7 Anion binding experiments ... 17
S1.7.1 Spectrofluorimetry ... 17
S1.7.2 ^{1}H NMR spectroscopy ... 17
S1.9 Estimation of fluorescence quantum yield ... 17
S2. NMR spectra ... 19
S3 HRMS spectrum ... 21
S4. Absorption and emission spectra ... 22
S5. Anions binding experiments ... 26
S5.1 Emission spectra ... 27
S5.2 ^{1}H NMR spectroscopy ... 27
S7. DLS measurements ... 51
S8 SEM images ... 53
S9 Supporting references ... 54

List of figures

Figure S 1 ^{1}H NMR (500 MHz, DMSO-d$_6$) spectrum of **3** 19
Figure S 2 {^{1}H}^{13}C NMR (125 MHz, DMSO-*d*$_6$) spectrum of **3** 19
Figure S 3 ^{1}H-^{1}H COSY NMR (500 MHz, DMSO-d$_6$) spectrum of **3** 20
Figure S 4 ESI-HRMS (TOF) spectrum of **3** 21
Figure S 5 UV-vis spectrum of compound **3** (DMSO, $C_3 = 2\cdot10^{-5}$ M) 22
Figure S 6 Emission spectra of **3** (DMSO, $C_3 = 2\cdot10^{-5}$ M, λ_{ex} = 270 nm (top), λ_{ex} = 340 nm (bottom)) 23
Figure S 7 3D emission spectrum of **3** (DMSO, $C_3 = 2\cdot10^{-5}$ M) 24
Figure S 8 Emission spectra of compound **3** in DMSO/H_2O system containing different vol% of water in the sample ($C_3 = 2\cdot10^{-5}$ M,) 24
Figure S 9 Emission spectra (λ_{ex} = 270 nm) of compound **3** in DMSO/H_2O system containing different vol% of water in the sample ($C_3 = 2\cdot10^{-5}$ M) 25
Figure S 10 3D emission spectra of **3** (DMSO/H_2O = 1:1 *v/v*, $C_3 = 2\cdot10^{-5}$ M) 25
Figure S 11 Emission spectra (λ_{ex} = 340 nm) of **3** in the presence of various molar equivalents of Br^- (DMSO/H_2O 1:1 *v/v*, $C_3 = 2\cdot10^{-5}$ M, λ_{ex} = 270 nm). 33
Figure S 12 Stern-Volmer plot regarding the interactions between **3** and **Br^-**. The data for the linear plot are also presented 34
Figure S 13 Plot for $(I-I_{min})/(I_{max}-I_{min})$ versus log(C_{Br-}) regarding the interactions between **3** and **Br^-**. The data for the linear plot are also presented. 34
Figure S 14 Emission spectra of **3** in the presence of various molar equivalents of **I^-** (DMSO/H_2O 1:1 *v/v*, $C_3 = 2\cdot10^{-5}$ M, λ_{ex} = 270 nm). 35
Figure S 15 Stern-Volmer plot regarding the interactions between **3** and **I^-**. The data for the linear plot are also presented 35
Figure S 16 Plot for $(I-I_{min})/(I_{max}-I_{min})$ versus log(C_{I^-}) regarding the interactions between **3** and **I^-**. The data for the linear plot are also presented. 36
Figure S 17 Emission spectra of **3** in the presence of various molar equivalents of **HSO_4^-** (DMSO/H_2O 1:1 *v/v*, $C_3 = 2\cdot10^{-5}$ M, λ_{ex} = 270 nm). 36
Figure S 18 Stern-Volmer plot regarding the interactions between **3** and **HSO_4^-**. The data for the linear plot are also presented. 37
Figure S 19 Plot for $(I-I_{min})/(I_{max}-I_{min})$ versus log(C_{HSO4-}) regarding the interactions between **3** and **HSO_4^-**. The data for the linear plot are also presented. 37
Figure S 20 Emission spectra of **3** in the presence of various molar equivalents of **BF_4^-** (DMSO/H_2O 1:1 *v/v*, $C_3 = 2\cdot10^{-5}$ M, λ_{ex} = 270 nm). 38
Figure S 21 Stern-Volmer plot regarding the interactions between **3** and **BF_4^-**. The data for the linear plot are also presented. 38
Figure S 22 Plot for $(I-I_{min})/(I_{max}-I_{min})$ versus log(C_{BF4-}) regarding the interactions between **3** and **BF_4^-**. The data for the linear plot are also presented. 39
Figure S 23 Emission spectra of **3** in the presence of various molar equivalents of **$H_2PO_4^-$** (DMSO/H_2O 1:1 *v/v*, $C_3 = 2\cdot10^{-5}$ M, λ_{ex} = 270 nm). 39
Figure S 24 Stern-Volmer plot regarding the interactions between **3** and **$H_2PO_4^-$**. The data for the linear plot are also presented. 40
Figure S 25 Plot for $(I-I_{min})/(I_{max}-I_{min})$ versus log(C_{H2PO4-}) regarding the interactions between **3** and **$H_2PO_4^-$**. The data for the linear plot are also presented. 40
Figure S 26 Emission spectra of **3** in the presence of various molar equivalents of **ClO_4^-** (DMSO/H_2O 1:1 *v/v*, $C_3 = 2\cdot10^{-5}$ M, λ_{ex} = 270 nm). 41

Figure S 27 Benesi-Hildebrand plots regarding the interactions between **3** and **ClO_4^-**. The data for the linear plot are also presented.. 41
Figure S 28 Emission spectra of **3** in the presence of various molar equivalents of **CN^-** (DMSO/H_2O 1:1 *v/v*, $C_3 = 2{\cdot}10^{-5}$ M, λ_{ex} = 270 nm). 42
Figure S 29 Stern-Volmer plot regarding the interactions between **3** and **CN^-**. The data for the linear plot are also presented. 42
Figure S 30 Plot for $(I-I_{min})/(I_{max}-I_{min})$ versus log(CCN −) regarding the interactions between **3** and **CN^-**. The data for the linear plot are also presented........................ 43
Figure S 31 Emission spectra of **3** in the presence of various molar equivalents of **AMP** (DMSO/H_2O 1:1 *v/v*, $C_3 = 2{\cdot}10^{-5}$ M, λ_{ex} = 270 nm). 43
Figure S 32 Stern-Volmer plot regarding the interactions between **3** and **AMP**. The data for the linear plot are also presented. 44
Figure S 33 Plot for $(I-I_{min})/(I_{max}-I_{min})$ versus $\log(C_{AMP})$ regarding the interactions between **3** and **AMP**. The data for the linear plot are also presented...................... 44
Figure S 34 Emission spectra of **3** in the presence of various molar equivalents of **ADP** (DMSO/H_2O 1:1 *v/v*, $C_3 = 2{\cdot}10^{-5}$ M, λ_{ex} = 270 nm). 45
Figure S 35 Stern-Volmer plot regarding the interactions between **3** and **ADP**. The data for the linear plot are also presented. 45
Figure S 36 Plot for $(I-I_{min})/(I_{max}-I_{min})$ versus $\log(C_{AMP})$ regarding the interactions between **3** and **ADP**. The data for the linear plot are also presented. 46
Figure S 37 Emission spectra of **3** in the presence of various molar equivalents of **ATP** (DMSO/H_2O 1:1 *v/v*, $C_3 = 2{\cdot}10^{-5}$ M, λ_{ex} = 270 nm). 46
Figure S 38 Stern-Volmer plot regarding the interactions between **3** and **ATP**. The data for the linear plot are also presented. 47
Figure S 39 Plot for $(I-I_{min})/(I_{max}-I_{min})$ versus $\log(C_{AMP})$ regarding the interactions between **3** and **ATP**. The data for the linear plot are also presented....................... 47
Figure S 40 Emission spectra of **3** in the presence of various molar equivalents of **NADP** (DMSO/H_2O 1:1 *v/v*, $C_3 = 2{\cdot}10^{-5}$ M, λ_{ex} = 270 nm). 48
Figure S 41 Stern-Volmer plot regarding the interactions between **3** and **NADP**. The data for the linear plot are also presented. 48
Figure S 42 Plot for $(I-I_{min})/(I_{max}-I_{min})$ versus $\log(C_{AMP})$ regarding the interactions between **3** and **NADP**. The data for the linear plot are also presented. 49
Figure S 43 Emission spectra of **3** in the presence of various molar equivalents of **FAD** (DMSO/H_2O 1:1 *v/v*, $C_3 = 2{\cdot}10^{-5}$ M, λ_{ex} = 270 nm). 49
Figure S 44 Stern-Volmer plot regarding the interactions between **3** and **FAD**. The data for the linear plot are also presented. 50
Figure S 45 Plot for $(I-I_{min})/(I_{max}-I_{min})$ versus $\log(C_{AMP})$ regarding the interactions between **3** and **NADP**. The data for the linear plot are also presented. 50
Figure S 46 ^{1}H NMR (500 MHz, DMSO-d$_6$) spectrum of **3** in presence of various molar equivalents of Br^- 27
Figure S 47 Inset of the ^{1}H NMR (500 MHz, DMSO-d6) spectrum of **3** in presence of various molar equivalents of Br^-.. 27
Figure S 48 Chemical formula of adenosine monophosphate (**AMP**) with the marked protons (a, b) for which the shifts of signals were observed in the ^{1}H NMR spectrum.. 28
Figure S 49 ^{1}H NMR (500 MHz, DMSO-*d*$_6$) spectrum of **3** in presence of various molar equivalents of **AMP** (grey colour indicates signals that are shifted)........... 28
Figure S 50 Insets of the ^{1}H NMR (500 MHz, DMSO-*d*$_6$) spectrum of **3** in presence of various molar equivalents of **AMP** 29

Figure S 51 Insets of the ^{1}H NMR (500 MHz, DMSO-d_6) spectrum of **3** in presence of various molar equivalents of **AMP** (amplification of the shifted proton signals of the nucleobase of the nucleotide) 29
Figure S 52 Job's plot regarding the interactions between **3** and **AMP** (the red arrow indicates the estimated stoichiometry of the complex formed).................. 30
Figure S 53 Chemical formula of adenosine diphosphate (**ADP**) with the marked protons (a, b), whose signals are shifted on the ^{1}H NMR spectrum....................... 30
Figure S 54 ^{1}H NMR (500 MHz, DMSO-d_6) spectrum of **3** in presence of various molar equivalents of **ADP** (grey colour indicates signals that are shifted) 31
Figure S 55 Insets of the ^{1}H NMR (500 MHz, DMSO-d_6) spectrum of **3** in presence of various molar equivalents of **ADP** (amplification of the shifted proton signals of the amide group)... 31
Figure S 56 Insets of the ^{1}H NMR (500 MHz, DMSO-d_6) spectrum of **3** in presence of various molar equivalents of **ADP** (amplification of the shifted proton signals of the nucleobase of the nucleotide) 32
Figure S 57 Job's plot regarding the interactions between **3** and **ADP** (the red arrow indicates the estimated stoichiometry of the complex formed).................. 32
Figure S 58 Size distribution pattern of **3** in DMSO 51
Figure S 59 Size distribution pattern of **3** in DMSO/H_2O = 1/1 *v/v* system 51
Figure S 60 Size distribution pattern of **3** in DMSO/H_2O = 9/1 *v/v* system 52
Figure S 61 SEM image of solid **3** obtained after column chromatography.................. 53
Figure S 62 SEM image of dried aggregates of **3** 53

List of abbreviations:

- **EDCI:** 1-(3-Dimethylaminopropyl)-3-ethylcarbodiimide hydrochloride
- **DMAP:** *N,N*-Dimethylpyridin-4-amine
- **k-Oxyma:** (Z)-ethyl 2-cyano-3-hydroxyacrylate potassium salt
- **NHS:** *N*-hydroxysuccinimide
- **sulfo-NHS:** *N*-hydroxy- sulfosuccinimide
- **CDI:** 1,1'-Carbonyldiimidazole
- **HBTU:** 2-(1H-benzotriazol-1-yl)-1,1,3,3-tetramethyluronium hexafluorophosphate
- **EDC:** 1-(3-Dimethylaminopropyl)-3-ethylcarbodiimide
- **DIC:** *N,N′*-Diisopropylcarbodiimide
- **DCC:** *N,N′*-Dicyclohexylcarbodiimide
- **AMP:** adenosine monophosphate
- **ADP:** adenosine diphosphate
- **ATP:** adenosine triphosphate
- **NADP:** nicotinamide adenine dinucleotide phosphate
- **FAD:** flavin adenine dinucleotide
- **RT:** room temperature
- **h:** hours

S1. Experimental section

S1.1 Materials and methods

Chemical reagents and solvents for the synthesis were commercially purchased and purified according to the standard methods, if necessary. Thin layer chromatography (TLC) was performed using Merck Silica gel 60 F254 plates.

The NMR experiments were conducted using a Varian VNMRS 500 MHz spectrometer (^1H at 500 MHz, $^{13}C\{^1H\}$ NMR at 125 MHz) equipped with a multinuclear z-gradient inverse probe head. The spectra were recorded at 25 °C and standard 5 mm NMR tubes were used. ^1H and ^{13}C chemical shifts (δ) were reported in parts per million (ppm) relative to the solvent signal, *i.e.*, DMSO-d_6: δ_H (residual DMSO) 2.50 ppm, δ_C (residual DMSO) 39.5 ppm. In the case of NMR spectra were analyzed with the MestReNova v12.0 software (Mestrelab Research S.L).

ESI-HRMS (TOF) measurements were performed with a Q-Exactive ThermoScientific spectrometer.

Elemental analyzes were performed using CHNS Elementar Vario EL III apparatus. Each elemental composition was reported as an average of two analyses.

UV-vis measurements were performed with a WVR UV-1600PC spectrometer, with the spectral resolution of 2 cm^{-1}. For the UV-Vis measurements, the wavelengths for the absorption maxima λ_{max} were reported in nm.

Emission spectra were recorded with a HITACHI F-7100 FL spectrometer; parameters for the spectra of liquid samples (DMSO solution): scan speed: 1200 nm/min, delay: 0.0 s, EX slit: 5.0 nm, EM slit: 5.0 nm, PMT voltage: 700 V; parameters for the spectra of samples of aggregates (DMSO/H_2O solution in various proportions): scan speed: 1200 nm/min, delay: 0.0 s, EX slit: 5.0 nm, EM slit: 5.0 nm, PMT voltage: 400 V parameters for the spectra of solid samples: scan speed: 1200 nm/min, delay: 0.0 s, EX slit: 5.0 nm, EM slit: 5.0 nm, PMT voltage: 400 V. The wavelengths for the emission maxima (λ_{em}) were reported in nm.

SEM Field emission scanning electron microscope Helios 5 PFIB (Thermo Scientific) with the use of SE (secondary electron) detector.

Dynamic light scattering (DLS) measurements were performed with Brookhaven Instruments Particle Size Analyser 90Plus

For grinding in hand-held mortar agate mortar with pestle was used (mortar diameter: 62 mm, pestle diameter: 24 mm)

For grinding in glass vial a vial (diameter: 18 mm) and rod (diameter: 8 mm) made of borosilicate glass were used.

For sonochemical reactions (Bandelin Sonorex RK 100H ultrasonic probe; ultrasonic peak output/HF power: 320W/80W; 35kHz) was used.

S1.2 Synthesis of compound 3 – synthesis in solvent

General method for the synthesis of compound 3 in solvent

In a round-bottom flask, 4-(1,2,2-triphenylvinyl) benzoic acid (**1**) was placed. Then 5′-phenyl-[1,1′:3′,1″-terphenyl]-4-amine (**2**) was added, followed by an addition of coupling agent and an organic solvent (see Table S1). The reaction mixture was stirred at room temperature under an argon atmosphere. Then, a 1 mol·dm^{-3} hydrochloric acid solution was added to the reaction mixture, and the crude product was extracted with CH_2Cl_2 (3x20 mL). Organic layers were combined, washed with water and brine. After drying with $MgSO_4$ followed by filtration, volatiles were distilled off on a rotary evaporator. Finally, the product was purified using a column chromatography (SiO_2, 2% hex/CH_2Cl_2) to provide the target compound **3** as a yellow solid (*Note: Compound 3 can also be purified by column chromatography with 50% c-hex/AcOEt, Rf = 0.9*).

^{1}H NMR (DMSO-*d*$_6$, 500 MHz, ppm), δ_H 10.29 (s, 1H) 7.91-7.85 (m, 11H), 7.78-7.76 (m,2H), 7.53-7,50 (m, 4H), 7.43-7,40 (m, 2H), 7.20-7.12 (m, 11H), 7.04-7.00 (m, 6H); {^{1}H}^{13}C NMR (DMSO-*d*$_6$, 125 MHz, ppm), 165.0, 146.7, 142.9, 142.8, 142.7, 141.6, 141.1, 140.2, 139.7, 138.9, 135.1, 132.6x2, 130.6, 128.9, 128.0, 127.9x2, 127.8, 127.7, 127.3x2, 127.2, 126.9, 126.7, 124.0x2, 120.6; HRMS (ESI) *m/z* [M]$^+$ calcd. for $C_{51}H_{37}NO$ = 680.2948, found = 680.2942 m/z; Elemental analysis: Anal. Calcd for $C_{51}H_{37}NO$: C, 90.1; H, 5.49; N. 2.06. Found: C, 89.86; H, 5.49; N, 2.08. R_f (2% hex/CH_2Cl_2) = 0.91

Table S1 Conditions for the reaction in solvent

no.	carboxylic acid (1) (mg; *mol*; eq)	amine (2) (mg; *mol*; eq)	solvent (ml)	coupling agent (mg; *mol*; eq)	time/ temp.	yield (mg; %)
1	20; $5.31\cdot10^{-5}$; 1.0	17.1; $5.31\cdot10^{-5}$; 1.0	DCM (4)	$SOCl_2$ 7.58; $6.37\cdot10^{-5}$; 1.2	24h/ RT	3.3 mg 9%
2	20; $5.31\cdot10^{-5}$; 1.0	17.1; $5.31\cdot10^{-5}$; 1.0	DMF (4)	EDCI 10.0; $5.31\cdot10^{-5}$; 1.0 DMAP 1.30; $1.06\cdot10^{-5}$; 0.2	24h/ RT	7.8 mg 28%
3	20; $5.31\cdot10^{-5}$; 1.0	17.1; $5.31\cdot10^{-5}$; 1.0	EtOAc (4)	EDCI 10.0; $5.31\cdot10^{-5}$; 1.0 DMAP 1.30; $1.06\cdot10^{-5}$; 0.2	24h/ RT	16.7 mg 46%
4	20; $5.31\cdot10^{-5}$; 1.0	17.1; $5.31\cdot10^{-5}$; 1.0	THF (4)	EDCI 10.0; $5.31\cdot10^{-5}$; 1.0 DMAP 1.30; $1.06\cdot10^{-5}$; 0.2	170h/ RT	16.7 mg 46%
**5**	**20; $5.31\cdot10^{-5}$; 1.0**	**17.1; $5.31\cdot10^{-5}$; 1.0**	**DCM (4)**	**EDCI 10.0; $5.31\cdot10^{-5}$; 1.0 DMAP 1.30; $1.06\cdot10^{-5}$; 0.2**	**170h/ RT**	**17.3 mg 48%**

S1.3 Synthesis of compound 3 – mechanochemistry

General method for the mechanochemical synthesis of compound 3

4-(1,2,2-Triphenylvinyl) benzoic acid (**1**), 5′-phenyl-[1,1′:3′,1″-terphenyl]-4-amine (**2**) and a coupling agent were grinded in presence of small amount of organic solvent (LAG – Liquid Assisted Grinding) at room temperature (see Table S2). Then a 1 mol·dm^{-3} hydrochloric acid solution was added to the reaction mixture, and the crude product was extracted with CH_2Cl_2 (3x20 mL). Organic layers were combined, washed with water and brine. After drying with $MgSO_4$ followed by filtration, volatiles were distilled off on a rotary evaporator. Finally, the product was purified using a column chromatography (SiO_2, 2% hex/CH_2Cl_2) to provide the target compound **3** as a yellow solid.

Table S2 Conditions for the mechanochemical synthesis

no.	carboxylic acid (1) (mg; *mol*; eq)	amine (2) (mg; *mol*; eq)	solvent (µl)	coupling agent (mg; *mol*; eq)	time/ temp.	yield (mg; %)
6	20; $5.31 \cdot 10^{-5}$; 1.0	17.1; $5.31 \cdot 10^{-5}$; 1.0	DCM (50)	k-Oxyma 9.56; $5.31 \cdot 10^{-5}$; 1	15min/ RT	grinding in hand-held mortar 0.0 mg / 0%
7	20; $5.31 \cdot 10^{-5}$; 1.0	17.1; $5.31 \cdot 10^{-5}$; 1.0	DCM (50)	EDCl 10.0; $5.31 \cdot 10^{-5}$; 1.0 NHS 6.11; $5.31 \cdot 10^{-5}$; 1	15min/ RT	grinding in hand-held mortar 1.0 mg / 3%
8	20; $5.31 \cdot 10^{-5}$; 1.0	17.1; $5.31 \cdot 10^{-5}$; 1.0	DCM (50)	CDI 8.61; $5.31 \cdot 10^{-5}$; 1.0	15 min/ RT	grinding in hand-held mortar 2.7 mg / 5%
9	20; $5.31 \cdot 10^{-5}$; 1.0	17.1; $5.31 \cdot 10^{-5}$; 1.0	DCM (50)	HBTU 20.1; $5.31 \cdot 10^{-5}$; 1.0	15 min/ RT	grinding in hand-held mortar 2.8 mg / 8%
10	20; $5.31 \cdot 10^{-5}$; 1.0	17.1; $5.31 \cdot 10^{-5}$; 1.0	DCM (50)	EDCl 10.0; $5.31 \cdot 10^{-5}$; 1.0 $FeCl_3$ 43.1; $1.59 \cdot 10^{-4}$; 3	15 min/ RT	grinding in hand-held mortar 4.2 mg / 12%
11	20; $5.31 \cdot 10^{-5}$; 1.0	17.1; $5.31 \cdot 10^{-5}$; 1.0	DCM (50)	EDCl 10.0; $6.44 \cdot 10^{-5}$; 1.2 K_3PO_4 33.8; $1.59 \cdot 10^{-4}$; 3	15 min/ RT	grinding in hand-held mortar 7.9 mg / 22%
12	20; $5.31 \cdot 10^{-5}$; 1.0	17.1; $5.31 \cdot 10^{-5}$; 1.0	-	EDC 10.0; $6.44 \cdot 10^{-5}$; 1.2	15 min/ RT	grinding in hand-held mortar 7.9 mg / 22%
13	20; $5.31 \cdot 10^{-5}$; 1.0	17.1; $5.31 \cdot 10^{-5}$; 1.0	DCM (50)	EDCl 10.0; $5.31 \cdot 10^{-5}$; 1.0 DIPEA 20.6; $1.59 \cdot 10^{-4}$; 3.0	15 min/ RT	grinding in hand-held mortar 8.4 mg / 23%
14	20; $5.31 \cdot 10^{-5}$; 1.0	17.1; $5.31 \cdot 10^{-5}$; 1.0	DCM (50)	EDCl 10.0; $5.31 \cdot 10^{-5}$; 1.0 K_2CO_3 22.0; $1.59 \cdot 10^{-4}$; 3.0	15 min/ RT	grinding in hand-held mortar 8.8 mg / 24%
15	20; $5.31 \cdot 10^{-5}$; 1.0	17.1; $5.31 \cdot 10^{-5}$; 1.0	DCM (50)	EDCl 10.0; $5.31 \cdot 10^{-5}$; 1.0 DMAP 1.50; $1.06 \cdot 10^{-5}$; 0.2	15 min/ RT	grinding in hand-held mortar 9.3 mg / 26%
16	20; $5.31 \cdot 10^{-5}$; 1.0	17.1; $5.31 \cdot 10^{-5}$; 1.0	DCM (50)	EDCl 10.0; $5.31 \cdot 10^{-5}$; 1.0 sulfo-NHS 11.5; $5.31 \cdot 10^{-5}$; 1.0	15 min/ RT	grinding in hand-held mortar 12.7 mg / 35%
17	20; $5.31 \cdot 10^{-5}$; 1.0	17.1; $5.31 \cdot 10^{-5}$; 1.0	DCM (50)	DIC 6.70; $5.31 \cdot 10^{-5}$; 1.0	15 min/ RT	grinding in hand-held mortar 13.4 mg / 37%
18	20; $5.31 \cdot 10^{-5}$; 1.0	17.1; $5.31 \cdot 10^{-5}$; 1.0	-	EDCl 10.0; $5.31 \cdot 10^{-5}$; 1.0	15 min/ RT	grinding in hand-held mortar 14.8 mg / 41%
19	20; $5.31 \cdot 10^{-5}$; 1.0	17.1; $5.31 \cdot 10^{-5}$; 1.0	DCM (50)	DCC 10.9; $5.31 \cdot 10^{-5}$; 1.0	15 min/ RT	grinding in hand-held mortar 16.5 mg / 45%

20	20; *$5.31\cdot10^{-5}$*; 1.0	17.1; *$5.31\cdot10^{-5}$*; 1.0	DCM (50)	DCC 10.9; *$5.31\cdot10^{-5}$*; 1.0	15 min/ RT	grinding in glass tube 16.2 mg / 45%
21	20; *$5.31\cdot10^{-5}$*; 1.0	17.1; *$5.31\cdot10^{-5}$*; 1.0	DCM (50)	EDCI 10.0; *$5.31\cdot10^{-5}$*; 1.0	15 min/ RT	grinding in hand-held mortar 18.9 mg / 52%
22	20; *$5.31\cdot10^{-5}$*; 1.0	17.1; *$5.31\cdot10^{-5}$*; 1.0	EtOAc (50)	EDCI 10.0; *$5.31\cdot10^{-5}$*; 1.0	15 min/ RT	grinding in hand-held mortar 18.9 mg / 52%
23	20; *$5.31\cdot10^{-5}$*; 1.0	17.1; *$5.31\cdot10^{-5}$*; 1.0	DCM (50)	EDCI 10.0; *$5.31\cdot10^{-5}$*; 1.0	5 min/ RT	grinding in hand-held mortar 19.3 mg / 53%
24	20; *$5.31\cdot10^{-5}$*; 1.0	17.1; *$5.31\cdot10^{-5}$*; 1.0	DCM (50)	EDCI 10.0; *$5.31\cdot10^{-5}$*; 1.0 NaCl 18.7; *$3.2\cdot10^{-4}$*; 6.0	15 min/ RT	grinding in hand-held mortar 19.9 mg / 55%
25	20; *$5.31\cdot10^{-5}$*; 1.0	17.1; *$5.31\cdot10^{-5}$*; 1.0	DCM (50)	EDCI 10.0; *$5.31\cdot10^{-5}$*; 1.0 SiO_2 20 mg	15 min/ RT	grinding in hand-held mortar 19.9 mg / 55%
26	20; *$5.31\cdot10^{-5}$*; 1.0	17.1; *$5.31\cdot10^{-5}$*; 1.0	DCM (50)	EDCI 10.0; *$5.31\cdot10^{-5}$*; 1.0	30 min/ RT	grinding in hand-held mortar 19.9 mg / 55%
27	20; *$5.31\cdot10^{-5}$*; 1.0	17.1; *$5.31\cdot10^{-5}$*; 1.0	EtOAc (50)	EDCI 10.0; *$5.31\cdot10^{-5}$*; 1.0	30 min/ RT	grinding in glass tube 28.4 mg / 80%
28	20; *$5.31\cdot10^{-5}$*; 1.0	17.1; *$5.31\cdot10^{-5}$*; 1.0	DCM (50)	EDCI 10.0; *$5.31\cdot10^{-5}$*; 1.0	15 min/ RT	grinding in glass tube 30.5 mg / 84%
**29**	**20; *$5.31\cdot10^{-5}$*; 1.0**	**17.1; *$5.31\cdot10^{-5}$*; 1.0**	**DCM (50)**	**EDCI 10.0; *$5.31\cdot10^{-5}$*; 1.0**	**30 min/ RT**	**grinding in glass tube 34.8 mg / 96%**

To check the repeatability of the designed grinding-induced protocol, we performed the mechanochemical synthesis of the target compound **3** under optimized mechanochemical conditions (grinding in glass vial with glass rod, reaction time: 30 minutes, 1.0 equiv. of EDCI) three times (independent runs), at the similar scales and on different days. The obtained isolated yields were consistent and equalled 93±3%. ^{1}H NMR analyses supported the isolation of pure **3** in each synthesis. The data for these experiments are presented below:

Run#1:
Synthesis date: 6.12.2022, reaction scale (mmol of amine **2**): 0.0531, mmol of the product **3** obtained: 0.0510, isolated yield of **3**: 96%

Run#2:
Synthesis date: 15.03.2024, reaction scale (mmol of amine **2**): 0.0531, mmol of the product **3** obtained: 0.0478, isolated yield of **3**: 90%

Run#3:
Synthesis date: 18.03.2024, reaction scale (mmol of amine **2**): 0.0531, mmol of the product **3** obtained: 0.0489, isolated yield of **3**: 92%

^1H NMR spectra (DMSO-d_6) of the samples of compound **3** from the above-listed mechanochemical reaction runs:

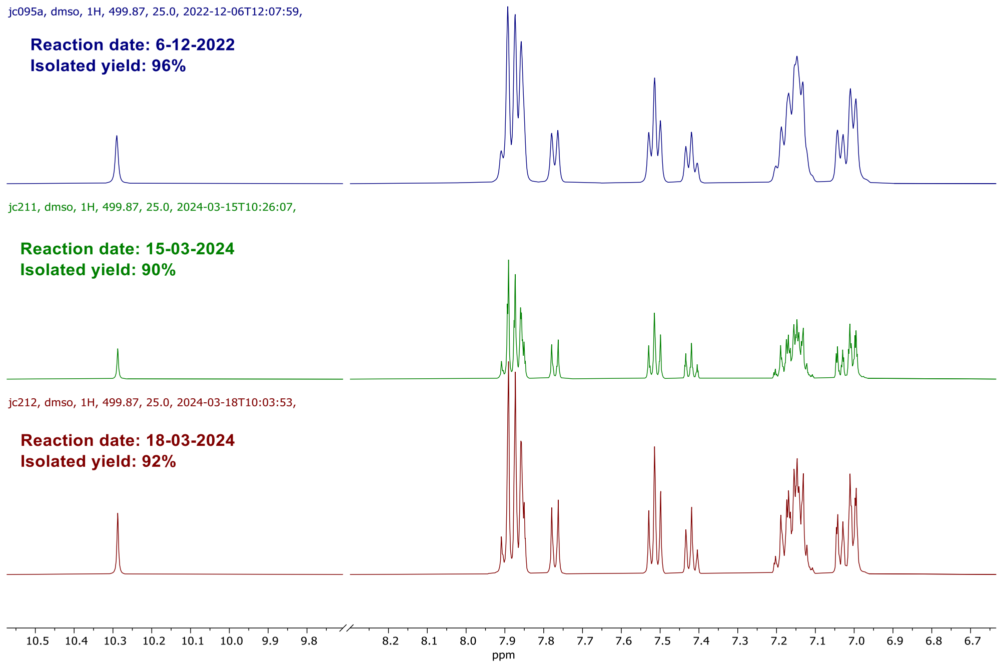

S1.4 Synthesis of compound 3 – sonochemistry

General method for the sonochemical synthesis of compound 3

In a round-bottom flask 4-(1,2,2-triphenylvinyl) benzoic acid (**1**) was placed. Then 5′-phenyl-[1,1′:3′,1″-terphenyl]-4-amine (**2**) was added, followed by coupling agent and organic solvent. The flask was then placed in an ultrasonic bath Then a 1 mol·dm^{-3} hydrochloric acid solution was added to the reaction mixture, and the crude product was extracted with CH_2Cl_2 (3x20 mL). Organic layers were combined, washed with water and brine. After drying with $MgSO_4$ followed by filtration, volatiles were distilled off on a rotary evaporator. Finally, the product was purified using a column chromatography (SiO_2, 2% hex/CH_2Cl_2) to provide the target compound **3** as yellow solid.

Table S3 Conditions for the sonochemical synthesis

no.	carboxylic acid (1) (mg; *mol*; eq)	amine (2) (mg; *mol*; eq)	solvent (ml)	coupling agent (mg; *mol*; eq)	time/ temp.	yield (mg; %)
33	20; *5.31·10⁻⁵*; 1.0	17.1; *5.31·10⁻⁵*; 1.0	EtOAc (4)	EDCl 10.0; *5.31·10⁻⁵*; 1.0	60 min/ RT	11.6 mg / 32%
34	20; *5.31·10⁻⁵*; 1.0	17.1; *5.31·10⁻⁵*; 1.0	DCM (0.2)	EDCl 10.0; *5.31·10⁻⁵*; 1.0	60 min/ RT	28.1 mg / 78%

S1.5 Green chemistry metrics

Safety considerations

Table S4 Hazards of the coupling reagents and solvents

compound	hazard statements	meaning	thermal stability
**TPE-COOH** 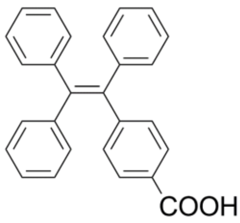	H302<br>H315<br>H319<br>H335	Harmful if swallowed<br>Causes skin irritation<br>Causes serious eye irritation<br>May cause respiratory irritation	n/a
**TPB-NH₂** 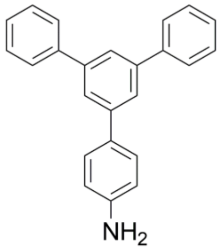	H302<br>H315<br>H319<br>H332<br>H335	Harmful if swallowed<br>Causes skin irritation<br>Causes serious eye irritation<br>Acute toxicity, inhalation<br>Specific target organ toxicity, single exposure; Respiratory system	n/a
**EDCI** 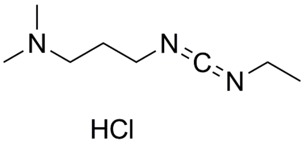	H302<br>H315<br>H319<br>H335	Harmful if swallowed<br>Causes skin irritation<br>Causes serious eye irritation<br>May cause respiratory irritation	n/a
**DMAP** 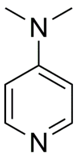	H301, H331<br>H310<br>H315<br>H318<br>H370<br>H411	Toxic if swallowed or if inhaled<br>Fatal in contact with skin<br>Causes skin irritation<br>Causes serious eye damage<br>Causes damage to organs (Nervous system)<br>Toxic to aquatic life with long lasting effects	n/a
**dichloromethane**	H315<br>H319<br>H336<br>H351	Causes skin irritation<br>Causes serious eye irritation<br>May cause drowsiness or dizziness<br>Suspected of causing cancer	n/a

First pass green metrics calculations

EDCI/ DMAP, synthesis in solution

Summary of First Pass Metrics Toolkit

Reactant (Limiting Reactant First)	Mass (g)	MW (g/mol)	Mol	Catalyst	Mass (g)	Reagent	Mass (g)	Reaction solvent	Volume (cm^3)	Density ($g \cdot ml^{-1}$)	Mass (g)	Workup chemical	Mass (g)	Workup solvent	Volume (cm^3)	Density ($g \cdot cm^{-1}$)	Mass (g)
**TPE-COOH**	**0.02**	376.45	5.31E-05			DMAP	0.0013	**DCM**	4	1.33	5.32			**n-hexane**	3	0.661	**1.983**
**TPB-NH_2**	**0.017**	321.41	5.31E-05											**DCM**	147	1.33	**195.51**
**EDCI**	**0.01**	191.17	5.31E-05														
**Total**	**0.047**	889.03					0.0013				5.32						**197.49**

**Yield**	48.0
**Selectivity**	100.0
**AE**	67.2
**RME**	71.9
**OE**	96.3
**PMI total**	5834.4
**PMI reaction**	154.4
**Reagents, catalyst**	0.047
**PMI reaction solvents**	153.0
**PMI reagents**	0.037
**PMI workup chemical**	0.0
**PMI workup solvent**	5680.0

	Mass (g)	MW	Mol
**Product**	0.03477	679.846	0.0000511
**Unreacted Limited Reactant**	0.0008		

$$RME = \frac{\text{mass of isolated product}}{\text{total mass of reactants}} \text{ x } 100$$

$$AE = \frac{\text{molecular weight of product}}{\text{total molecular weight of reactants}} \text{ x } 100$$

$$PMI = \text{mass intensity} = \frac{\text{total mass in a process or process step}}{\text{mass of product}}$$

$$OE = \frac{RME}{AE} \text{ x } 100$$

Solvents (first pass)		Tick
**Preferred solvents**	water, EtOH, *n*-BuOH, *i*-PrOH, EtOAc, *i*-PrOAc, *n*-BuOAc, anisole, sulfolane	
**Problematic solvents**	DMSO, AcOH, Acetonitrile, AcOMe, THF, heptane, toluene, MTBE, cyclohexane, chlotrobenzene, Me-THF	
**Hazardous solvents**	dioxane, TEA, DME, **DCM**, DMF, **hexane**	**+**
**Highly hazardous solvents**	Et_2O, benzene, CCl_4, chloroform, nitromethane, CS_2	

Catalyst/enzyme (First pass)		Tick
catalyst or enzyme used or reaction takes place without any catalyst/	**Green Flag**	
Use of stoichometric quantities of reagents	**Amber Flag**	**+**
use of reagents in excess	**Red Flag**	

EDCI/ DMAP, synthesis in solution

Critical Elements		
Supply Remaining	Flag Colour	Note element
5-50 years	Red Flag	
50-500 years	Amber Flag	
+500 years	Green Flag	+

Energy		Tick
Reaction run between 0 to 70°C	Green Flag	+
Reaction run between -20 to 0 or 70 to 140°C	Amber Flag	
Reaction run between below -20 or above 140°C	Red Flag	

Batch/Flow		Tick
Flow	Gren Flag	
Batch	Amber Flag	+

		Tick
Reaction run at reflux	Red Flag	
Reaction run 5°C or below the solvent boiling point	Green Flag	+

Workup		Tick
quenching<br>filtration<br>centrifugation<br>crystalisation<br>low temperature distillation/ evaporation/ sublimation	Green Flag	
solvent exchange, quenching into aqueous solvent	Amber Flag	
chromatography<br>high temperature<br>multiple crystalisation	Red Flag	+

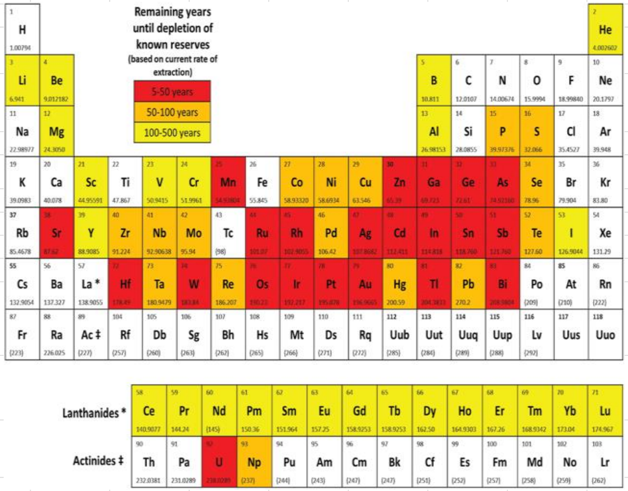

Health and Safety

	Red Flag	Amber Flag	Green Flag	List substances nad H-codes	List substances nad H-codes	List substances nad H-codes
Highly Explosive	H200, H201, H202, H203	H205, H220, H224	If no red or amber flagged H codes present then green flag			TPE-COOH
Explosive thermal runaway	H230, H240, H250	H241				TPB-NH_2
Toxic	H300, H310, H330	H301, H311, H331		DMAP	DMAP	
Long Term Toxicity	H340, H350, H360, H370, H372	H341, H351, H361, H371, H373				DCM
Environmental Implications	H400, H410, H411, H420	H401, H412				

Use of chemicals of environmental concern		List of substances
Chemical identified as Substances of Very High Concern by ChemSec which are utilised	Red Flag	

EDCI, mechanochemistry

Summary of First Pass Metrics Toolkit

Reactant (Limiting Reactant First)	Mass (g)	MW (g/mol)	Mol	Catalyst	Mass (g)	Reagent	Mass (g)	Reaction solvent	Volume (cm^3)	Density ($g·ml^{-1}$)	Mass (g)	Workup chemical	Mass (g)	Workup solvent	Volume (cm^3)	Density ($g·cm^{-1}$)	Mass (g)
**TPE-COOH**	**0.02**	376.45	5.30E-05					**DCM**	0.05	1.33	0.0665			**n-hexane**	3	0.661	**1.983**
**TPB-NH_2**	**0.017**	321.41	5.30E-05											**DCM**	147	1.33	**195.51**
**EDCI**	**0.01**	191.17	5.30E-05														
**Total**	**0.047**	889.03									0.07						**197.49**

**Yield**	96.0
**Selectivity**	100.0
**AE**	76.4
**RME**	73.4
**OE**	96.1
**PMI total**	5683.2
**PMI reaction**	3.3
**Reagents, catalyst**	0.000
**PMI reaction solvents**	1.9
**PMI reagents**	0.0
**PMI workup chemical**	0.0
**PMI workup solvent**	5680.0

	Mass (g)	MW	Mol
**Product**	0.03477	679.846	0.0000511
**Unreacted Limited Reactant**	0.0008		

$$\text{RME} = \frac{\text{mass of isolated product}}{\text{total mass of reactants}} \text{ x } 100$$

$$\text{AE} = \frac{\text{molecular weight of product}}{\text{total molecular weight of reactants}} \text{ x } 100$$

$$\text{PMI} = \text{mass intensity} = \frac{\text{total mass in a process or process step}}{\text{mass of product}}$$

$$\text{OE} = \frac{\text{RME}}{\text{AE}} \text{ x } 100$$

Solvents (first pass)		Tick
**Preferred solvents**	water, EtOH, *n*-BuOH, *i*-PrOH, EtOAc, *i*-PrOAc, *n*-BuOAc, anisole, sulfolane	
**Problematic solvents**	DMSO, AcOH, Acetonitrile, AcOMe, THF, heptane, toluene, MTBE, cyclohexane, chlotrobenzene, Me-THF	
**Hazardous solvents**	dioxane, TEA, DME, **DCM**, DMF, **hexane**	**+**
**Highly hazardous solvents**	Et_2O, benzene, CCl_4, chloroform, nitromethane, CS_2	

Catalyst/enzyme (First pass)		Tick
catalyst or enzyme used or reaction takes place without any catalyst/ reagent	Green Flag	
Use of stoichometric quantities of reagents	Amber Flag	**+**
Use of reagents in excess	Red Flag	

EDCI, mechanochemistry

Critical Elements

Supply Remaining	Flag Colour	Note element
5-50 years	Red Flag	
50-500 years	Amber Flag	
+500 years	Green Flag	+

		Tick
Reaction run at reflux	Red Flag	
Reaction run 5°C or below the solvent boiling point	Green Flag	+

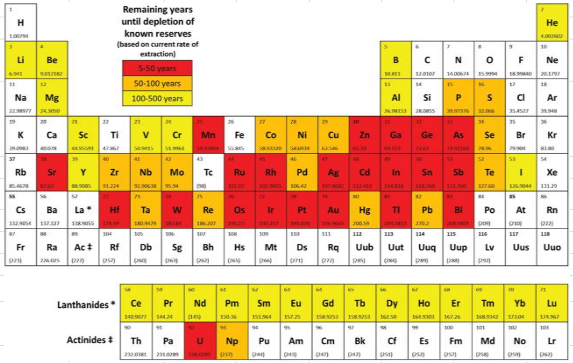

Energy

		Tick
Reaction run between 0 to 70°C	Green Flag	+
Reaction run between -20 to 0 or 70 to 140°C	Amber Flag	
Reaction run between below -20 or above 140°C	Red Flag	

Workup

Workup		Tick
quenching	Green Flag	
filtration		
centrifugation		
crystalisation		
low temperature distillation/ evaporation/ sublimation		
solvent exchange, quenching into aqueous solvent	Amber Flag	
chromatography	Red Flag	+
high temperature		
multiple crystalisation		

Batch/Flow

		Tick
Flow	Gren Flag	
Batch	Amber Flag	+

Health and Safety

	Red Flag	Amber Flag	Green Flag	List substances nad H-codes	List substances nad H-codes	List substances nad H-codes
Highly Explosive	H200, H201, H202, H203	H205, H220, H224	If no red or amber flagged H codes present then green flag			TPE-COOH
Explosive thermal runaway	H230, H240, H250	H241				TPB-NH_2
Toxic	H300, H310, H330	H301, H311, H331				EDCI
Long Term Toxicity	H340, H350, H360, H370, H372	H341, H351, H361, H371, H373				DCM
Environmental Implications	H400, H410, H411, H420	H401, H412				

Use of chemicals of environmental concern		List of substances
Chemical identified as Substances of Very High Concern by ChemSec which are utilised	Red Flag	

S1.6 Characterisation of aggregation induced emission effect

The characterisation of aggregation induced emission behavior of compound **3** was performed employing measurements of the emission spectra. The experiments were performed in the DMSO/H_2O solvent mixture. Stock solution of **3** ($2 \cdot 10^{-3}$ M) in DMSO was diluted with proper volume of pure DMSO followed by addition of H_2O to reach given vol% of H_2O in the sample.

S1.7 Anion binding experiments

S1.7.1 ^{1}H NMR spectroscopy

The binding experiments between compound **3** (receptor) and anions (Br^-, AMP and ADP) were performed employing the ^{1}H NMR titration experiments. Tetrabutylammonium bromide ($[N(C_4H_9)_4]^+$) was used in these experiments. The experiments were performed in DMSO-d_6 containing TMS (tetramethylsilane, 0.03% *vol*) as follows. To a stock solution of **3** ($7.5 \cdot 10^{-3}$ M) in DMSO-d_6 a stock solution of analyte ($7.5 \cdot 10^{-3}$ M) in DMSO-d_6 was added, followed by addition of DMSO-d_6 to reach given concentration of analyte in the sample (in case of AMP and ADP stock solution of **3** and stock solution of analyte were mixed in such a way that the sum of receptor (**3**) and analyte concentrations in the sample were on the constant level with varying molar fractions). Final volume of the samples was 1 mL.

S1.7.2 Spectrofluorimetry

The anion binding experiments between compound **3** (receptor) and anions (analytes; Br^-, I^-, HSO_4^-, BF_4^-, $H_2PO_4^-$, ClO_4^-, CN^-, AMP, ADP, ATP, NADP and FAD) were performed employing the emission spectra titration experiments. In all cases, tetrabutylammonium ($[N(C_4H_9)_4]^+$) salts of anions were used. The experiments were performed in the DMSO/H_2O = 1:1 *v*/*v* system as follows. Stock solution of **3** ($2 \cdot 10^{-3}$ M) in DMSO was diluted with adequate volume of pure DMSO (to reach volume of 1 mL), followed by addition of H_2O solution containing given anion (final concentration of anion was between $5 \cdot 10^{-6}$ M and $2 \cdot 10^{-4}$ M)

S1.9 Estimation of fluorescence quantum yield

The measurements were performed at room temperature according to the published procedures.[1,2] Fluorescence quantum yields (Φ_F) were determined by comparison with quinine sulfate (QS) in 0.5M H_2SO_4 ($\Phi_{F,ref}$ = 0.55 [3]) as the standard. The measurements were performed with diluted solutions (absorbance for the highest wavelength A < 0.1 a.u.). The selected excitation

wavelengths (λ_{ex}) were as follows: C_{QS} = 2·10^{-6} M; $C_{\mathbf{3}}$ = 2·10^{-6} M, λ_{ex} = 351 nm; $C_{\mathbf{3agg.}}$ = 2·10^{-6} M, λ_{ex} = 343 nm.

The following formula was used for the calculation of Φ_F:

$$\Phi_F = \Phi_{F,ref} \cdot \frac{F_{sample}}{F_{reference}} \cdot \frac{1-10^{-A_{ref}}}{1-10^{-A_{sample}}} \cdot \frac{n^2_{sample}}{n^2_{reference}}$$

where $\Phi_{F,ref}$ is the quantum yield for QS (0.55[1]), *F* is the integrated area under the fluorescence spectra, *A* is the absorbance at the excitation wavelength, *n* is the refractive index of the solvent (1.346 for 0.5M H_2SO_4, 1.4772 for DMSO, *n* for the aggregates solution (DMSO/H_2O = 1:1 *v*/*v*) was taken as weighted arithmetic mean with weights equal to vol% of H_2O (*n* = 1.3329) and DMSO in the mixture). The calculated Φ_F for **3** and aggregates of **3** , were 0.0042 and 0.2437, respectively.

S2. NMR spectra

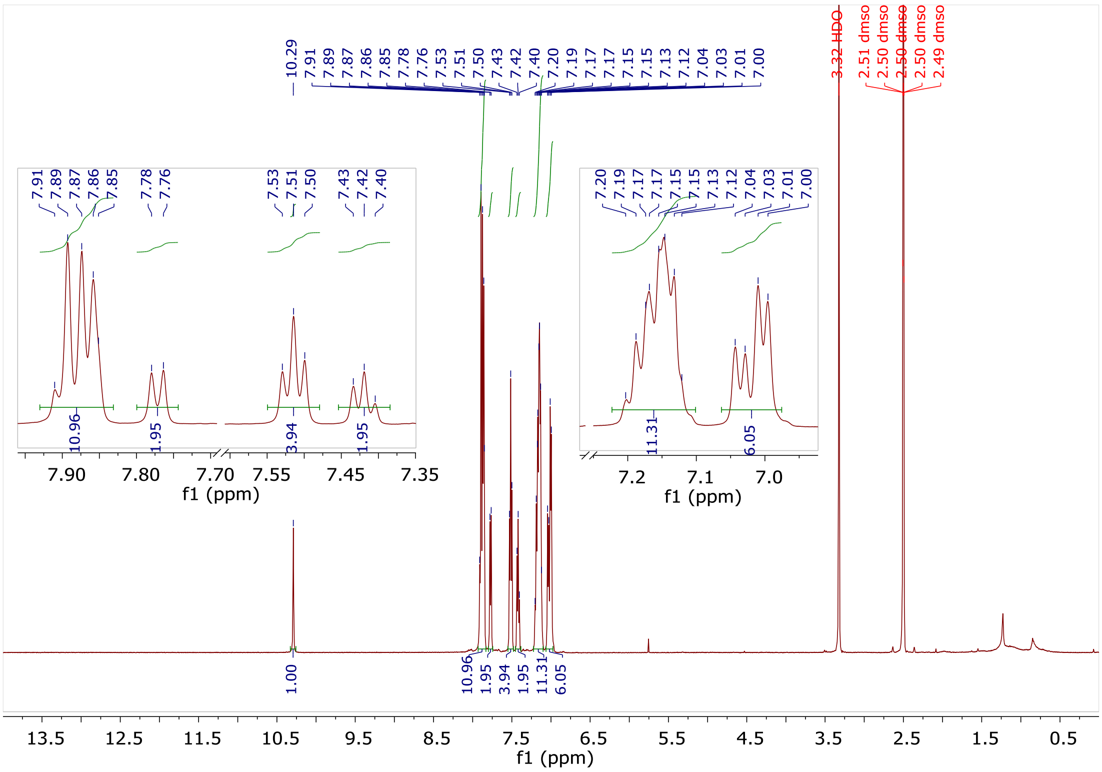

Figure S 1 ^{1}H NMR (500 MHz, DMSO-d$_6$) spectrum of **3**

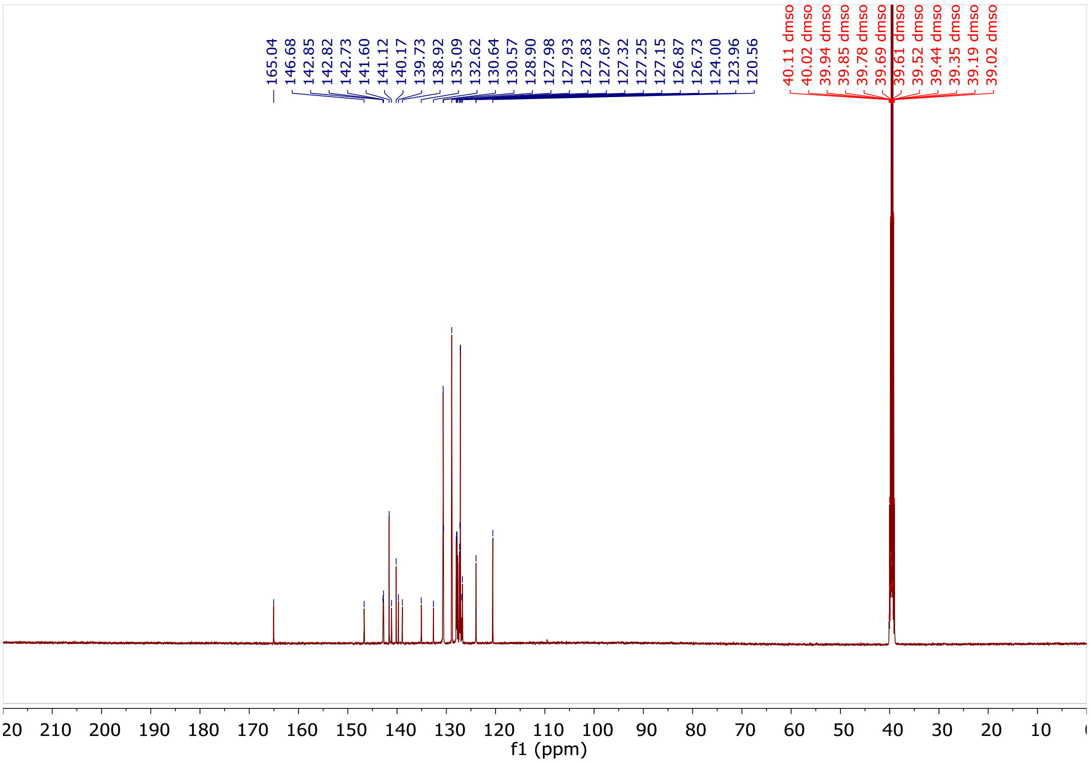

Figure S 2 {^{1}H}^{13}C NMR (125 MHz, DMSO-*d*$_6$) spectrum of **3**

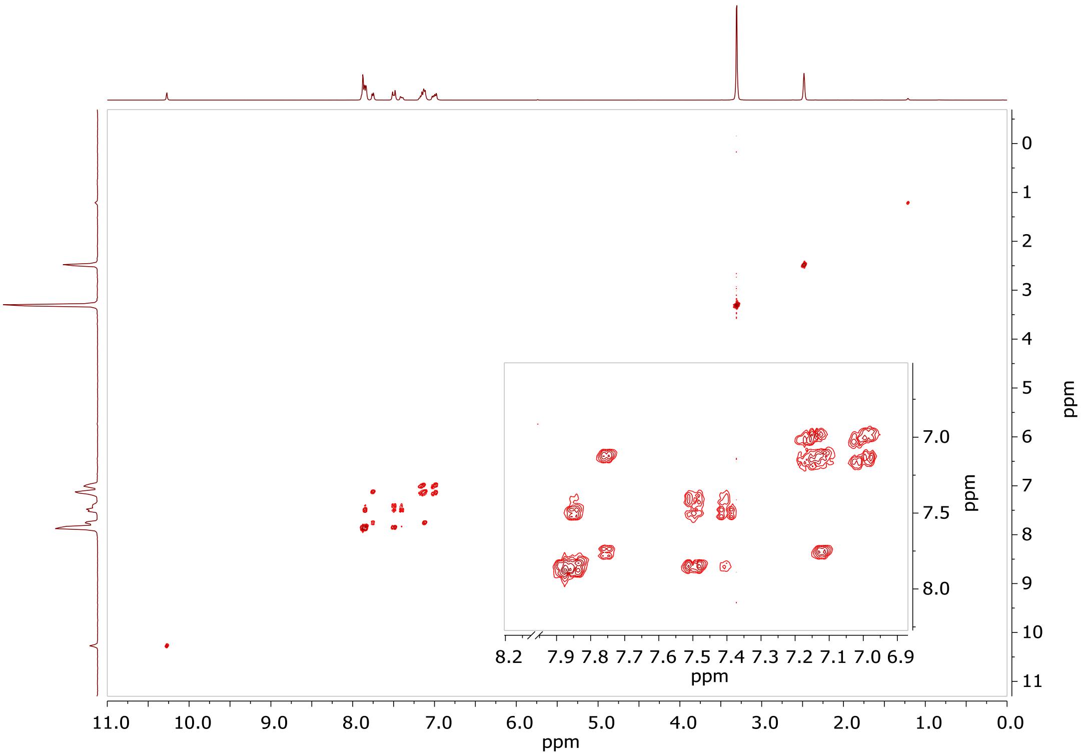

Figure S 3 ^{1}H-^{1}H COSY NMR (500 MHz, DMSO-d6) spectrum of **3**

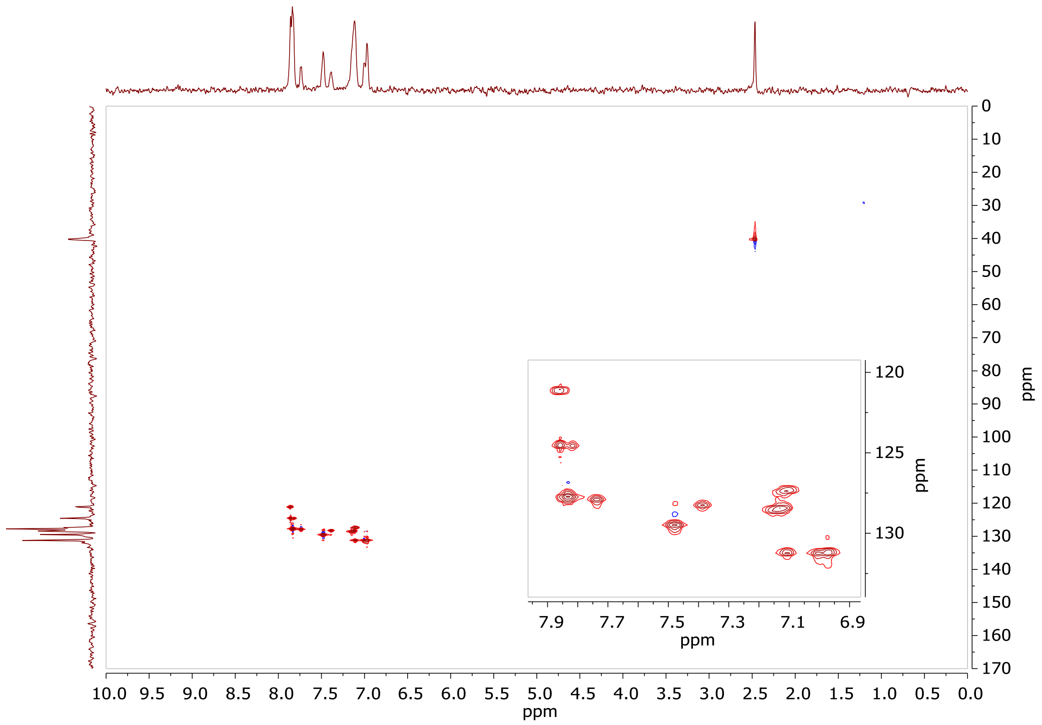

Figure S 4 ^{1}H-^{13}C HSQC NMR (DMSO-d6) spectrum of **3**

S3 HRMS spectrum

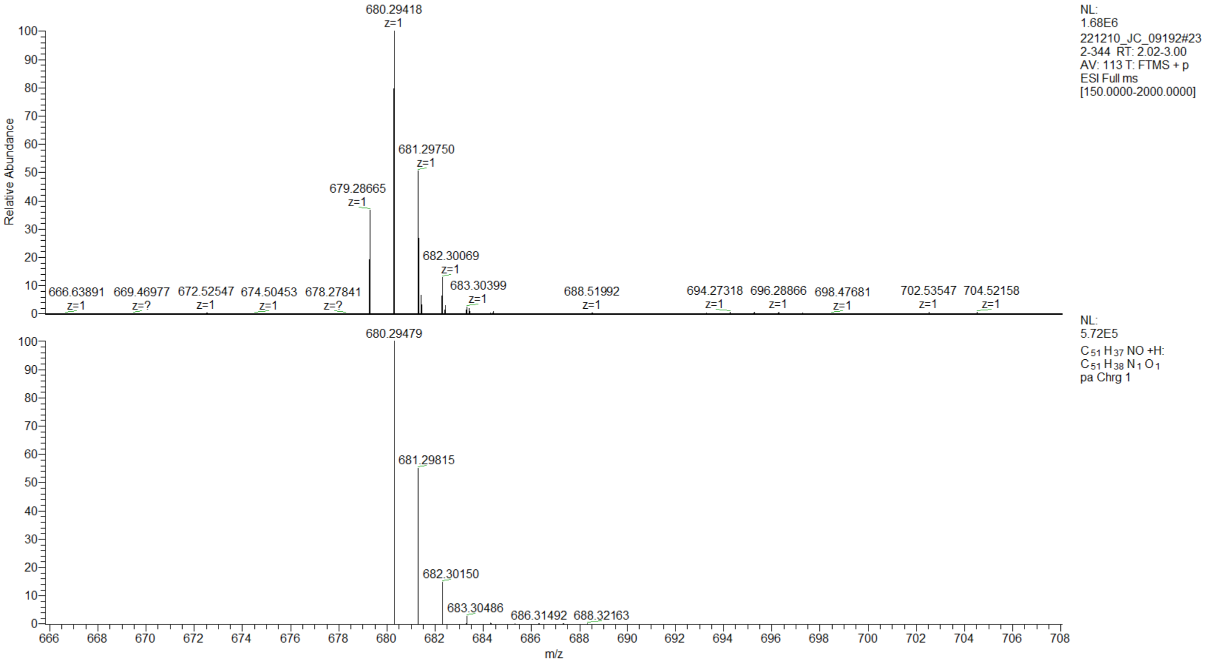

Figure S 5 ESI-HRMS (TOF) spectrum of **3**

S4. Absorption and emission spectra

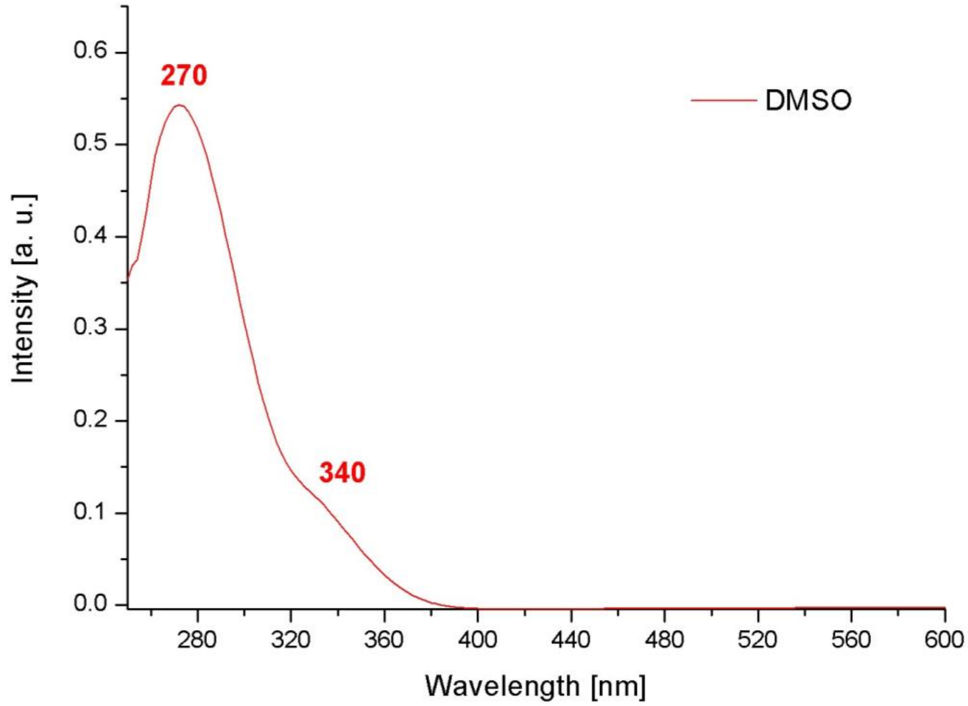

Figure S 6 UV-vis spectrum of compound **3** (DMSO, $C_3 = 2 \cdot 10^{-5}$ M)

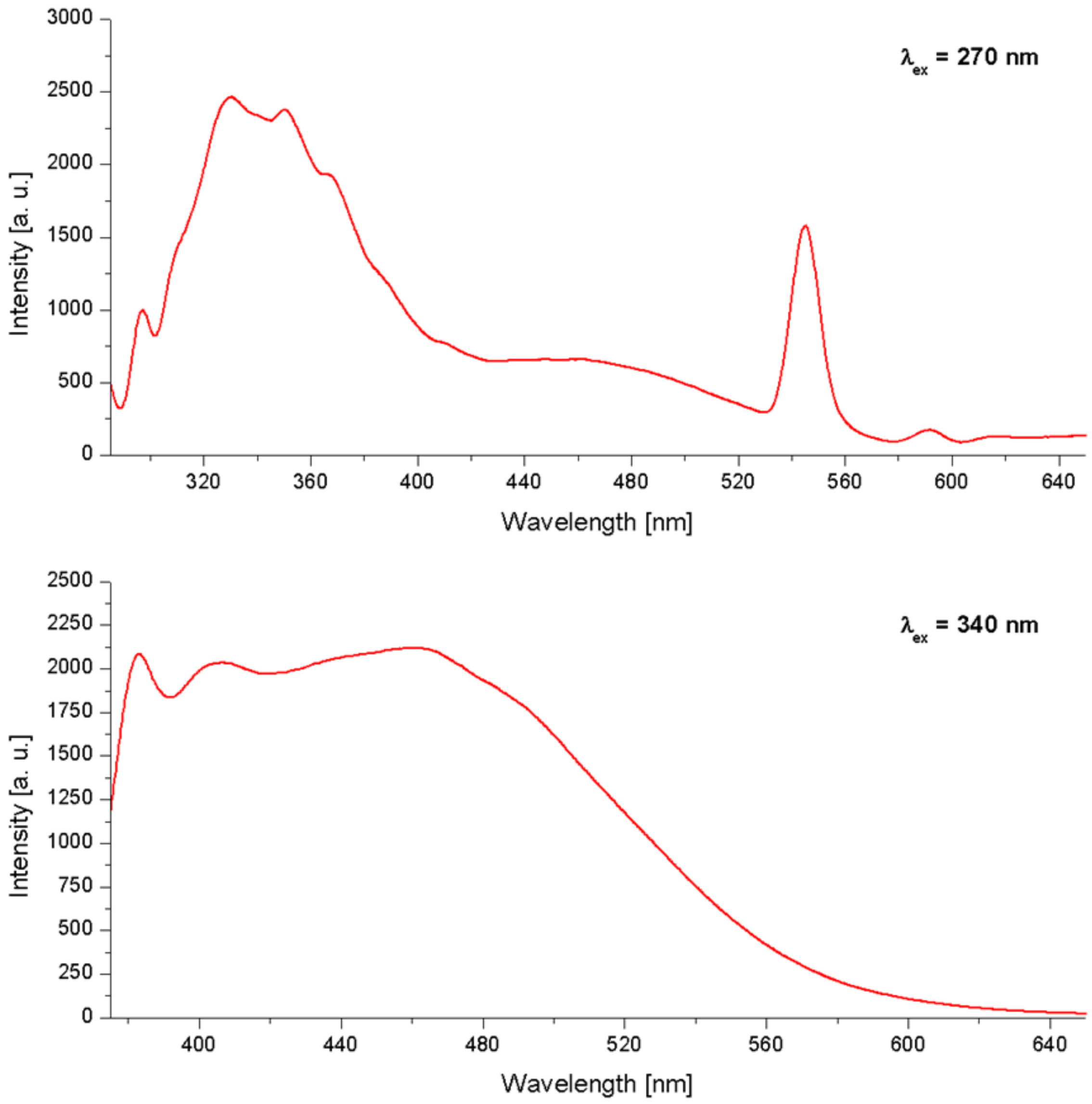

Figure S 7 Emission spectra of **3** (DMSO, $C_3 = 2 \cdot 10^{-5}$ M, λ_{ex} = 270 nm (top), λ_{ex} = 340 nm (bottom))

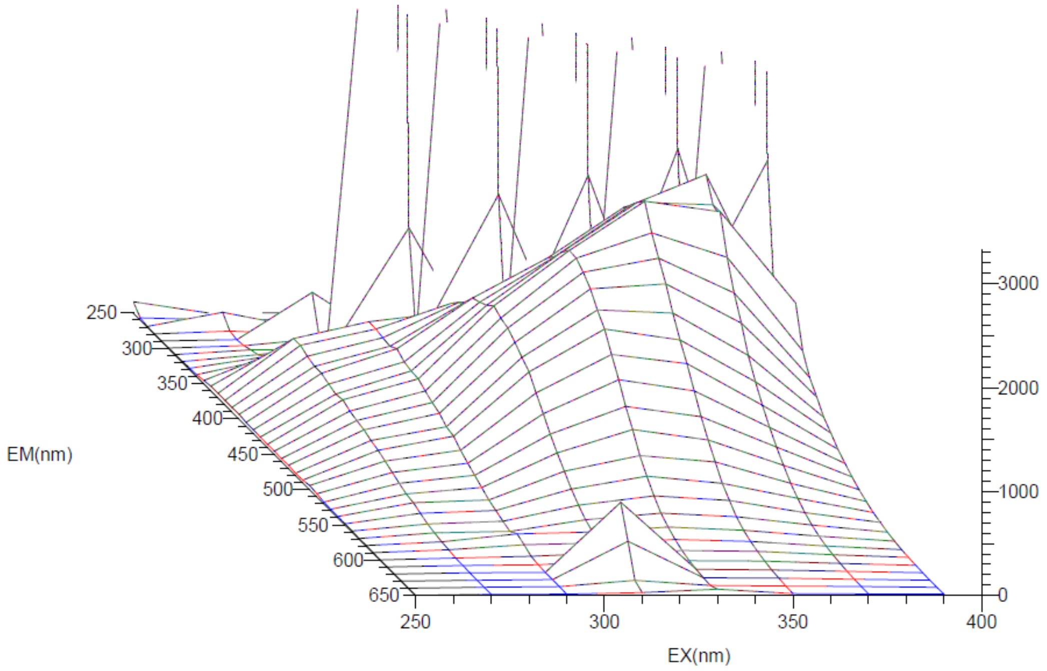

Figure S 8 3D emission spectrum of **3** (DMSO, $C_3 = 2 \cdot 10^{-5}$ M)

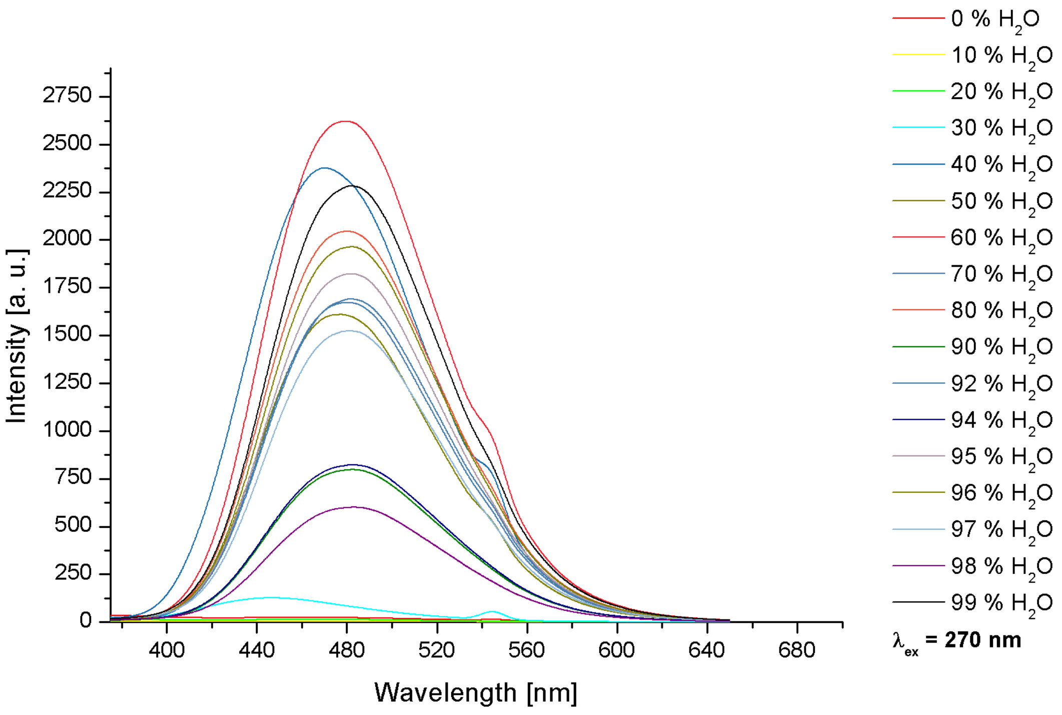

Figure S 9 Emission spectra (λ_{ex} = 270 nm) of compound **3** in DMSO/H_2O system containing different vol% of water in the sample ($C_3 = 2 \cdot 10^{-5}$ M,)

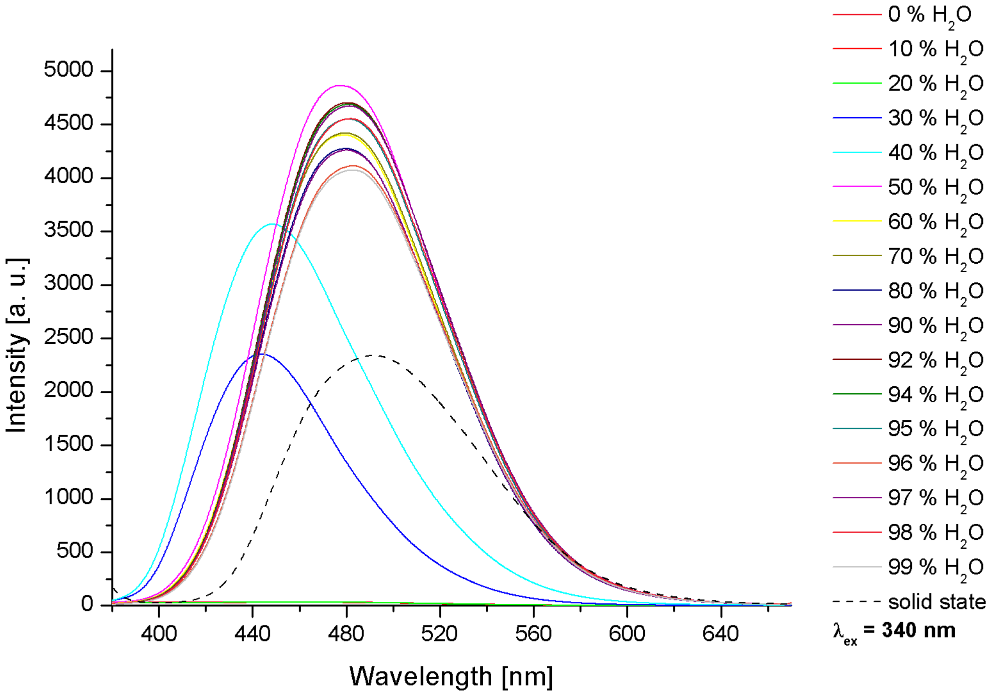

Figure S 10 Emission spectra (λ_{ex} = 340 nm) of compound **3** in DMSO/H_2O system containing different vol% of water in the sample ($C_3 = 2{\cdot}10^{-5}$ M)

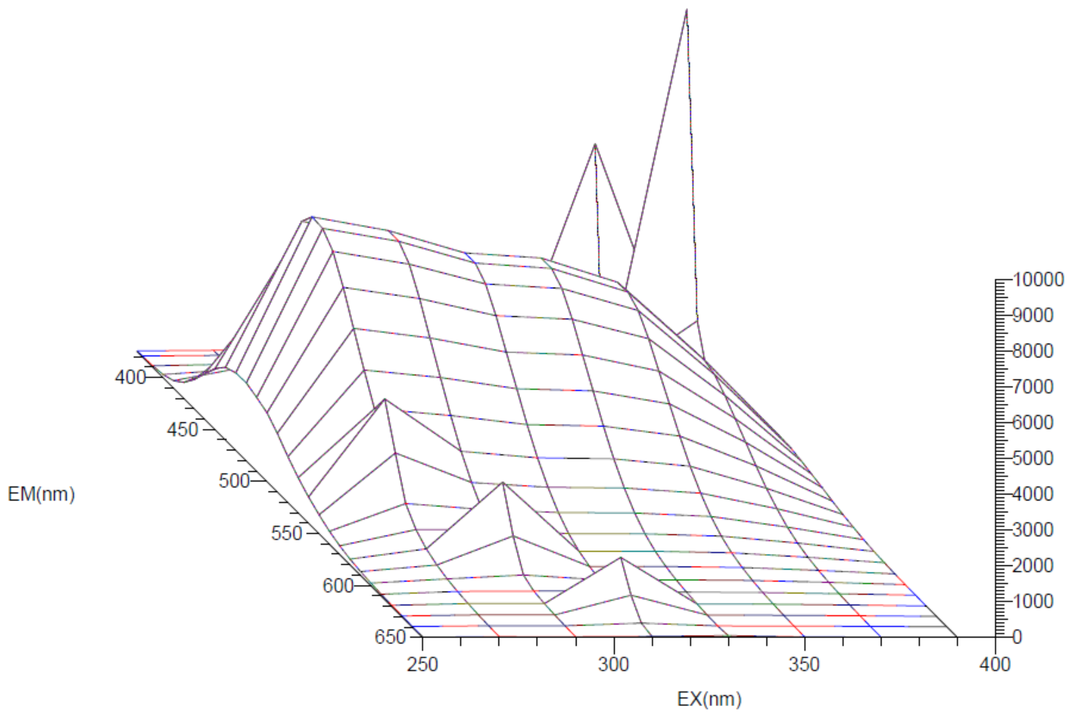

Figure S 11 3D emission spectra of **3** (DMSO/H_2O = 1:1 *v/v*, $C_3 = 2{\cdot}10^{-5}$ M)

S5. Anions binding experiments

For anions for which a decrease in emission intensity was observed (I^-, HSO_4^-, BF_4^-, $H_2PO_4^-$, CN^-, AMP, ADP, ATP, NADP, FAD) the Stern-Volmer constant values (K_{sv}) were estimated using the Stern-Volmer method, given by the equation:

$$\frac{I_0}{I} = 1 + K_{SV}$$

where I_0 and I are the fluorescence intensities of **3** in the absence and presence of given anion, respectively. K_{SV} were taken as slope of $1/C(A-)$ vs. $1/\Delta I$ linear plots.

The limit of detection (LOD) values were estimated from the plot: of $(I-I_{min})/(I_{max}-I_{min})$ vs Log ([A^-]).

For ClO_4^- where an increase in emission intensity was observed the apparent binding constant (K_{app}) values were estimated using the Benesi-Hildebrand [4,5] method, given by the equation:

$$\frac{1}{I - I_0} = \frac{1}{a} + \frac{1}{a \cdot K_{app} \cdot C(\mathrm{A}^-)}$$

where I_0 and I are the fluorescence intensities of **3** in the absence and presence of given anion, respectively, *a* is a constant, and $C(A^-)$ is the concentration of given anion in solution. K_{app} were determined as a ratio of intercept-to-slope of $1/(I - I_0)$ vs. $1/C(A^-)$ linear plots.

The data (for the estimation of K_{app} for the studied systems were collected from emission maxima (λ_{em}) = 496 nm (λ_{ex} = 270 nm).

The limit of detection (LOD) values were estimated by the equation:

LOD = 3S/b

Where S is standard error of intercept, and b is slope of regression line

The stoichiometry of the complexes formed was estimated using Job's plot method, from the plot: $(1- x)\cdot(\delta- \delta_0)$ vs x. The *x* stands from the mole fraction of nucleotide. The expected stoichiometry is indicated by the maximum on the plot.

All the spectra and plots are presented below.

S5.1 ^{1}H NMR spectroscopy

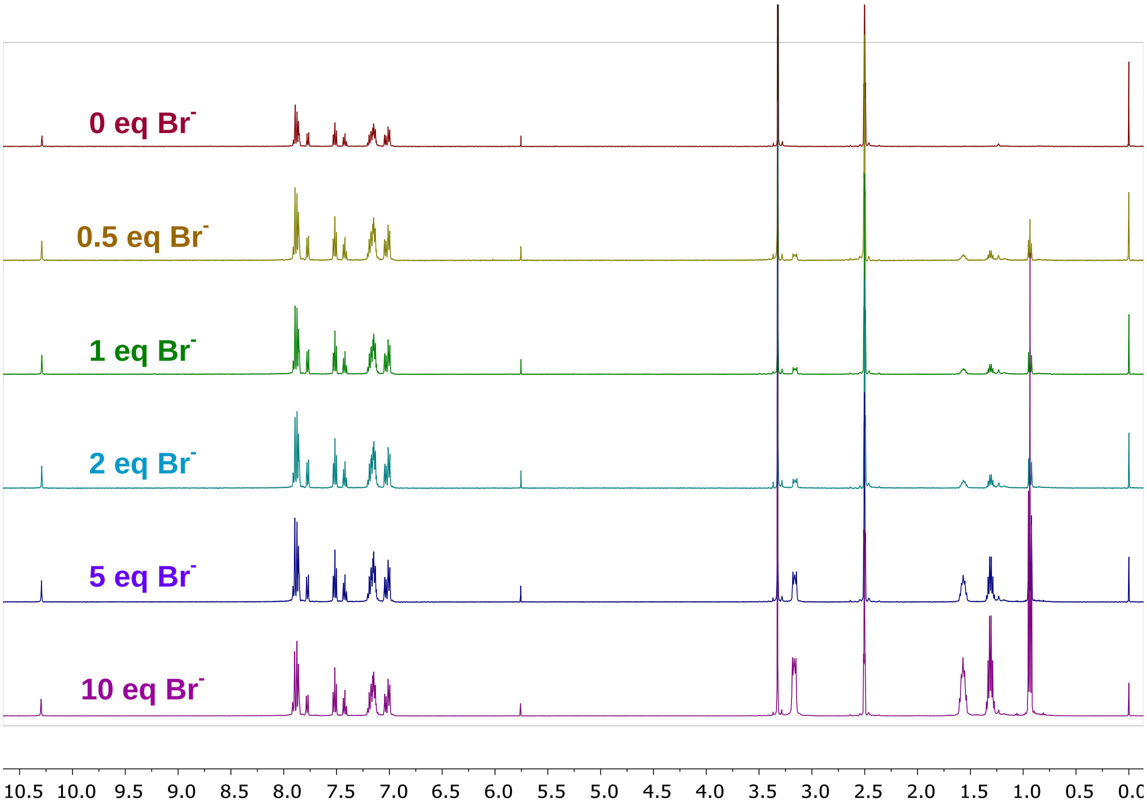

Figure S 12 ^{1}H NMR (500 MHz, DMSO-d$_6$) spectrum of **3** in presence of various molar equivalents of Br^-

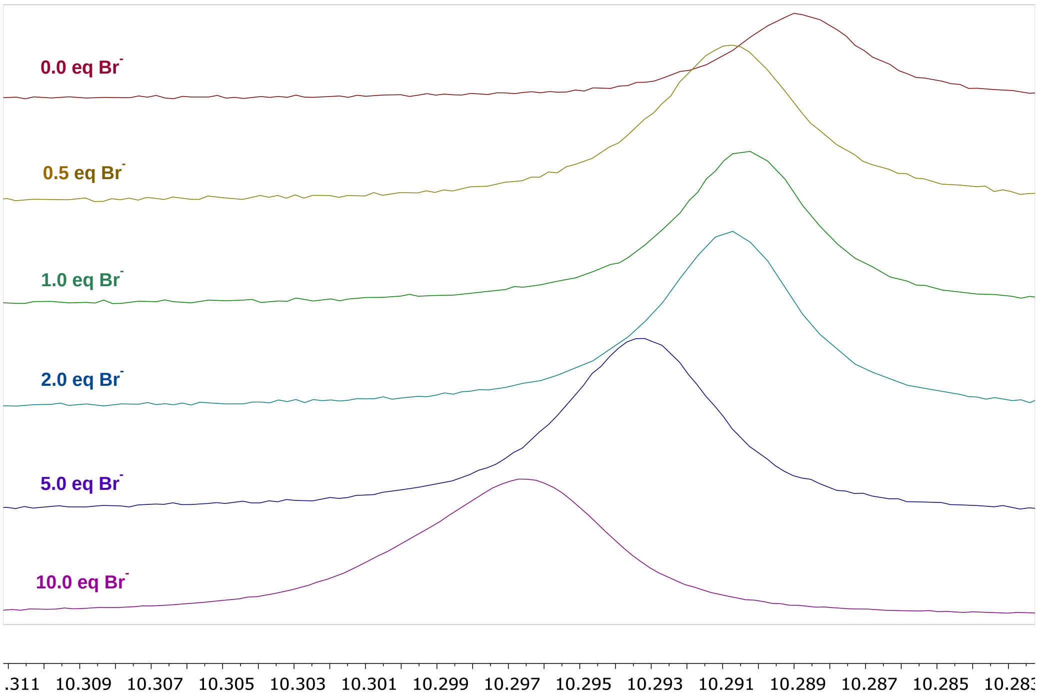

Figure S 13 Inset of the ^{1}H NMR (500 MHz, DMSO-d6) spectrum of **3** in presence of various molar equivalents of Br^-

Signals on the ^{1}H NMR spectra were assigned according to the literature data[6].

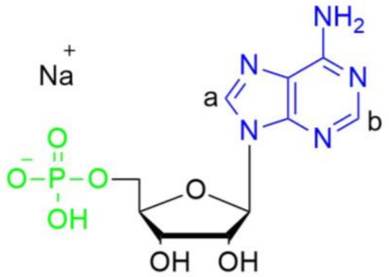

Figure S 14 Chemical formula of adenosine monophosphate (**AMP**) with the marked protons (a, b) for which the shifts of signals were observed in the ^{1}H NMR spectrum

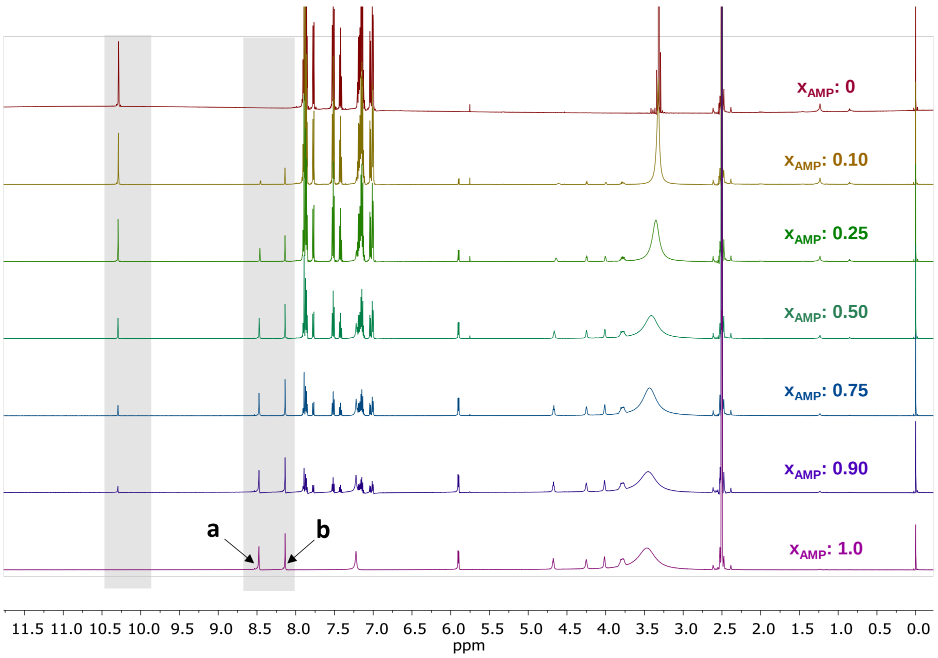

Figure S 15 ^{1}H NMR (500 MHz, DMSO-d_6) spectrum of **3** in presence of various molar equivalents of **AMP** (grey colour indicates signals that are shifted)

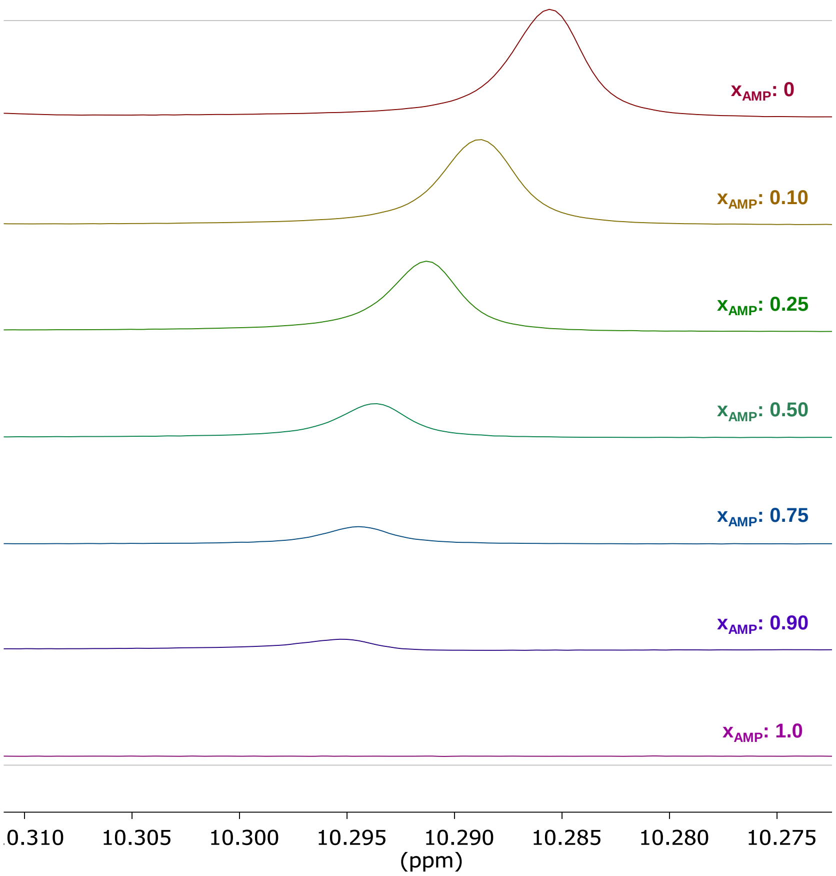

Figure S 16 Insets of the ^{1}H NMR (500 MHz, DMSO-d_6) spectrum of **3** in presence of various molar equivalents of **AMP**

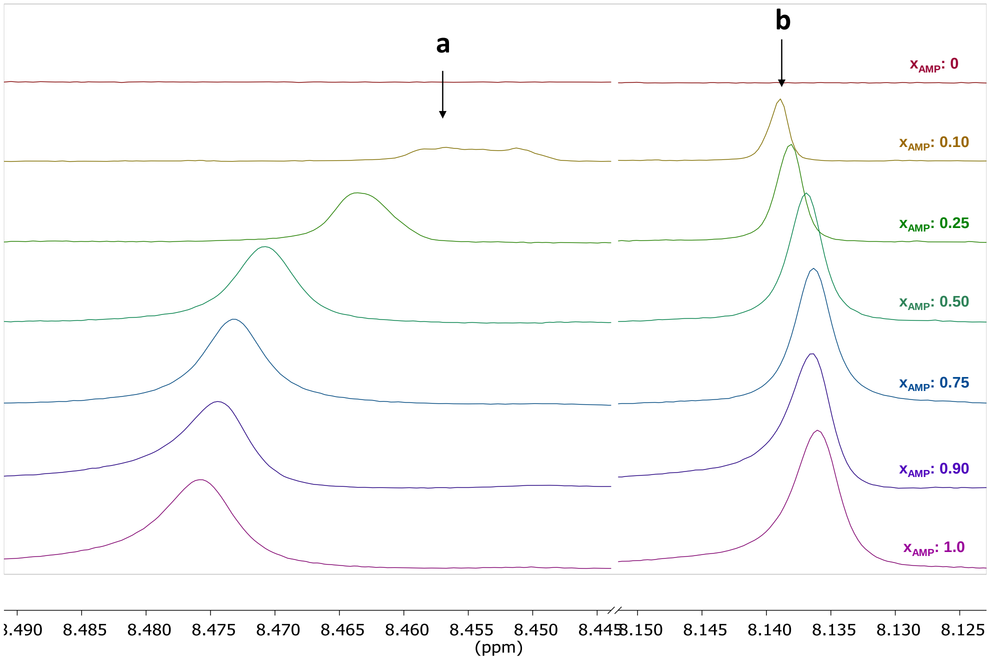

Figure S 17 Insets of the ^{1}H NMR (500 MHz, DMSO-d_6) spectrum of **3** in presence of various molar equivalents of **AMP** (amplification of the shifted proton signals of the nucleobase of the nucleotide)

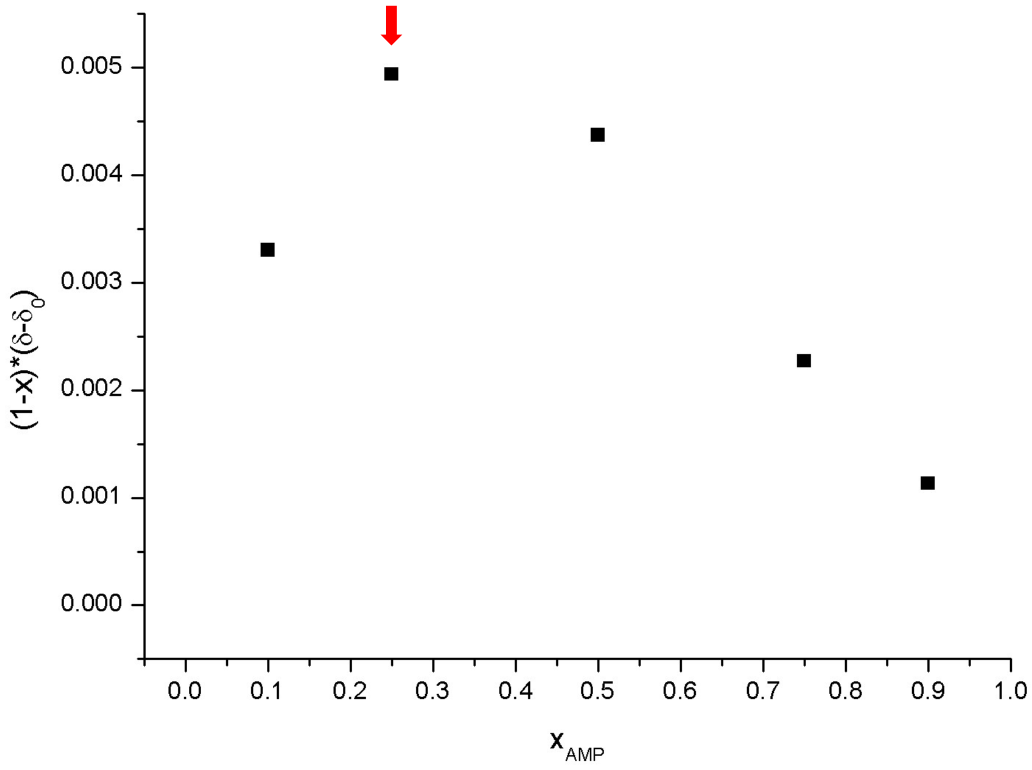

Figure S 18 Job's plot regarding the interactions between **3** and **AMP** (the red arrow indicates the estimated stoichiometry of the complex formed)

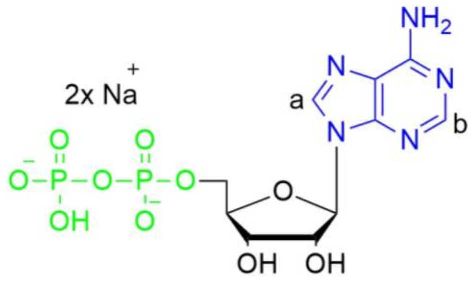

Figure S 19 Chemical formula of adenosine diphosphate (**ADP**) with the marked protons (a, b), whose signals are shifted in the ^{1}H NMR spectrum

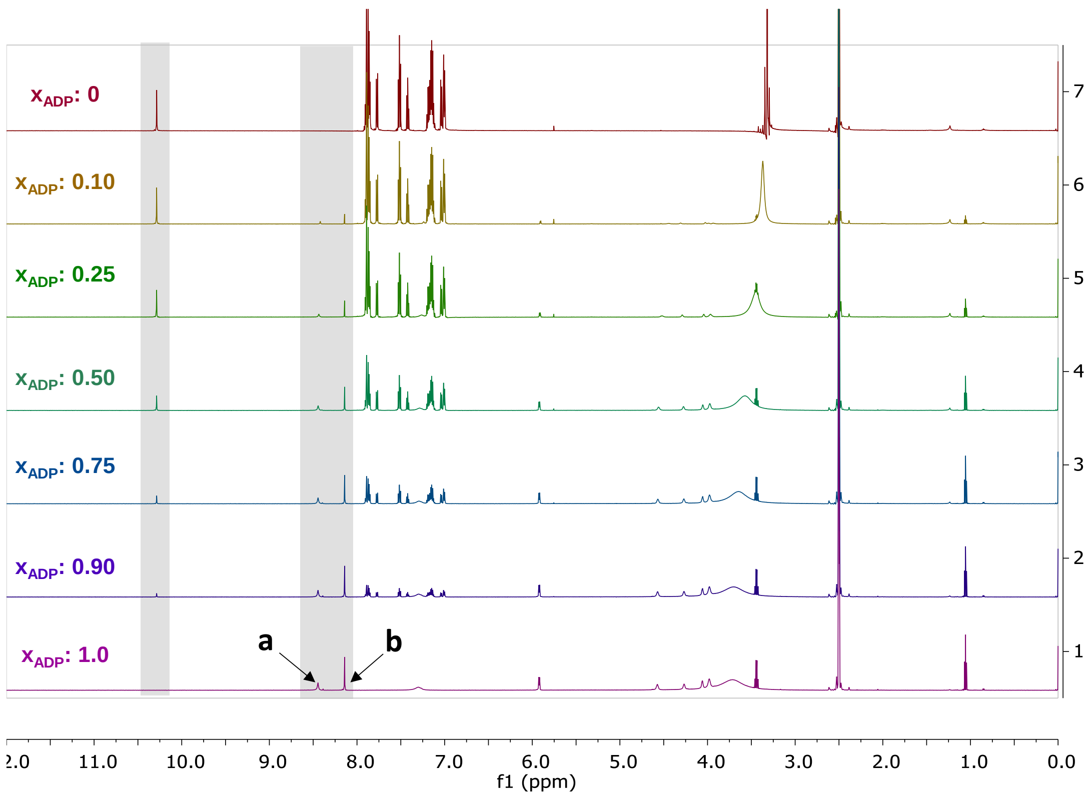

Figure S 20 ^{1}H NMR (500 MHz, DMSO-d_6) spectrum of **3** in presence of various molar equivalents of **ADP** (grey colour indicates signals that are shifted)

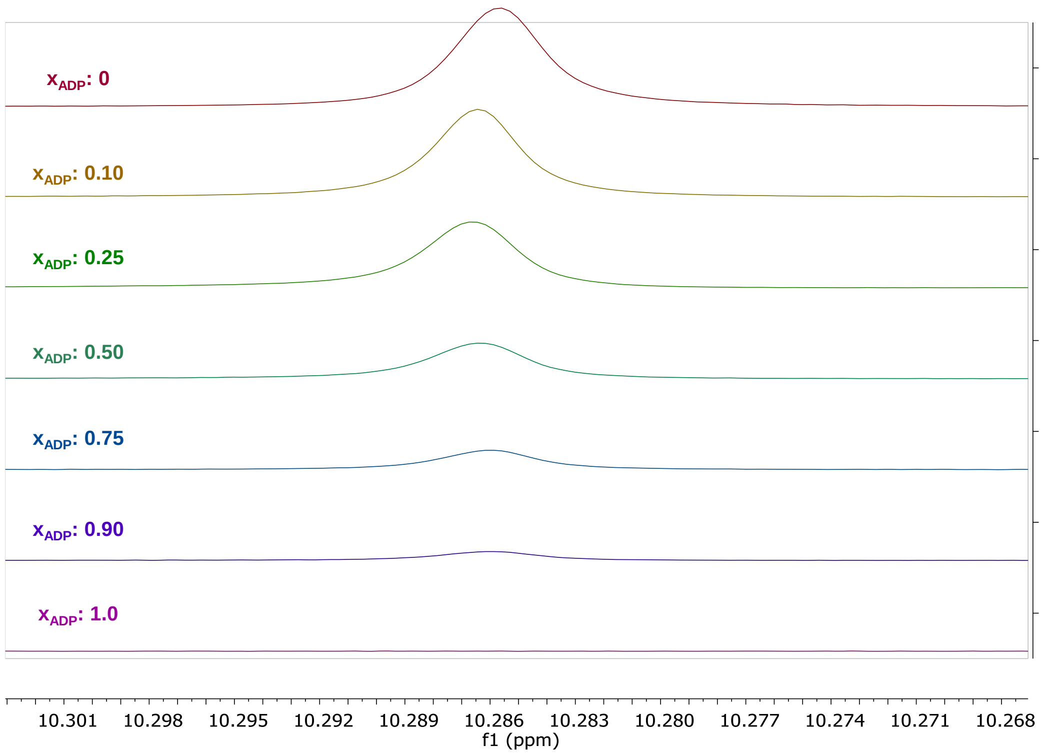

Figure S 21 Insets of the ^{1}H NMR (500 MHz, DMSO-d_6) spectrum of **3** in presence of various molar equivalents of **ADP** (amplification of the shifted proton signals of the amide group)

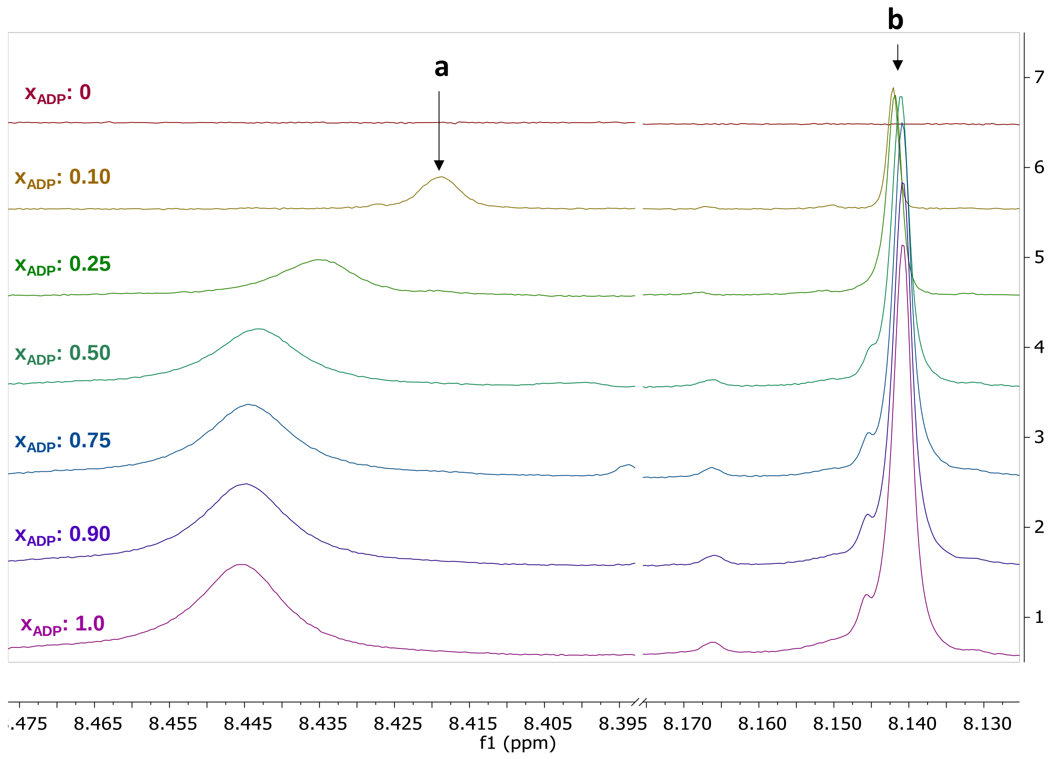

Figure S 22 Insets of the ^{1}H NMR (500 MHz, DMSO-*d*6) spectrum of **3** in presence of various molar equivalents of **ADP** (amplification of the shifted proton signals of the nucleobase of the nucleotide)

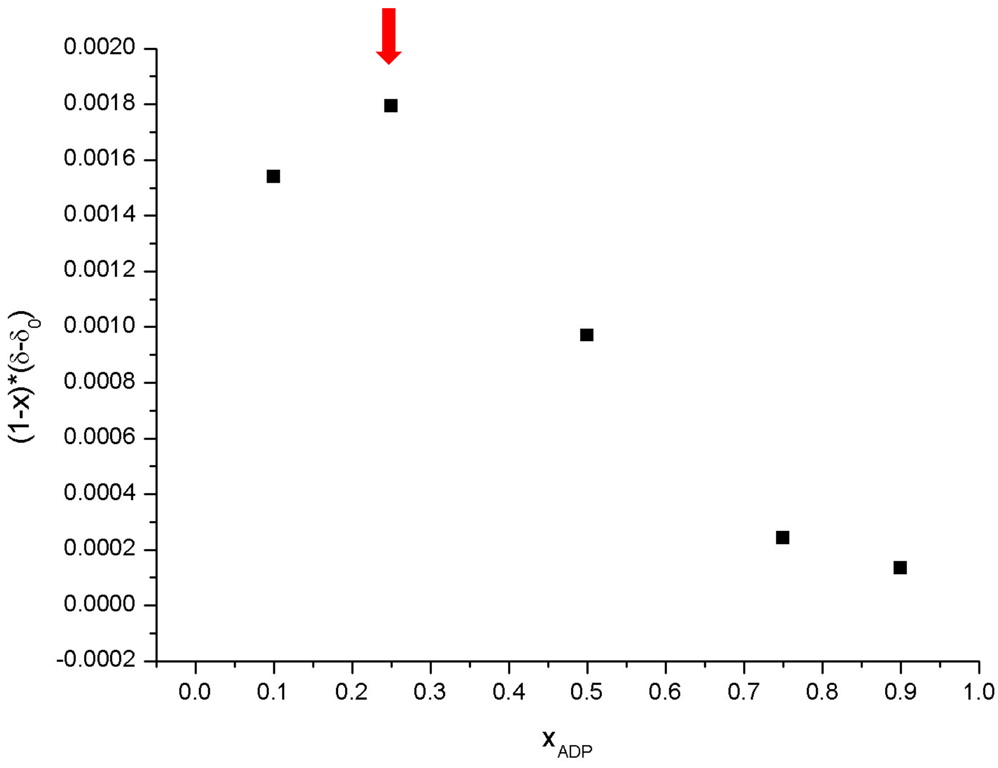

Figure S 23 Job's plot regarding the interactions between **3** and **ADP** (the red arrow indicates the estimated stoichiometry of the complex formed)

S5.2 Emission spectra

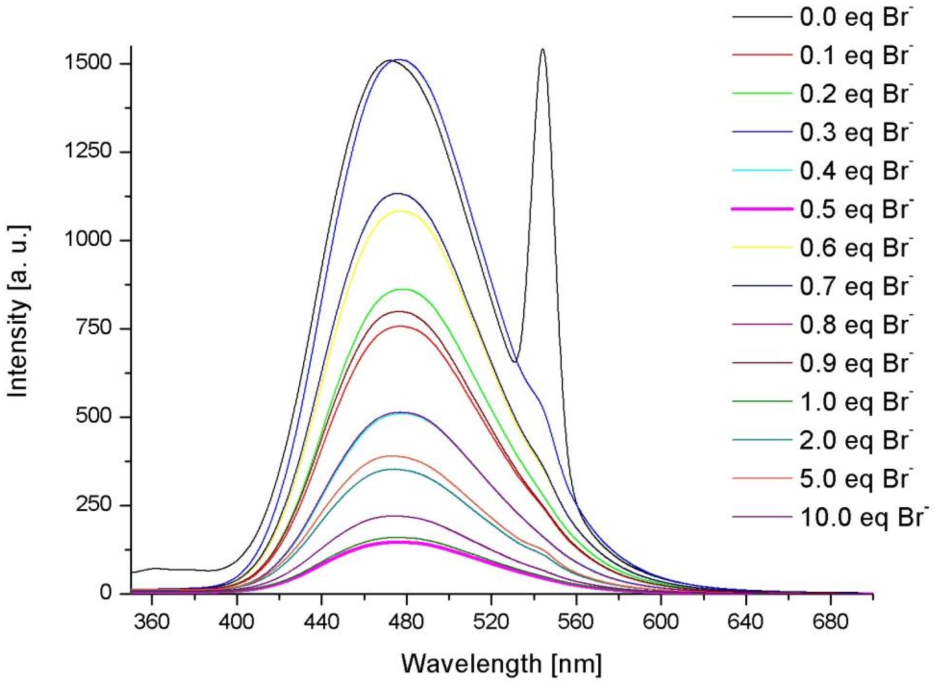

Figure S 24 Emission spectra of **3** in the presence of various molar equivalents of Br^- (DMSO/H_2O 1:1 *v/v*, $C_3 = 2 \cdot 10^{-5}$ M, λ_{ex} = 270 nm).

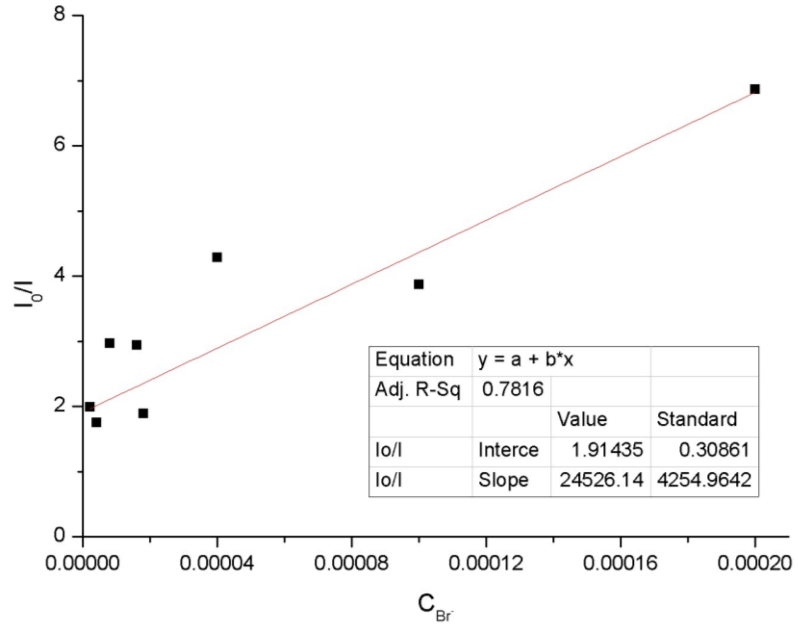

Figure S 25 Stern-Volmer plot regarding the interactions between **3** and **Br^-**. The data for the linear plot are also presented.

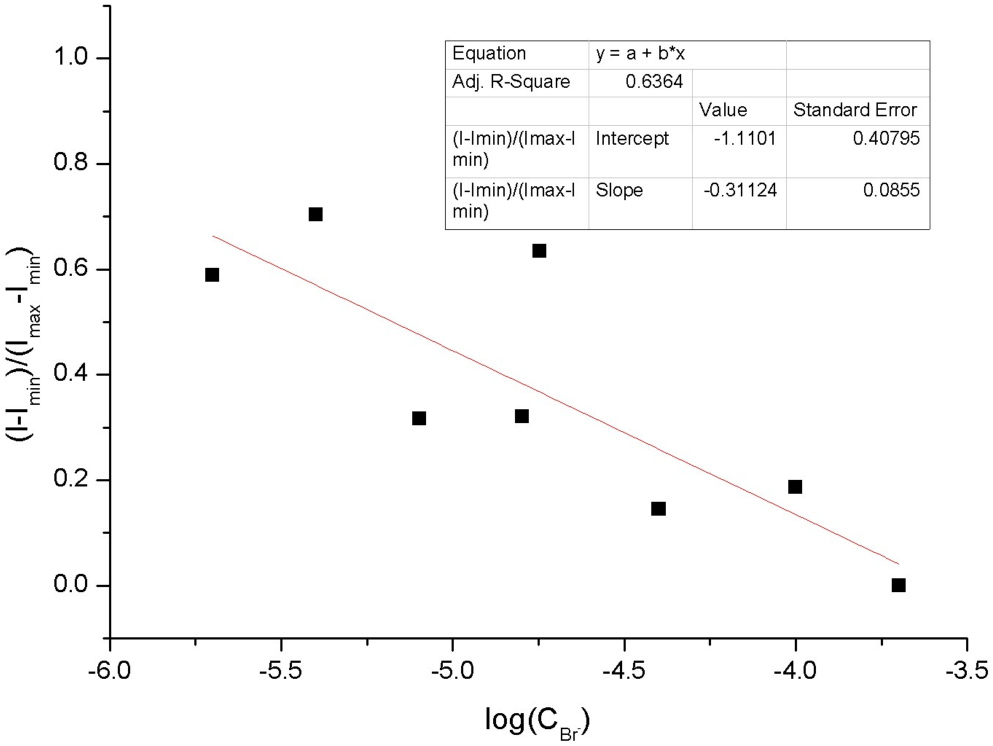

Figure S 26 Plot for $(I-I_{min})/(I_{max}-I_{min})$ versus $\log(C_{Br^-})$ of the interactions between **3** and **Br^-**. The data for the linear plot are also presented.

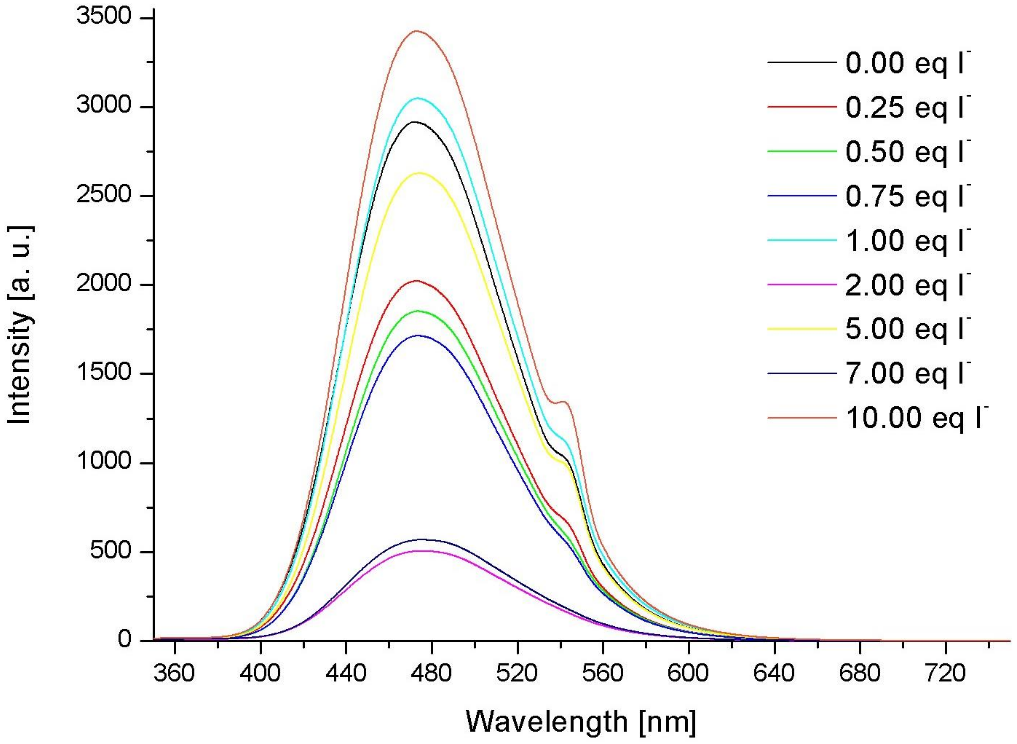

Figure S 27 Emission spectra of **3** in the presence of various molar equivalents of **I**$^-$ (DMSO/H_2O 1:1 *v/v*, $C_3 = 2{\cdot}10^{-5}$ M, λ_{ex} = 270 nm).

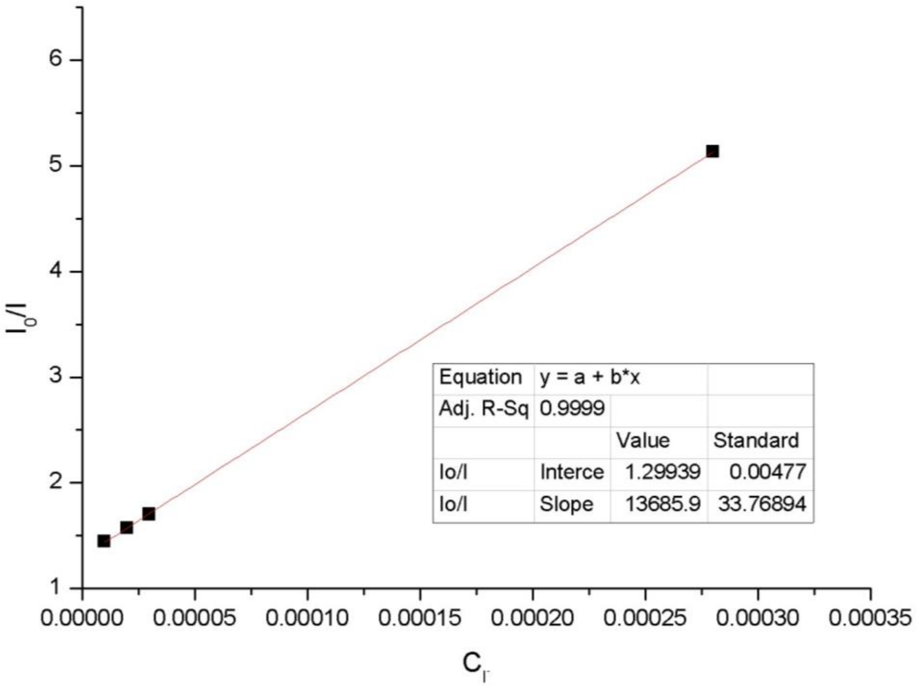

Figure S 28 Stern-Volmer plot regarding the interactions between **3** and **I**$^-$. The data for the linear plot are also presented.

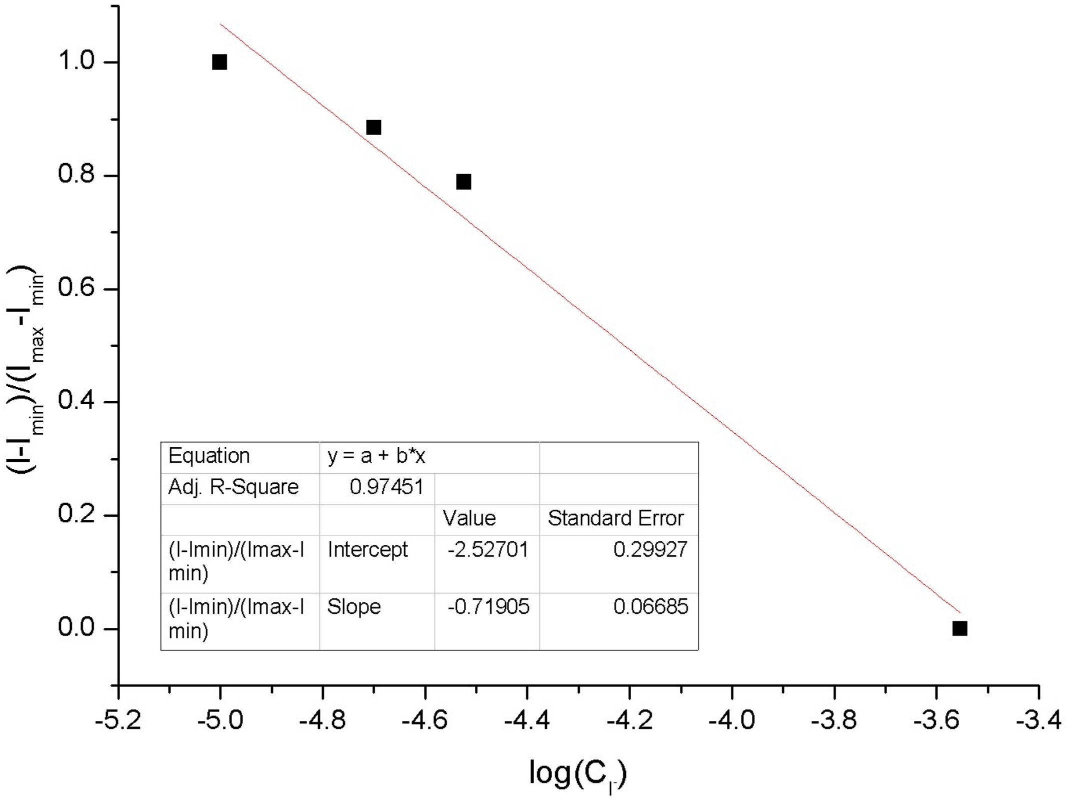

Figure S 29 Plot for $(I\text{-}I_{min})/(I_{max}\text{-}I_{min})$ versus $\log(C_{I^-})$ regarding the interactions between **3** and **I^-**. The data for the linear plot are also presented.

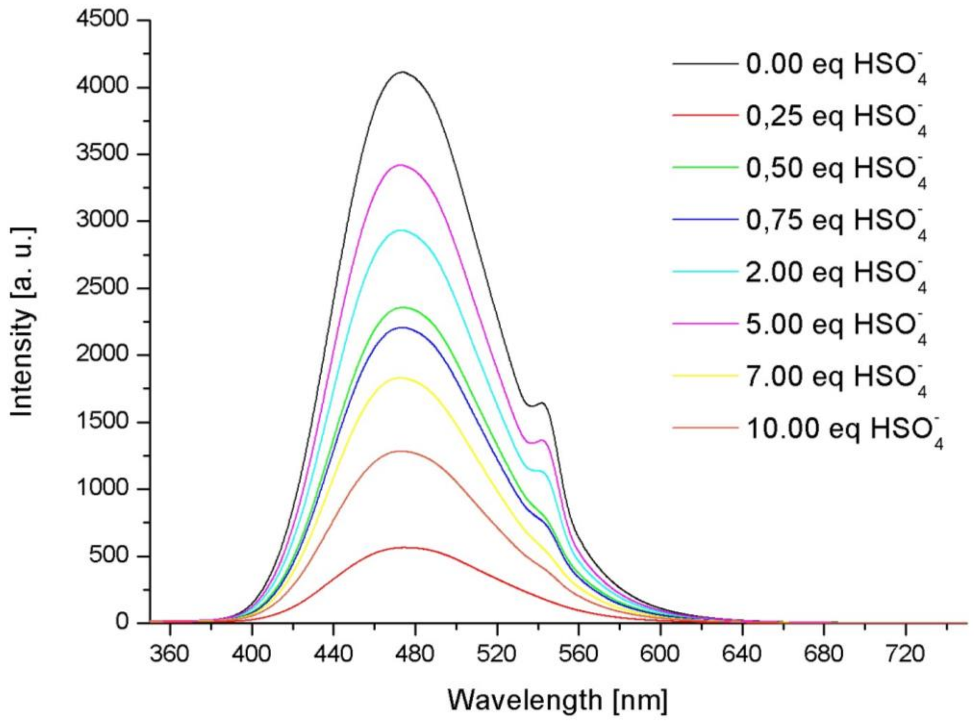

Figure S 30 Emission spectra of **3** in the presence of various molar equivalents of **HSO_4^-** (DMSO/H_2O 1:1 *v/v*, $C_3 = 2\cdot10^{-5}$ M, λ_{ex} = 270 nm).

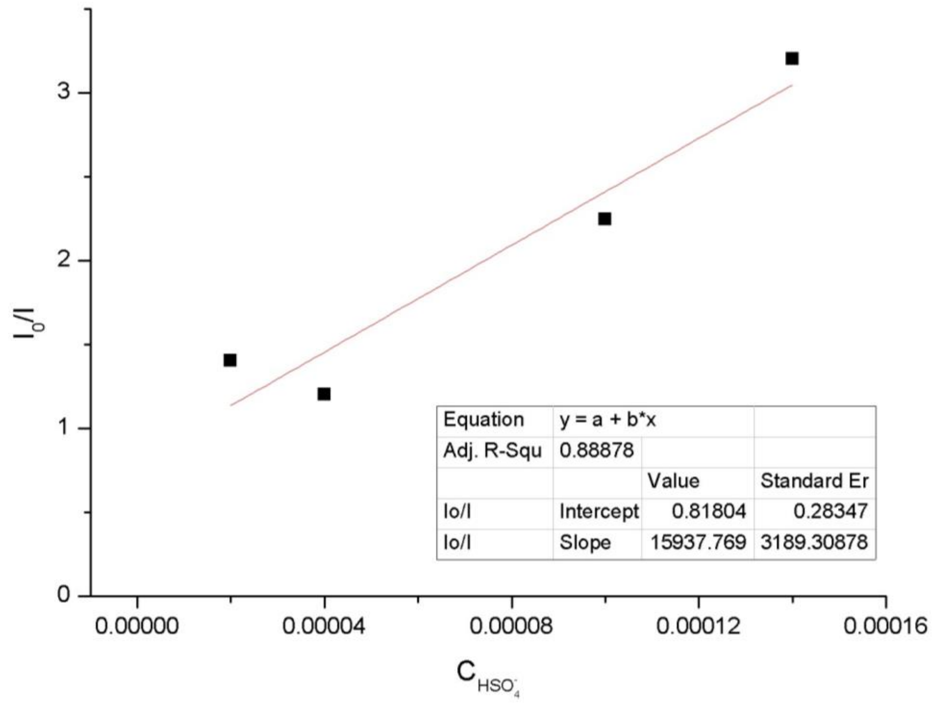

Figure S 31 Stern-Volmer plot regarding the interactions between **3** and **HSO_4^-**. The data for the linear plot are also presented.

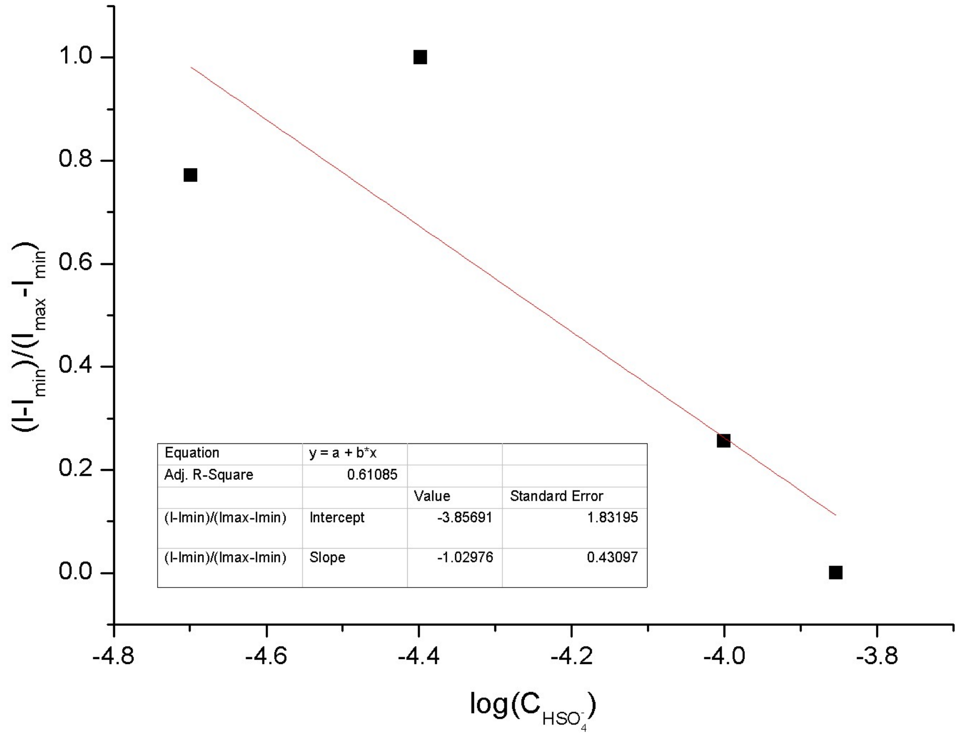

Figure S 32 Plot for $(I-I_{min})/(I_{max}-I_{min})$ versus $\log(C_{HSO_4^-})$ regarding the interactions between **3** and **HSO_4^-**. The data for the linear plot are also presented.

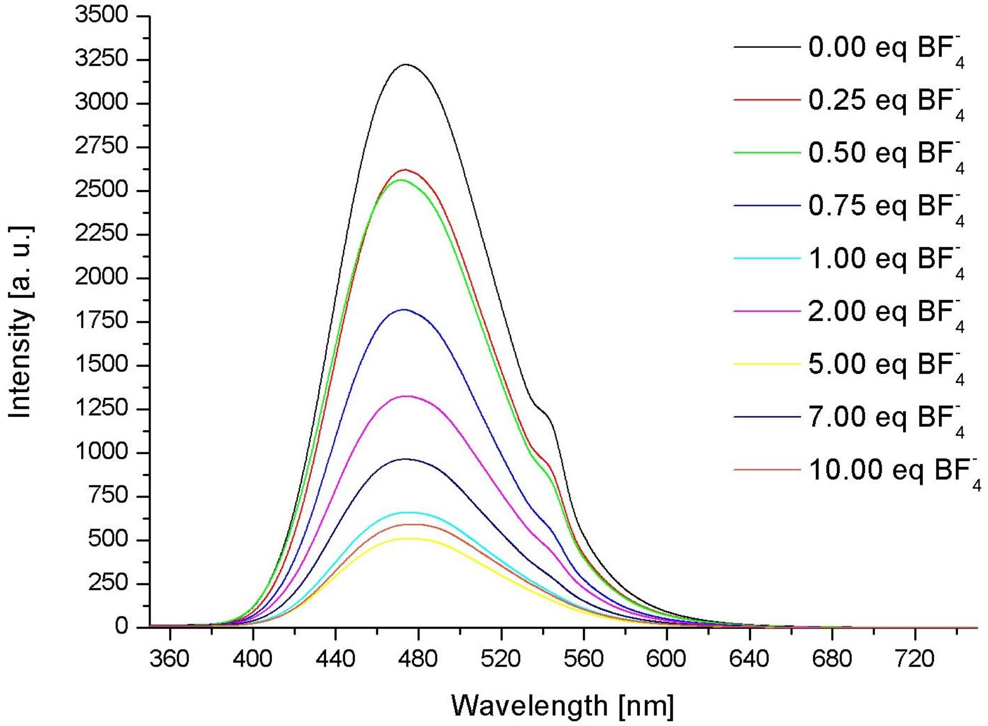

Figure S 33 Emission spectra of **3** in the presence of various molar equivalents of **BF_4^-** (DMSO/H_2O 1:1 *v/v*, $C_3 = 2{\cdot}10^{-5}$ M, λ_{ex} = 270 nm).

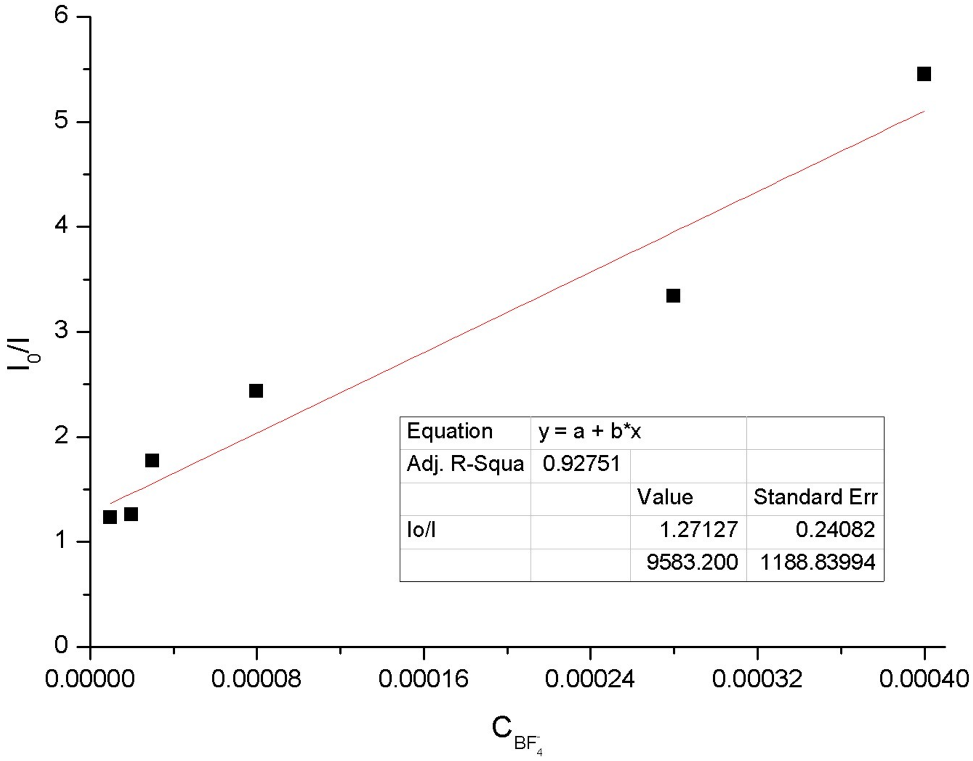

Figure S 34 Stern-Volmer plot regarding the interactions between **3** and **BF_4^-**. The data for the linear plot are also presented.

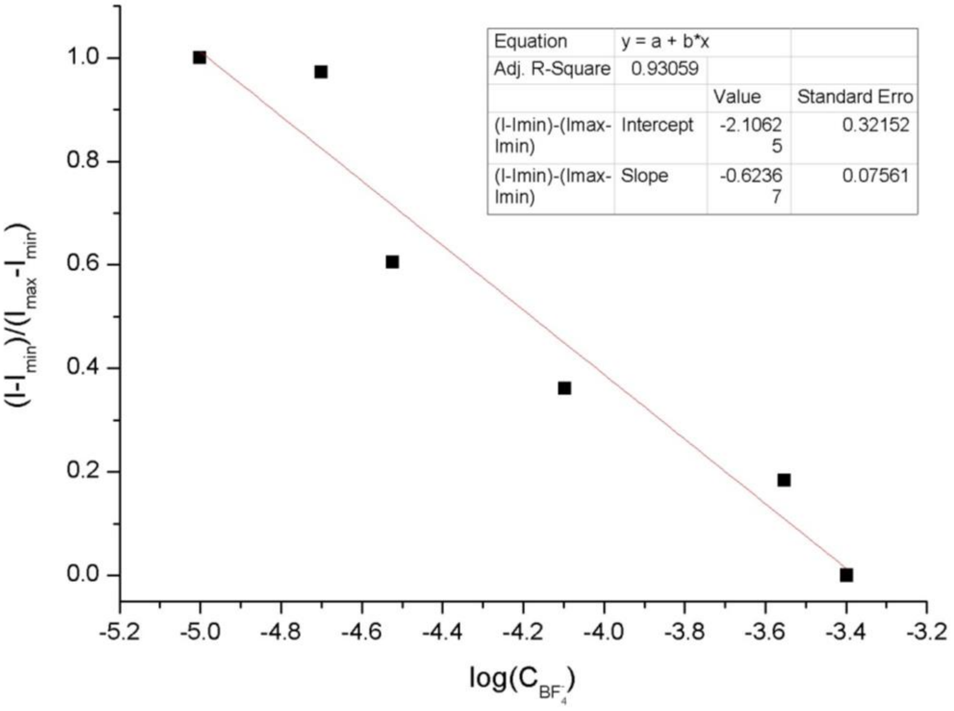

Figure S 35 Plot for (I-I_{min})/(I_{max}-I_{min}) versus $\log(C_{BF_4^-})$ regarding the interactions between **3** and **BF_4^-**. The data for the linear plot are also presented.

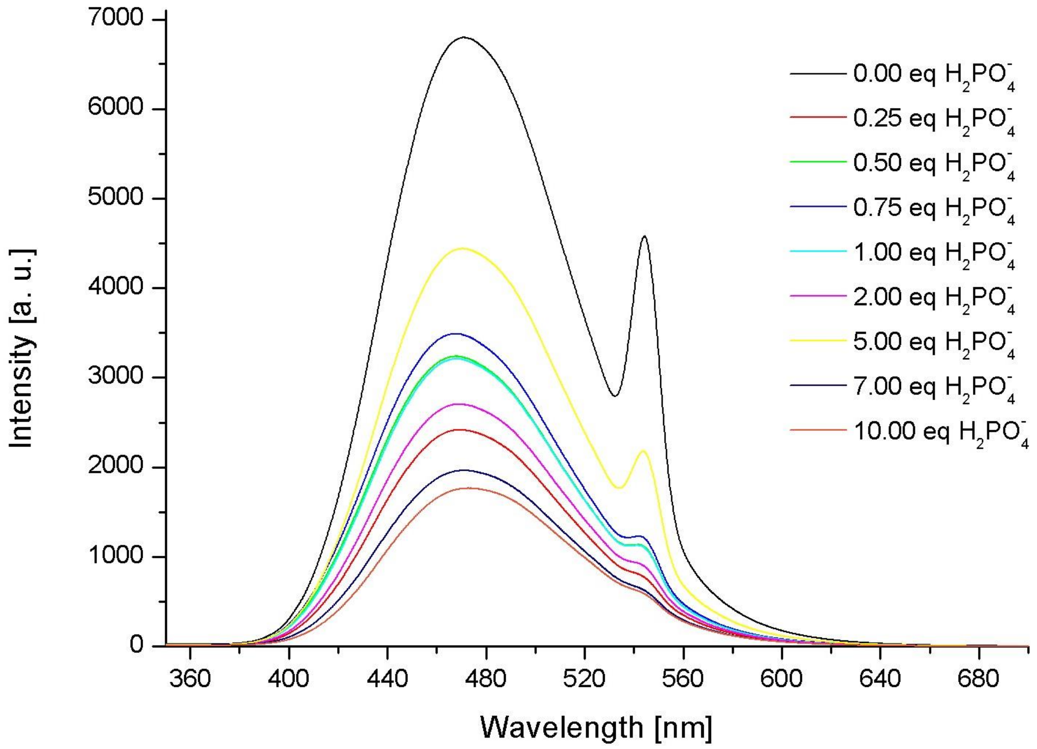

Figure S 36 Emission spectra of **3** in the presence of various molar equivalents of **$H_2PO_4^-$** (DMSO/H_2O 1:1 *v/v*, $C_3 = 2 \cdot 10^{-5}$ M, λ_{ex} = 270 nm).

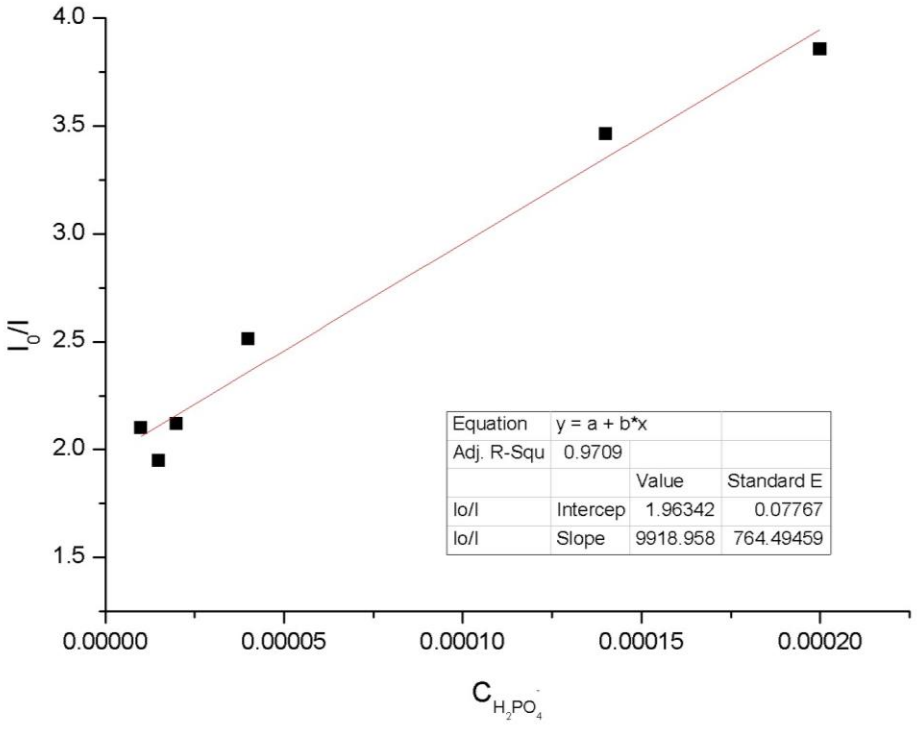

Figure S 37 Stern-Volmer plot regarding the interactions between **3** and $\mathbf{H_2PO_4^-}$. The data for the linear plot are also presented.

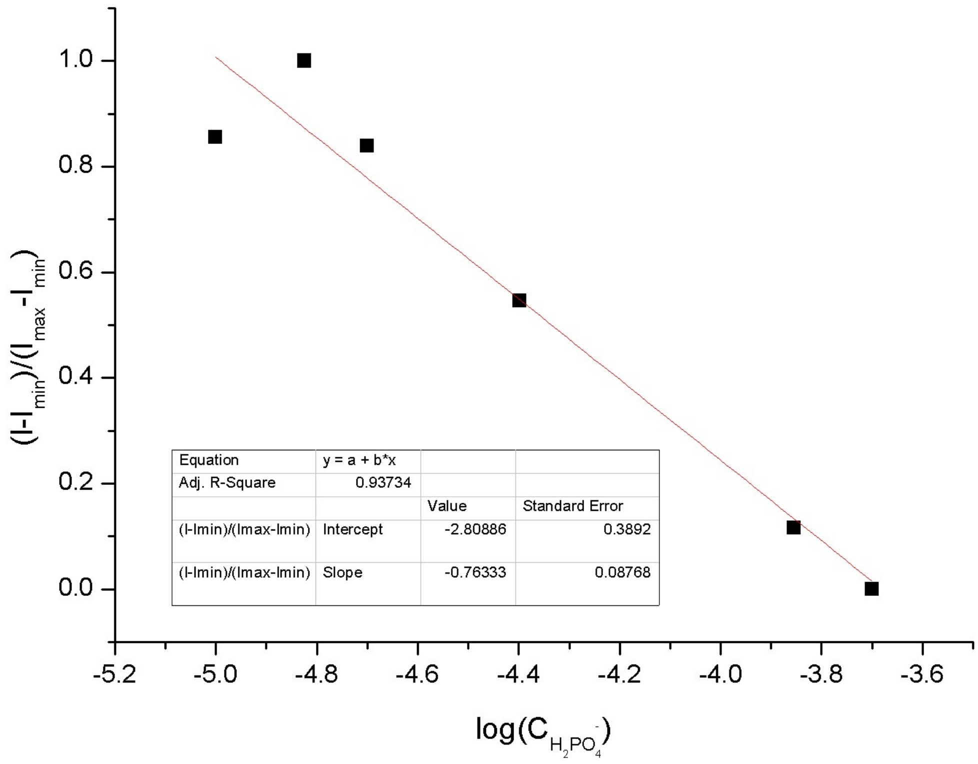

Figure S 38 Plot for $(I-I_{min})/(I_{max}-I_{min})$ versus $\log(C_{H_2PO_4^-})$ regarding the interactions between **3** and $\mathbf{H_2PO_4^-}$. The data for the linear plot are also presented.

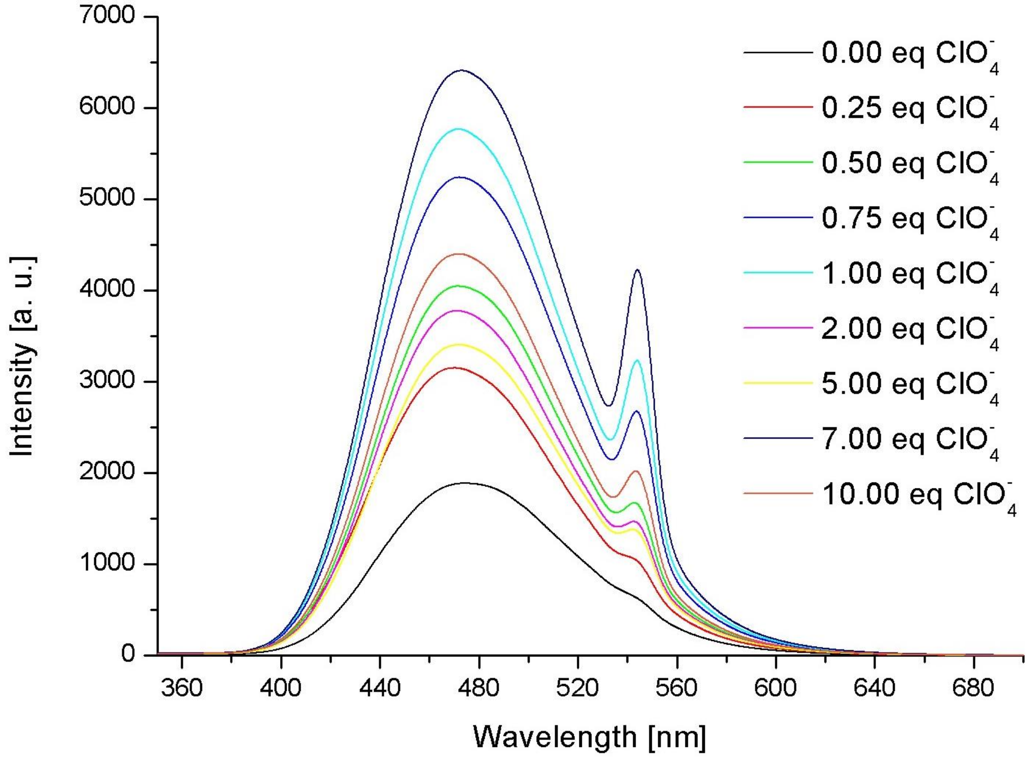

Figure S 39 Emission spectra of **3** in the presence of various molar equivalents of **ClO_4^-** (DMSO/H_2O 1:1 *v/v*, $C_3 = 2\cdot10^{-5}$ M, $\lambda_{ex} = 270$ nm).

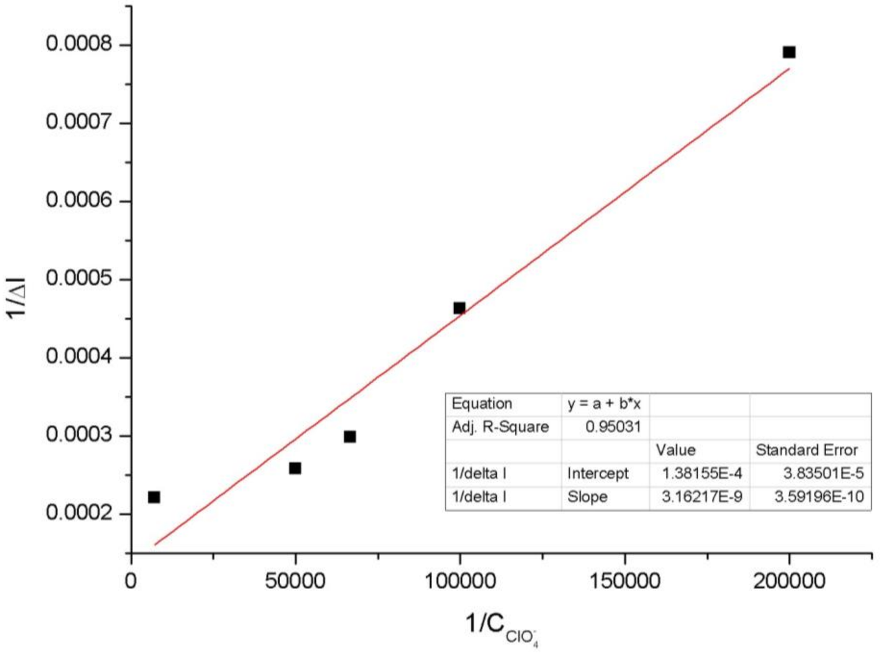

Figure S 40 Benesi-Hildebrand plots regarding the interactions between **3** and **ClO_4^-**. The data for the linear plot are also presented.

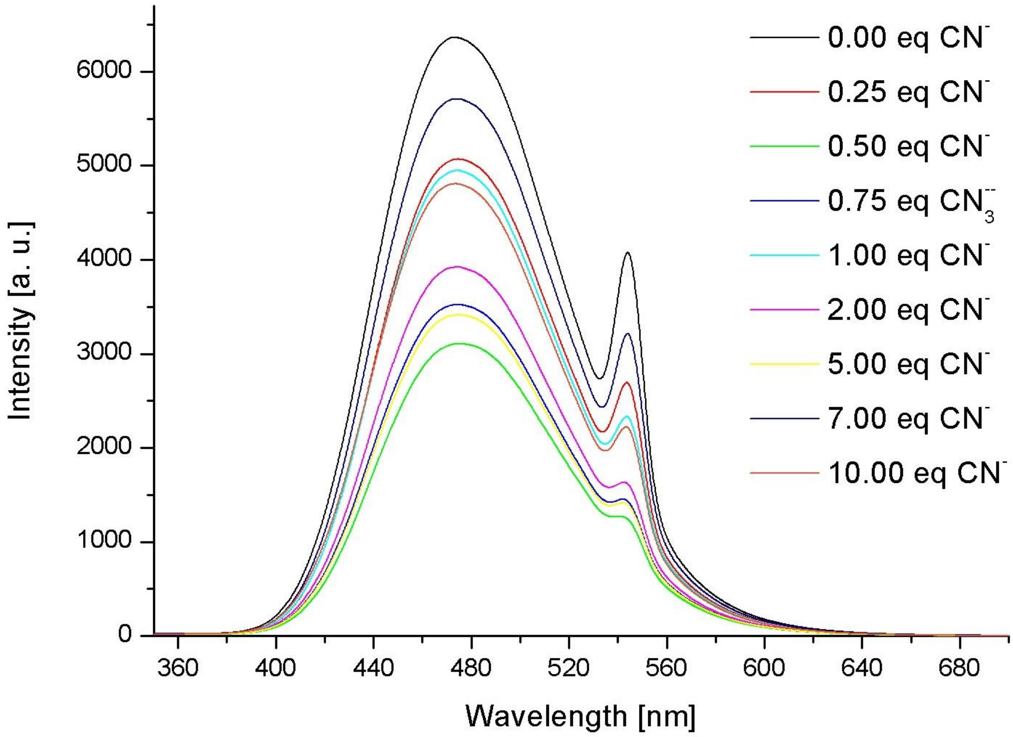

Figure S 41 Emission spectra of **3** in the presence of various molar equivalents of **CN^-** (DMSO/H_2O 1:1 *v/v*, $C_3 = 2\cdot10^{-5}$ M, λ_{ex} = 270 nm).

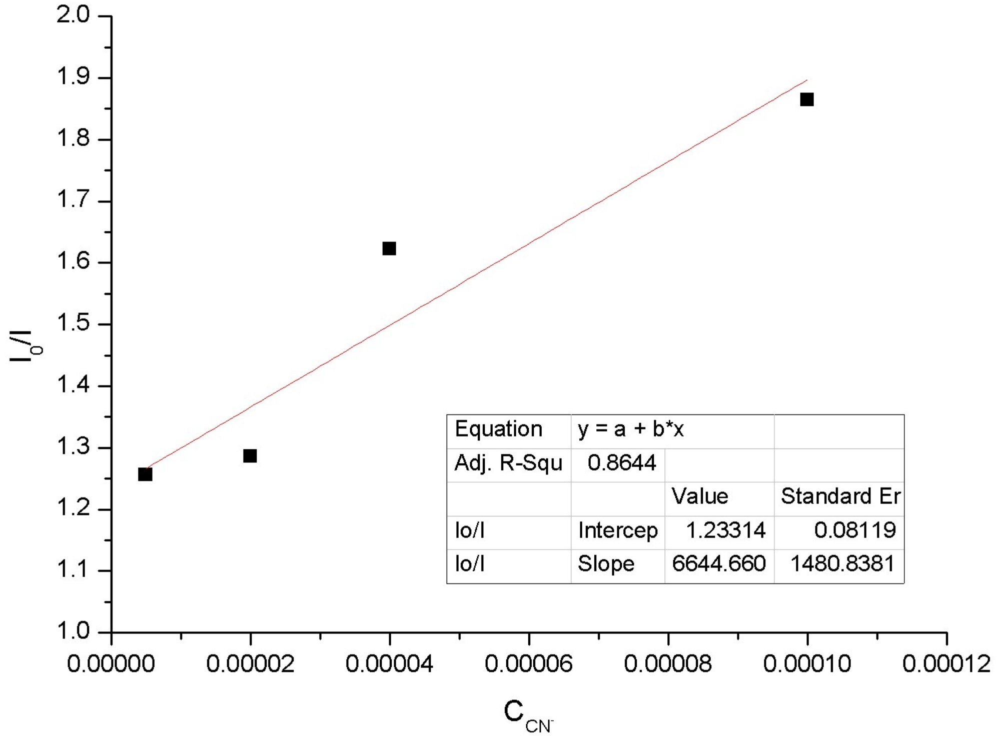

Figure S 42 Stern-Volmer plot regarding the interactions between **3** and **CN^-**. The data for the linear plot are also presented.

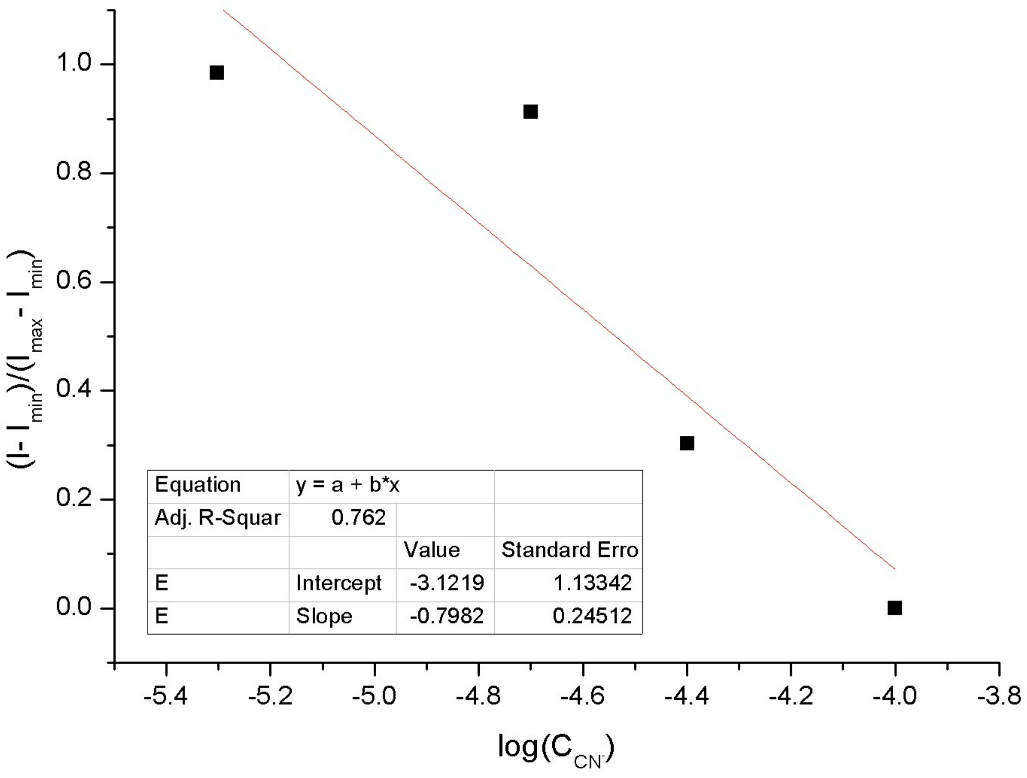

Figure S 43 Plot for $(I\text{-}I_{min})/(I_{max}\text{-}I_{min})$ versus $\log(C_{CN^-})$ regarding the interactions between **3** and **CN$^-$**. The data for the linear plot are also presented.

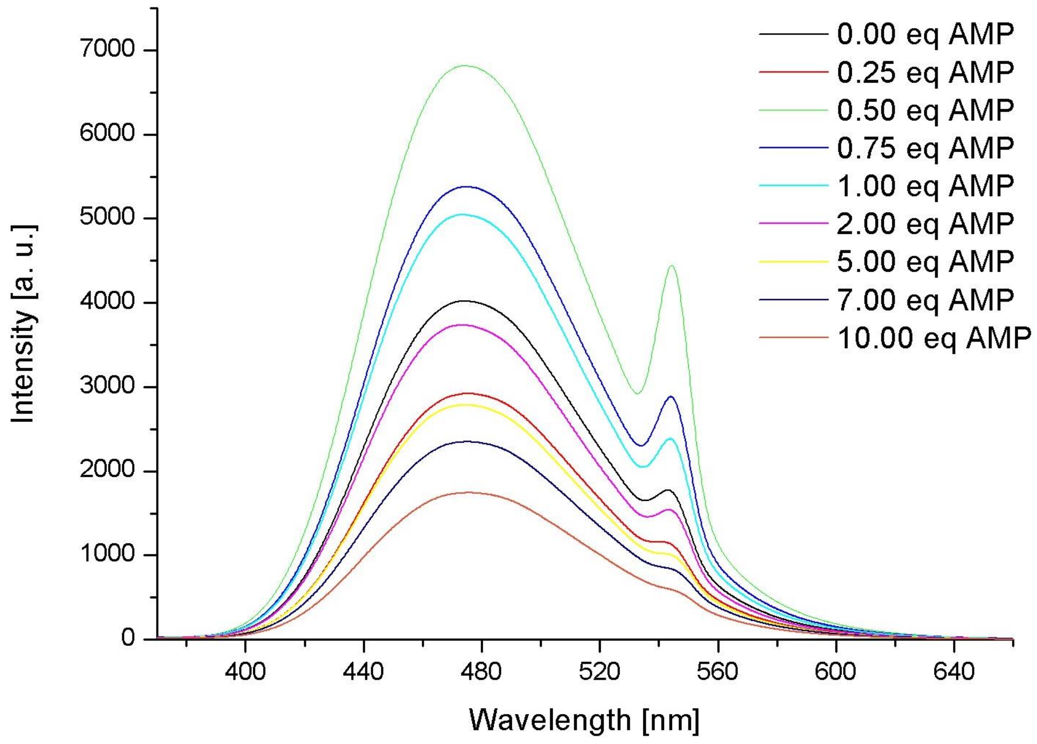

Figure S 44 Emission spectra of **3** in the presence of various molar equivalents of **AMP** (DMSO/H_2O 1:1 *v/v*, $C_3 = 2\cdot10^{-5}$ M, $\lambda_{ex} = 270$ nm).

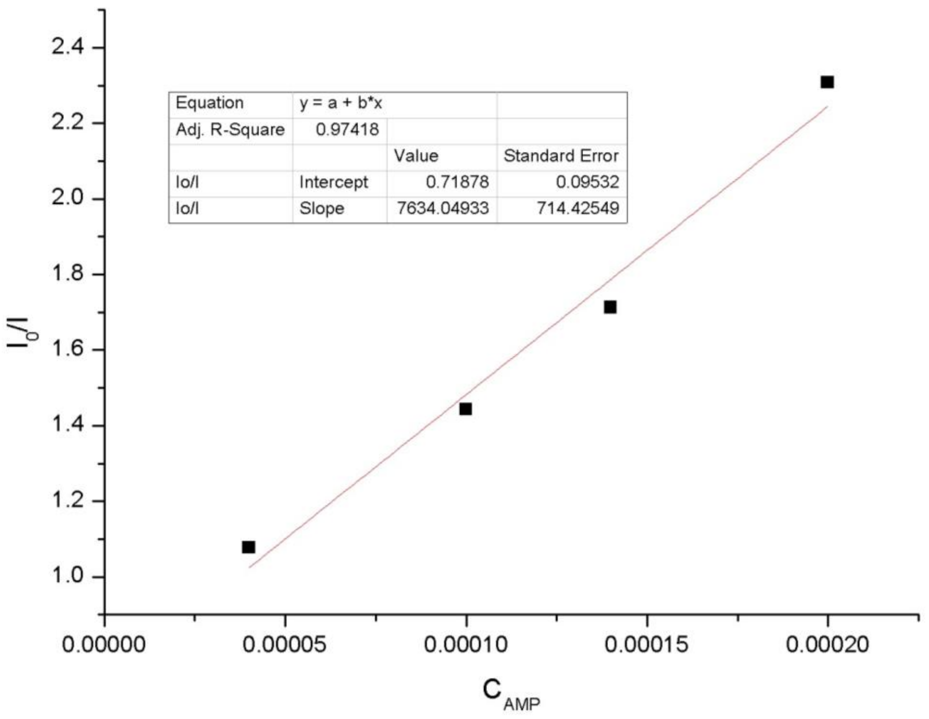

Figure S 45 Stern-Volmer plot regarding the interactions between **3** and **AMP**. The data for the linear plot are also presented.

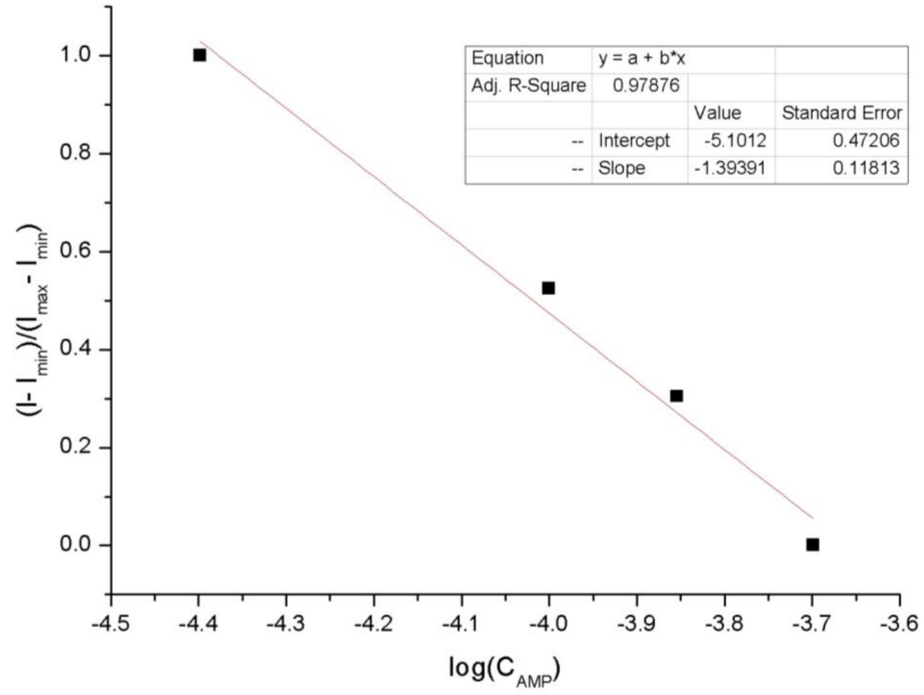

Figure S 46 Plot for $(I-I_{min})/(I_{max}-I_{min})$ versus $\log(C_{AMP})$ of the interactions between **3** and **AMP**. The data for the linear plot are also presented.

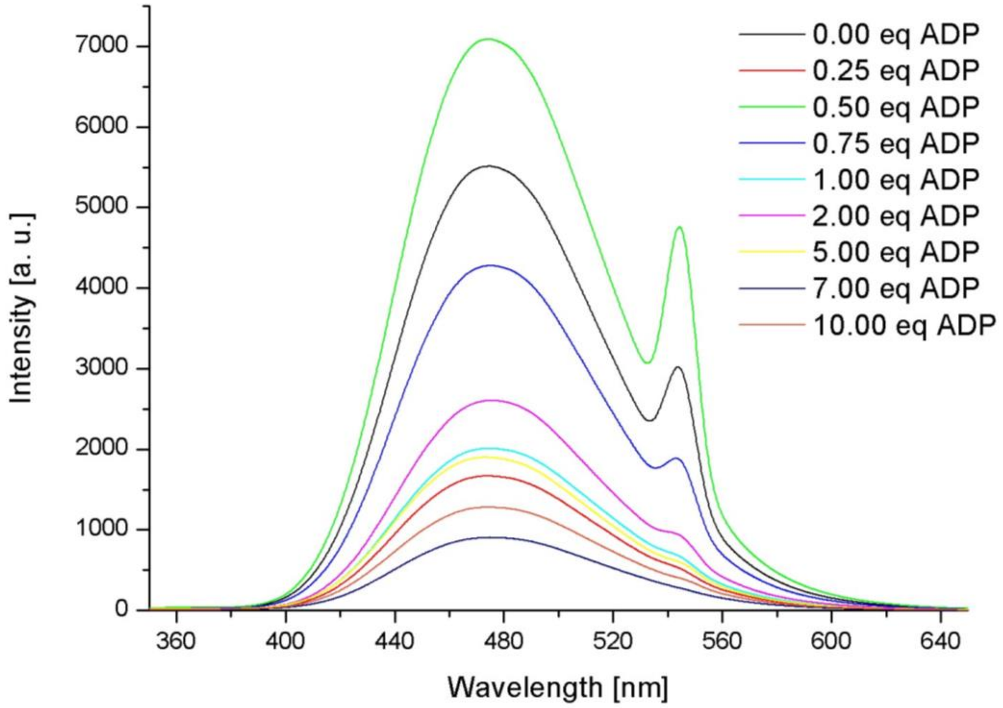

Figure S 47 Emission spectra of **3** in the presence of various molar equivalents of **ADP** (DMSO/H_2O 1:1 *v/v*, $C_3 = 2\cdot10^{-5}$ M, λ_{ex} = 270 nm).

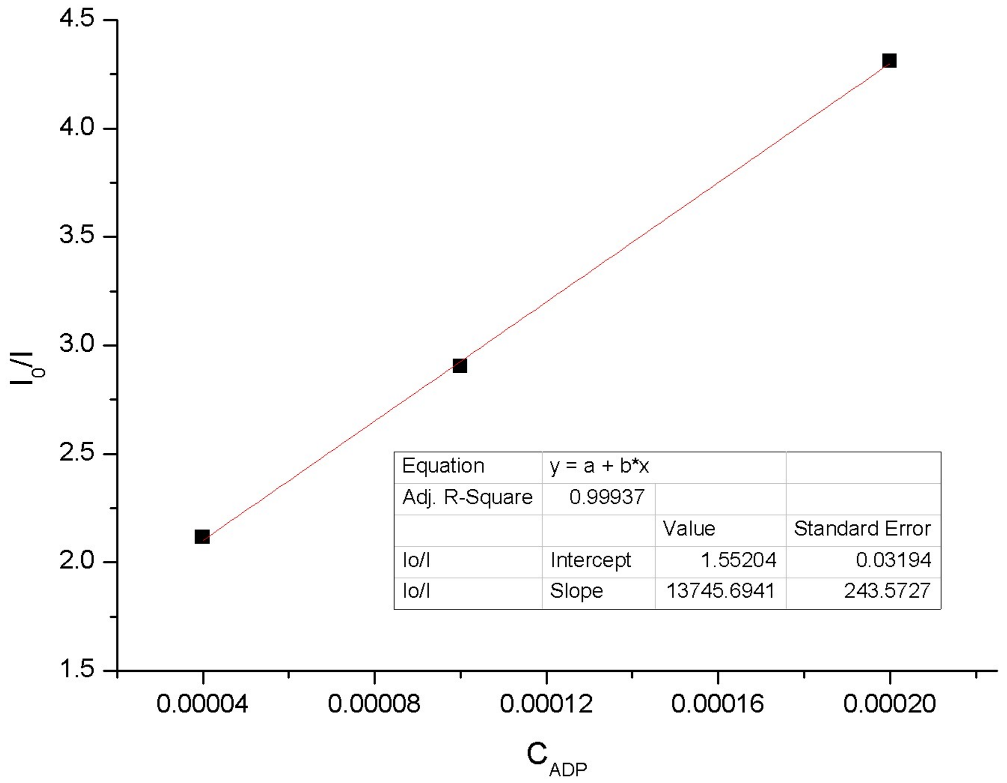

Figure S 48 Stern-Volmer plot regarding the interactions between **3** and **ADP**. The data for the linear plot are also presented.

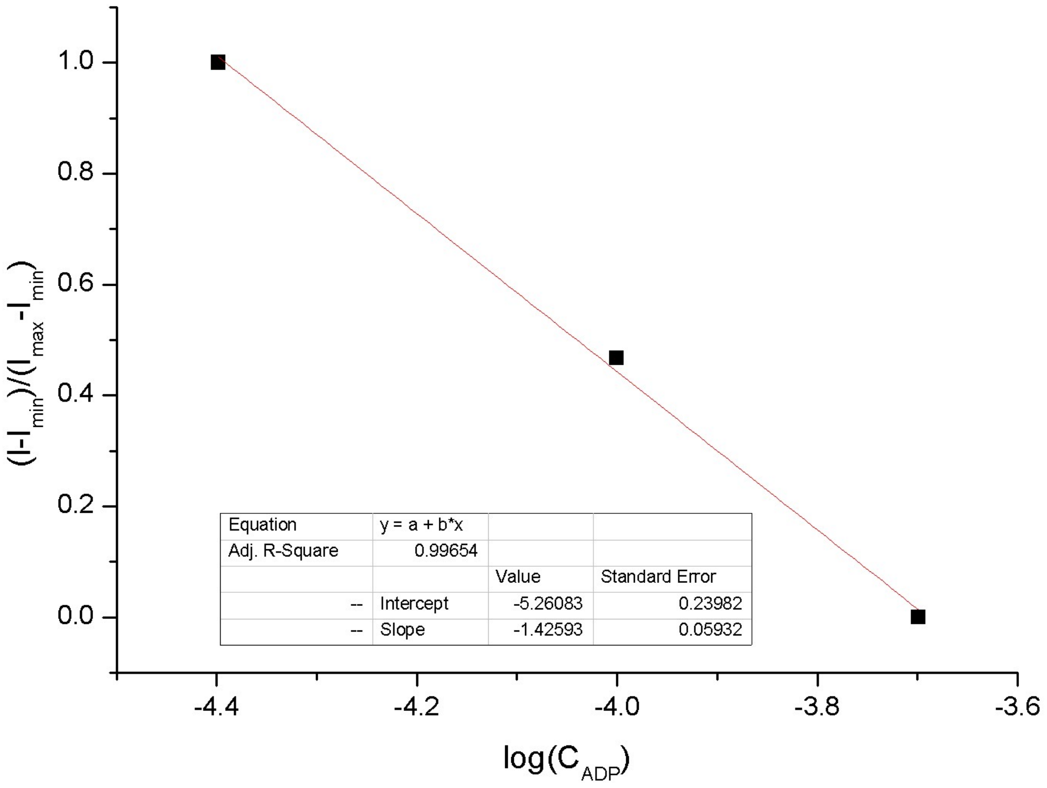

Figure S 49 Plot for $(I\text{-}I_{min})/(I_{max}\text{-}I_{min})$ versus $\log(C_{AMP})$ of the interactions between **3** and **ADP**. The data for the linear plot are also presented.

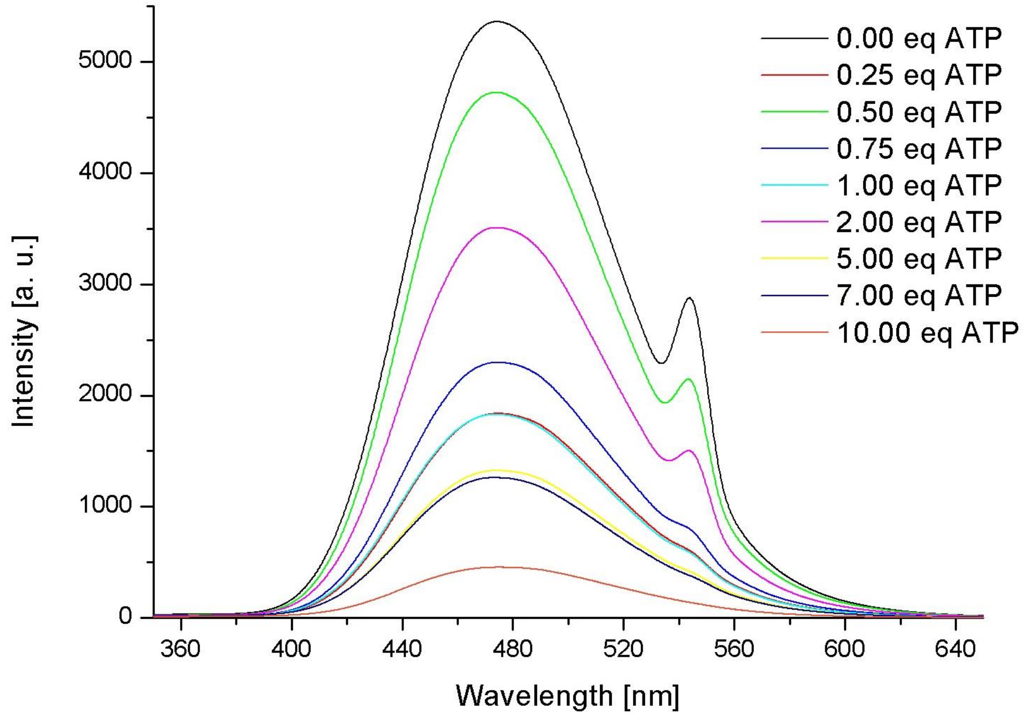

Figure S 50 Emission spectra of **3** in the presence of various molar equivalents of **ATP** (DMSO/H_2O 1:1 *v/v*, $C_3 = 2{\cdot}10^{-5}$ M, λ_{ex} = 270 nm).

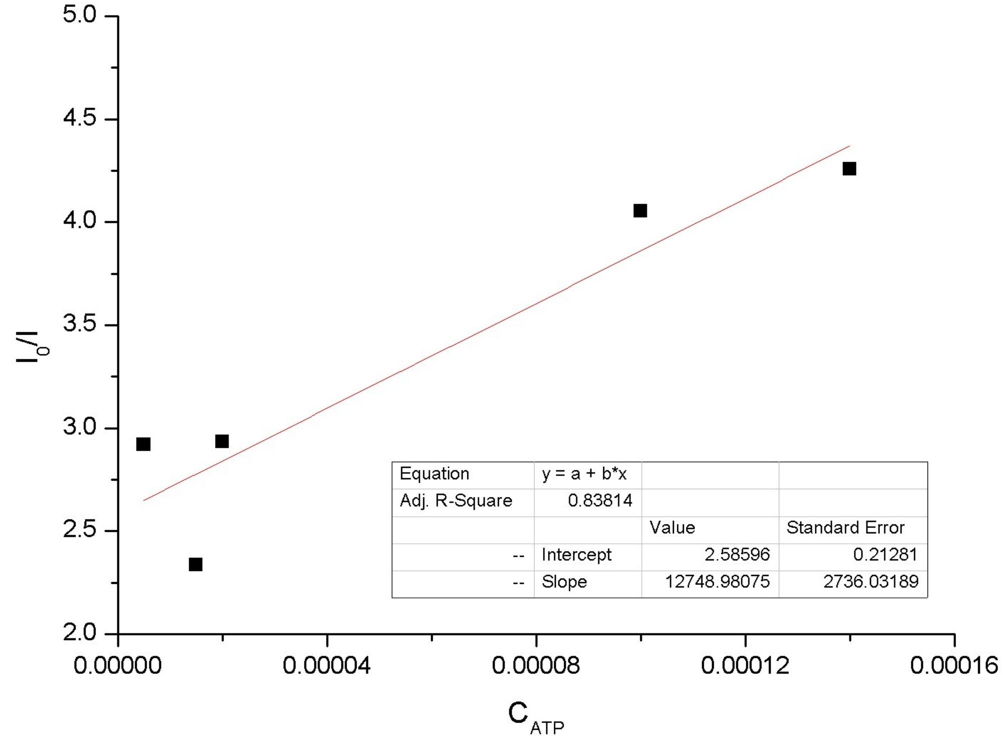

Figure S 51 Stern-Volmer plot of the interactions between **3** and **ATP**. The data for the linear plot are also presented.

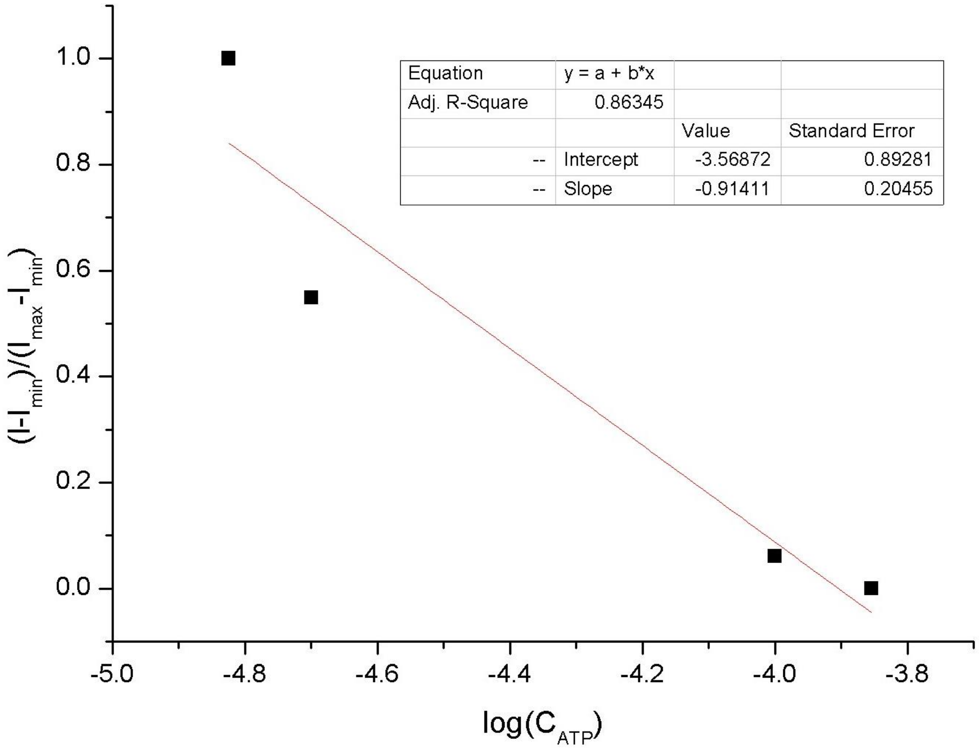

Figure S 52 Plot for $(I-I_{min})/(I_{max}-I_{min})$ versus $\log(C_{AMP})$ of the interactions between **3** and **ATP**. The data for the linear plot are also presented.

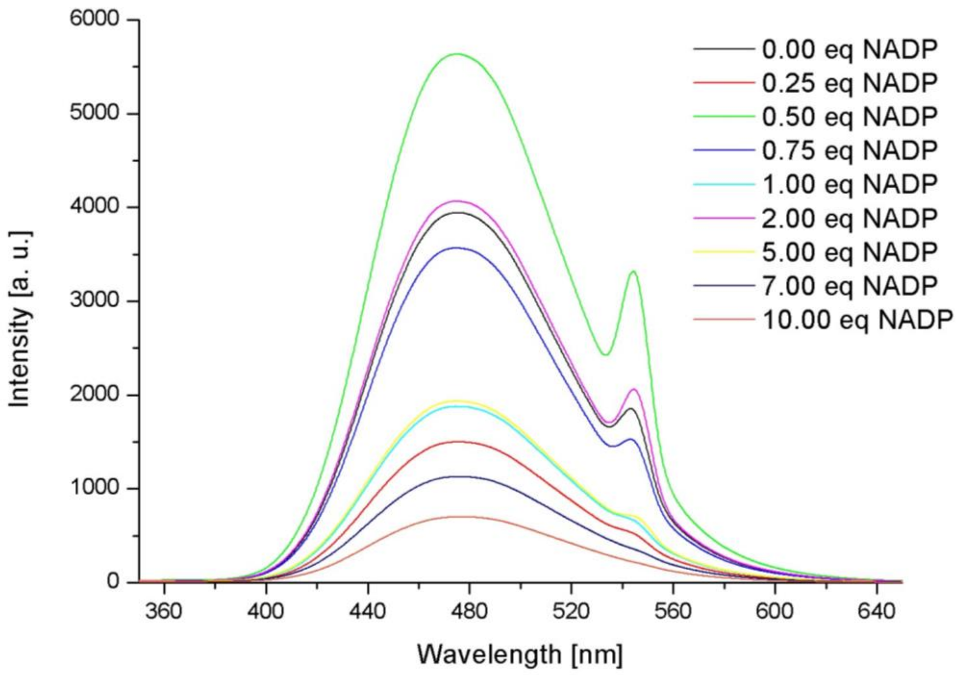

Figure S 53 Emission spectra of **3** in the presence of various molar equivalents of **NADP** (DMSO/H_2O 1:1 *v/v*, C_3 = $2 \cdot 10^{-5}$ M, λ_{ex} = 270 nm).

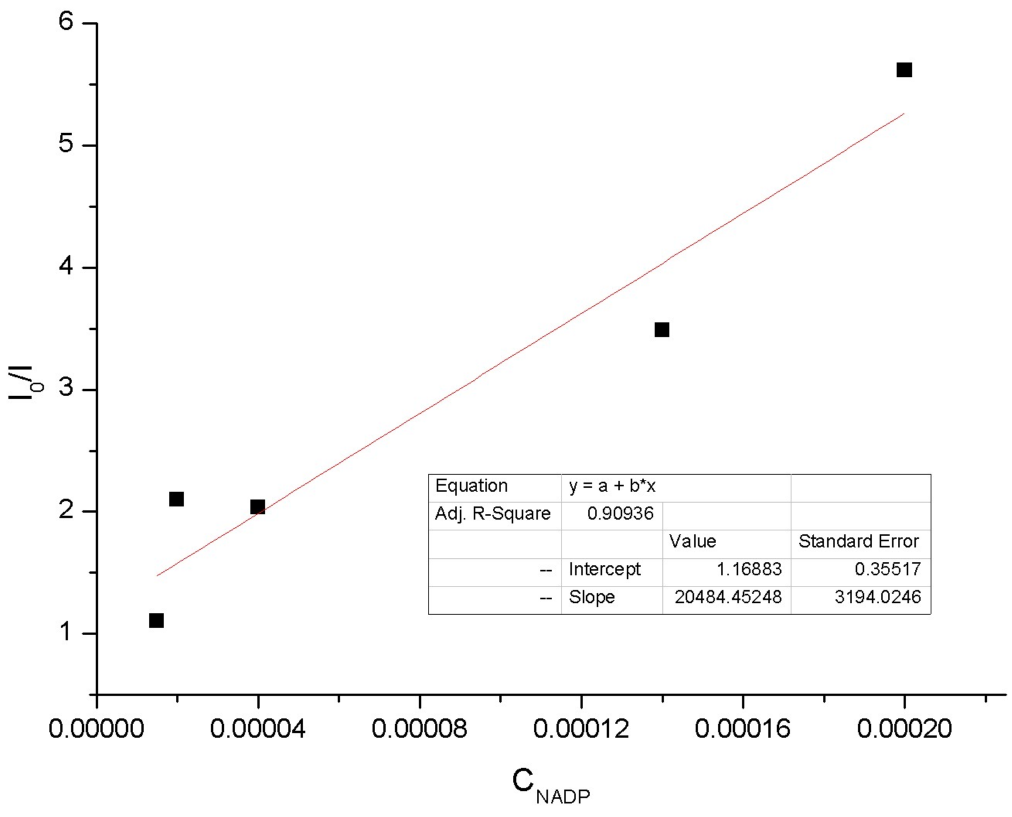

Figure S 54 Stern-Volmer plot of the interactions between **3** and **NADP**. The data for the linear plot are also presented.

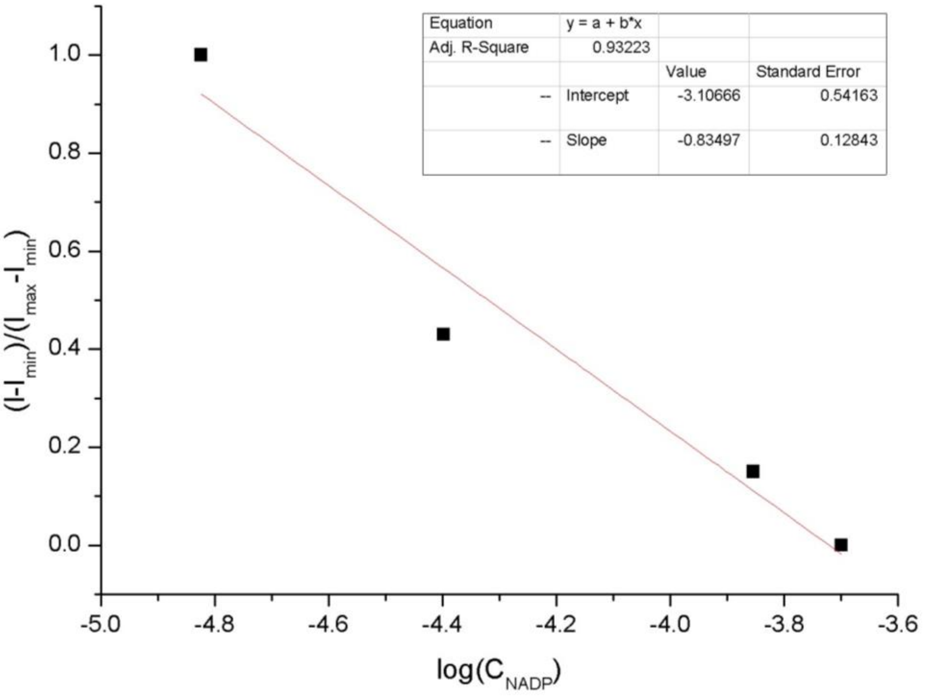

Figure S 55 Plot for $(I\text{-}I_{min})/(I_{max}\text{-}I_{min})$ versus $\log(C_{AMP})$ regarding the interactions between **3** and **NADP**. The data for the linear plot are also presented.

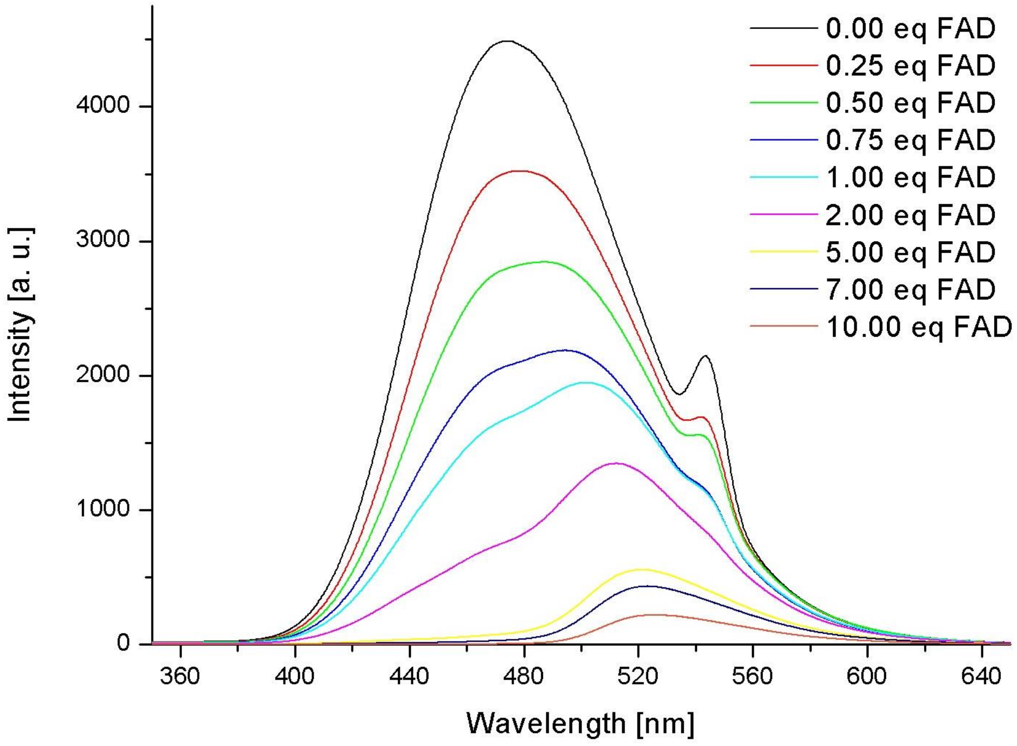

Figure S 56 Emission spectra of **3** in the presence of various molar equivalents of **FAD** (DMSO/H_2O 1:1 *v/v*, $C_3 = 2\cdot10^{-5}$ M, λ_{ex} = 270 nm).

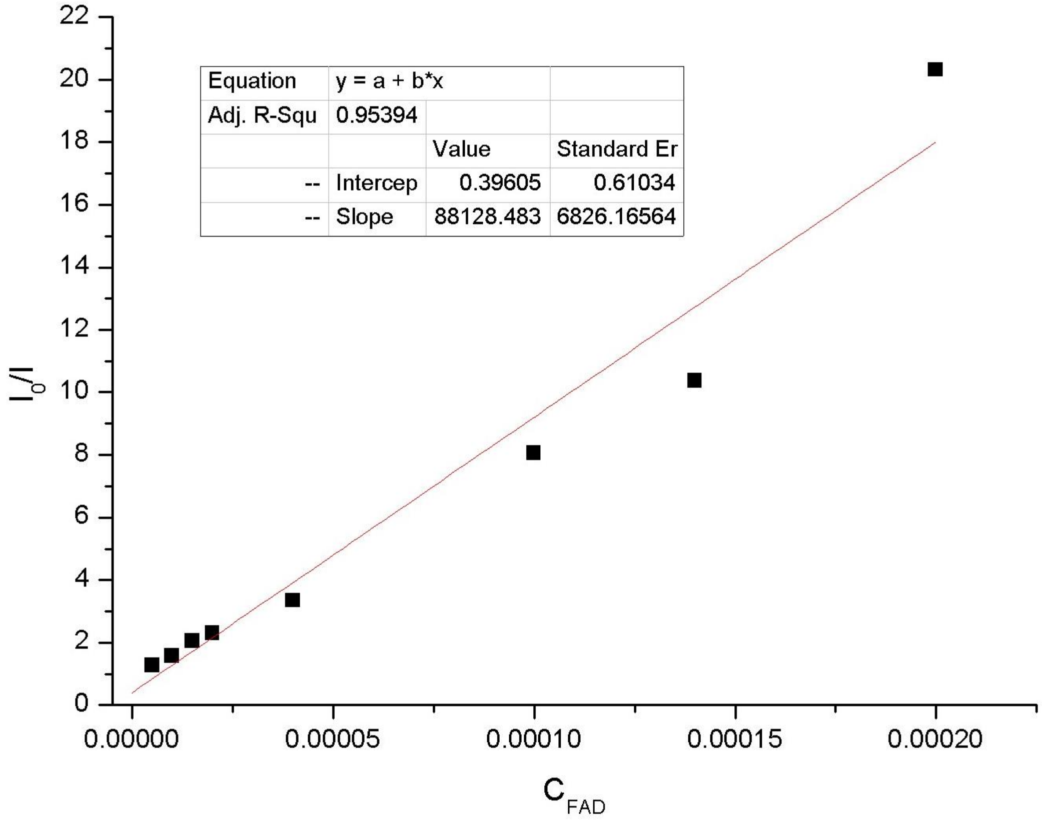

Figure S 57 Stern-Volmer plot of the interactions between **3** and **FAD**. The data for the linear plot are also presented.

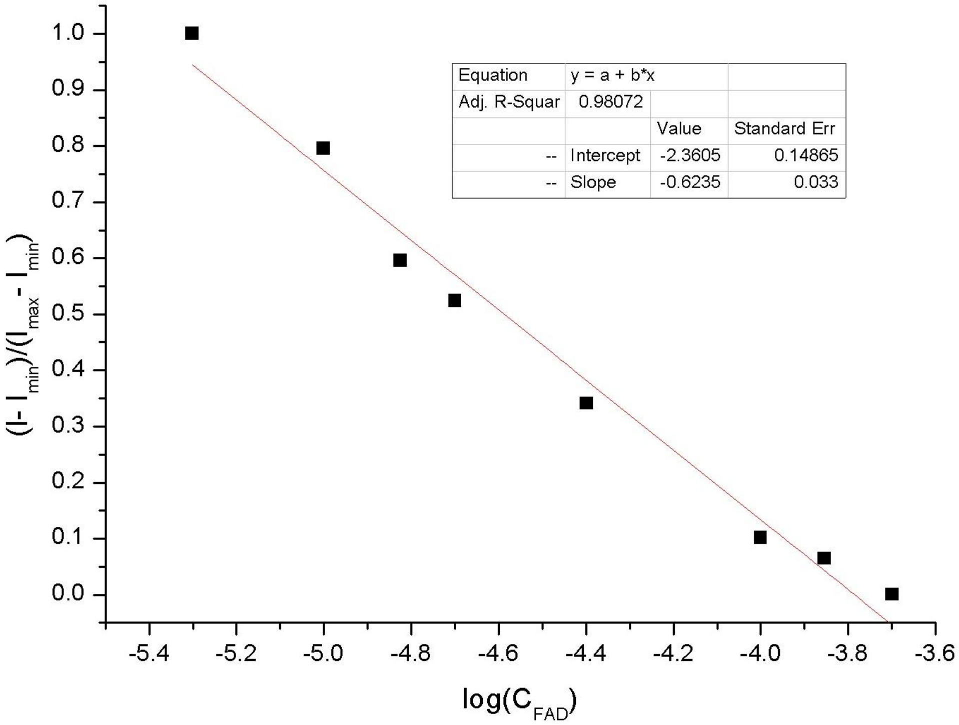

Figure S 58 Plot for $(I-I_{min})/(I_{max}-I_{min})$ versus $\log(C_{AMP})$ of the interactions between **3** and **NADP**. The data for the linear plot are also presented.

S6. DLS measurements

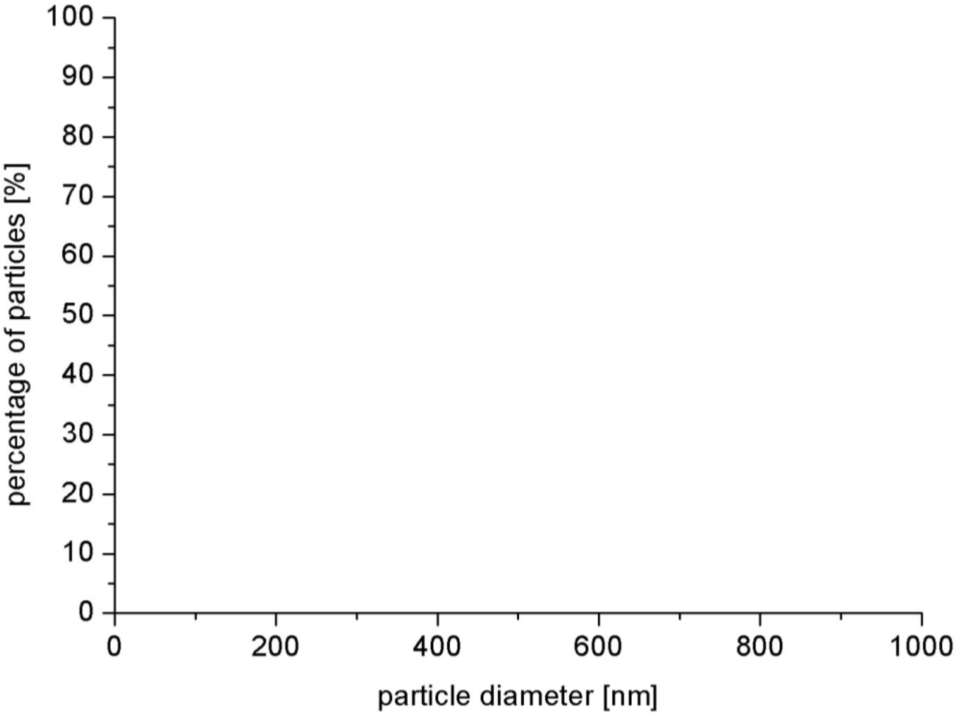

Figure S 59 Size distribution pattern of **3** in DMSO

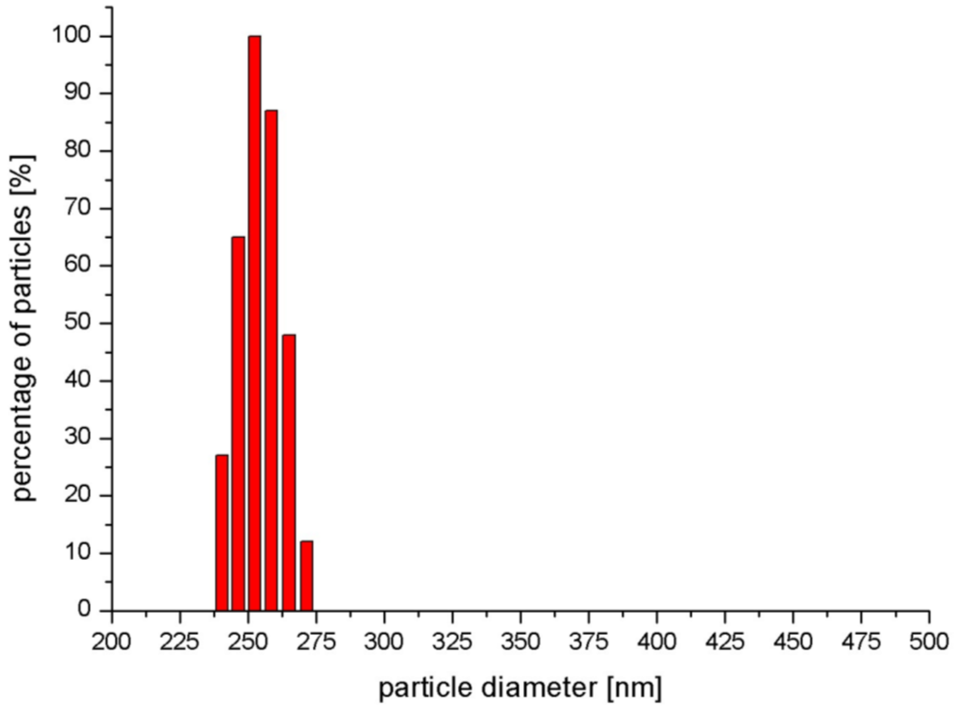

Figure S 60 Size distribution pattern of **3** in DMSO/H_2O = 1/1 *v/v* system

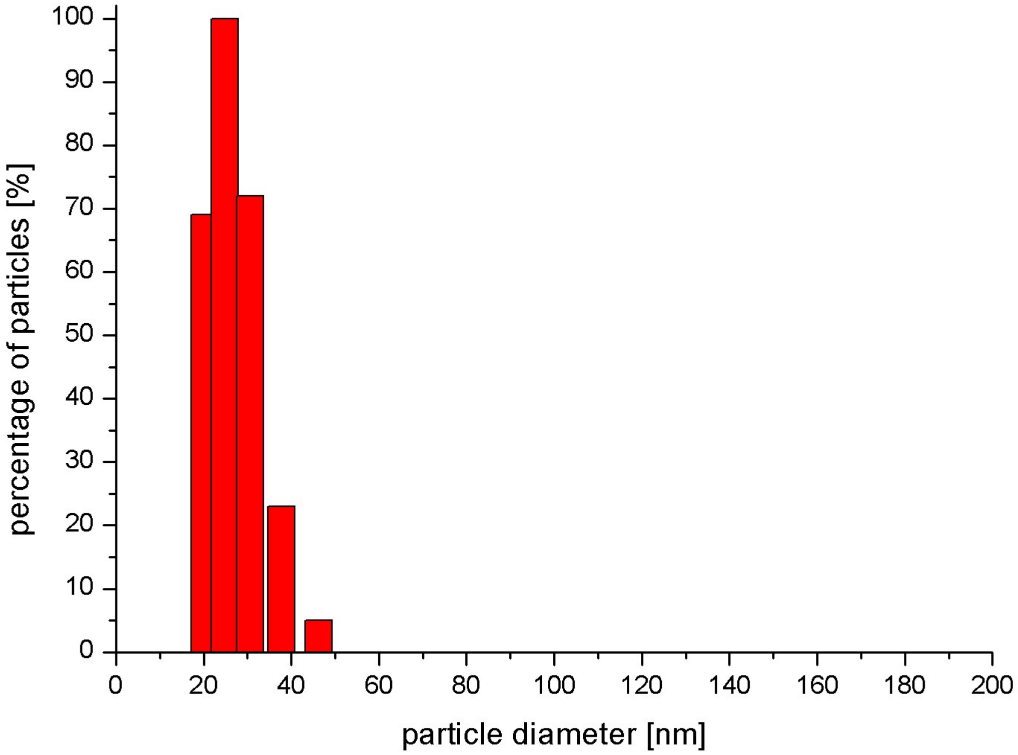

Figure S 61 Size distribution pattern of **3** in DMSO/H_2O = 9/1 *v/v* system

S7 SEM images

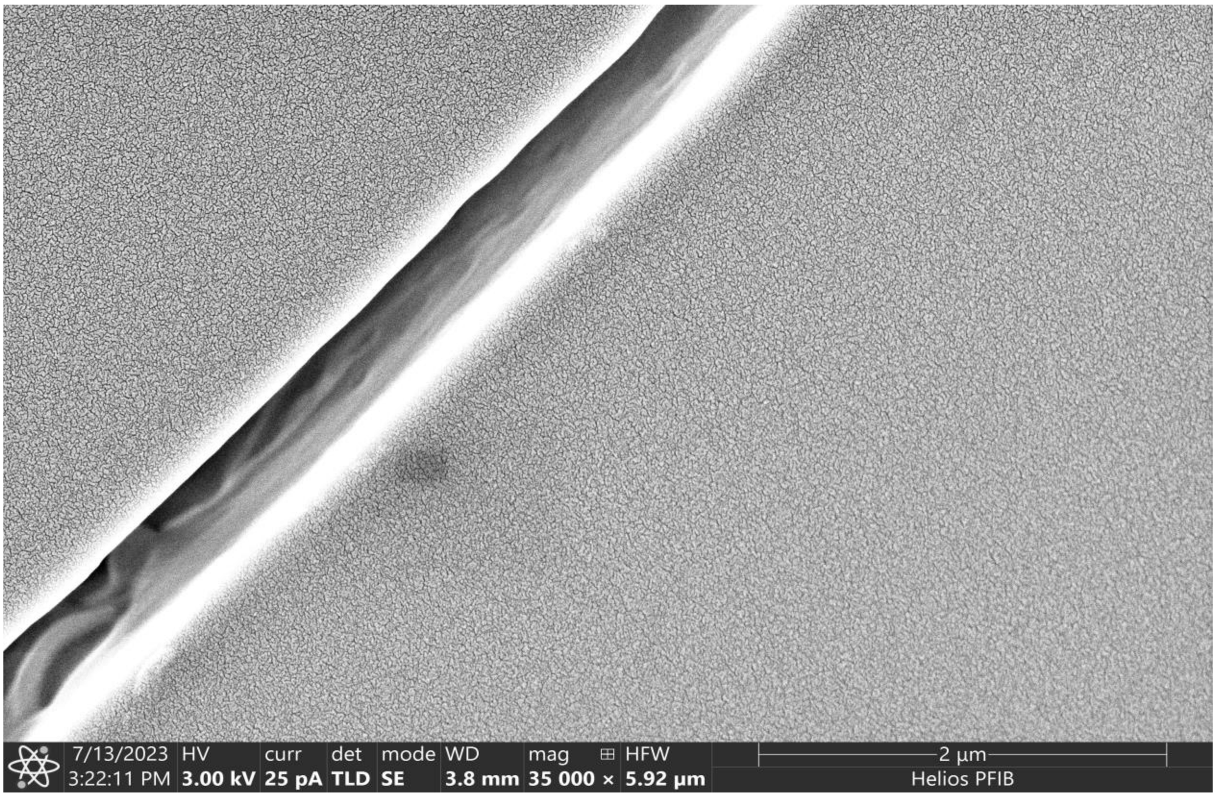

Figure S 62 SEM image of solid **3** obtained after column chromatography

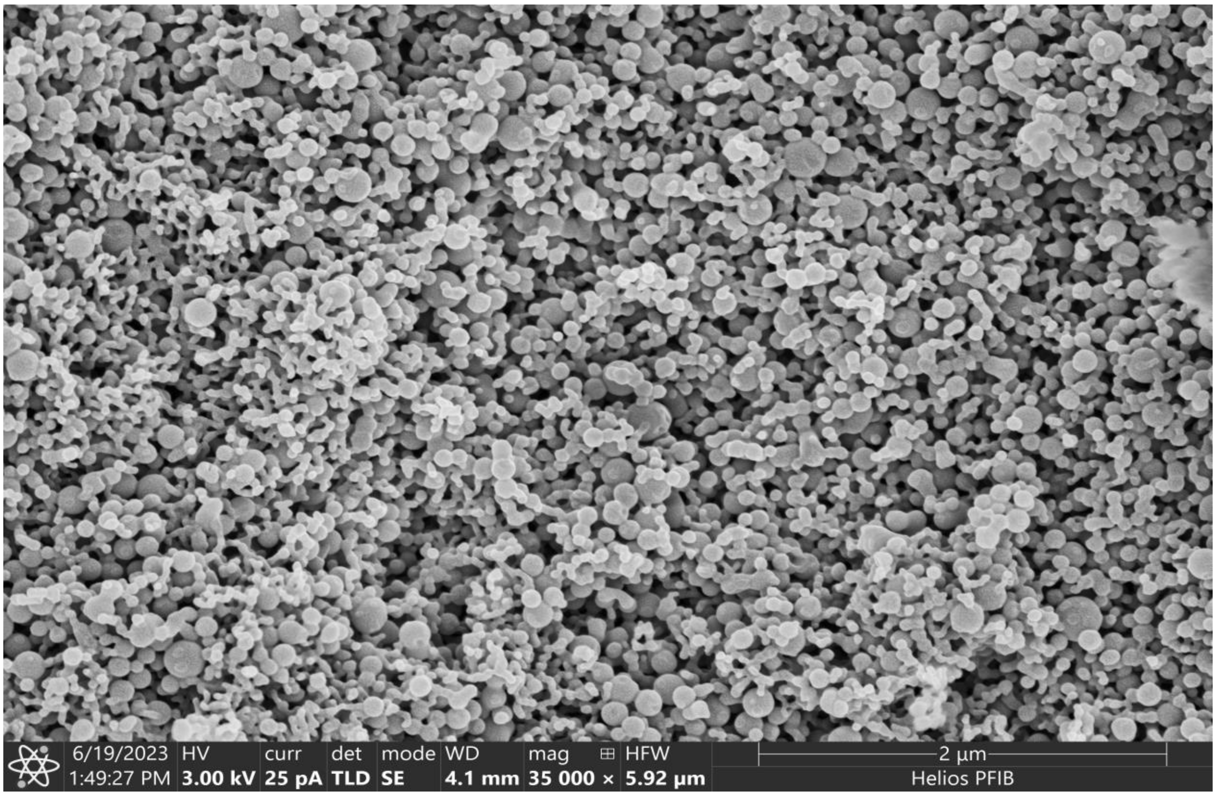

Figure S 63 SEM image of dried aggregates of **3**

S8 Supporting references

1. Brouwer, A. M. Standards for photoluminescence quantum yield measurements in solution (IUPAC Technical Report). *Pure Appl. Chem.* **83**, 2213–2228 (2011).

2. Würth, C., Grabolle, M., Pauli, J., Spieles, M. & Resch-Genger, U. Relative and absolute determination of fluorescence quantum yields of transparent samples. *Nat. Protoc.* **8**, 1535–1550 (2013).

3. Brouwer, A. M. Standards for photoluminescence quantum yield measurements in solution (IUPAC Technical Report). *Pure Appl. Chem.* **83**, 2213–2228 (2011).

4. Benesi, H. A. & Hildebrand, J. H. A Spectrophotometric Investigation of the Interaction of Iodine with Aromatic Hydrocarbons. *J. Am. Chem. Soc.* **71**, 2703–2707 (1949).

5. Goswami, S. *et al.* A highly selective and sensitive probe for colorimetric and fluorogenic detection of Cd2+ in aqueous media. *The Analyst* **138**, 1903 (2013).

6. Wang, D. *et al.* Efficient Gene Delivery Based on Guanidyl-Nucleic Acid Molecular Interactions. *Adv. Funct. Mater.* **30**, 2004783 (2020).
